# Extraction and Purification of Biopolymers from Marine Origin Sources Envisaging Their Use for Biotechnological Applications

**DOI:** 10.1007/s10126-024-10361-5

**Published:** 2024-09-10

**Authors:** Duarte Nuno Carvalho, Cristiana Gonçalves, Rita O. Sousa, Rui L. Reis, J. Miguel Oliveira, Tiago H. Silva

**Affiliations:** 1 3B´S Research Group, I3B´s – Research Institute on Biomaterials, Biodegradables and Biomimetics of University of Minho, Headquarters of the European Institute of Excellence On Tissue Engineering and Regenerative Medicine, AvePark 4805-017, Barco, Guimarães, Portugal; 2 ICVS/3B´s – PT Government Associate Laboratory, Braga/Guimarães, Portugal

**Keywords:** Marine origin materials, Extraction methodology, Seaweeds, Polymer characterization, Blue biotechnology

## Abstract

Biopolymers are a versatile and diverse class of materials that has won high interest due to their potential application in several sectors of the economy, such as cosmetics, medical materials/devices, and food additives. In the last years, the search for these compounds has explored a wider range of marine organisms that have proven to be a great alternative to mammal sources for these applications and benefit from their biological properties, such as low antigenicity, biocompatibility, and biodegradability, among others. Furthermore, to ensure the sustainable exploitation of natural marine resources and address the challenges of 3R’s policies, there is a current necessity to valorize the residues and by-products obtained from food processing to benefit both economic and environmental interests. Many extraction methodologies have received significant attention for the obtention of diverse polysaccharides, proteins, and glycosaminoglycans to accomplish the increasing demands for these products. The present review gives emphasis to the ones that can be obtained from marine biological resources, as agar/agarose, alginate and sulfated polysaccharides from seaweeds, chitin/chitosan from crustaceans from crustaceans, collagen, and some glycosaminoglycans such as chondroitin sulfate and hyaluronic acids from fish. It is offered, in a summarized and easy-to-interpret arrangement, the most well-established extraction and purification methodologies used for obtaining the referred marine biopolymers, their chemical structure, as well as the characterization tools that are required to validate the extracted material and respective features. As supplementary material, a practical guide with the step-by-step isolation protocol, together with the various materials, reagents, and equipment, needed for each extraction is also delivered is also delivered. Finally, some remarks are made on the needs still observed, despite all the past efforts, to improve the current extraction and purification procedures to achieve more efficient and green methodologies with higher yields, less time-consuming, and decreased batch-to-batch variability.

**Graphical Abstract:**

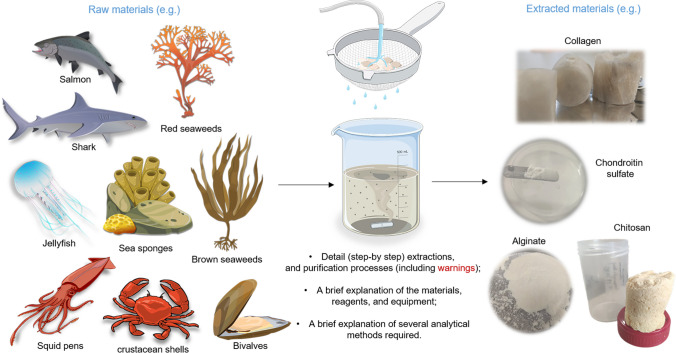

**Supplementary Information:**

The online version contains supplementary material available at 10.1007/s10126-024-10361-5.

## Introduction

A growing interest in biopolymers has been emerging in recent years, mainly their extraction/synthesis from natural sources, providing a wide range of valuable products due to their properties such as biocompatibility, biodegradability, or biomimetic properties. Conventionally, the industry has extracted diverse bioactive compounds from mammal organisms, such as bovines, pigs, chickens, and rabbits (Berillis 2015). Despite the efforts to use these products safely on human beings, they are associated with a series of risks, such as infections, immunogenicity, or rejection due to ethical reasons (Iswariya et al. 2017). To contradict these drawbacks, in today’s society, there is an increased concern in exploring marine biological resources such as marine sponges, jellyfish, seaweeds, fishes, crustaceous, cephalopods, and echinoderms (Ibanez and Cifuentes 2013; Andrade et al. 2013; Kim and Chojnacka 2015; Carvalho et al. 2021b). To attempt an eco-friendly approach, which has been widely promoted by the recent environmental education applied to the conversation of the marine ecosystems, strategies of biomass valorization under the circular economy concept, as the exploitation and the management of industrial wastes as by-products, should be addressed (Ferrario et al. 2017; Alves et al. 2017). In fact, 75% of the fish biomass caught is not properly used. Part of them is discarded back into the oceans or treated as by-products for feed or land fill, such as skin, fins, heads, eyes, swim bladders, and cartilage (Subhan et al. 2015; Hoyer et al. 2014; Abraham et al. 2008). For example, 60% of the total material weight from cod fillet industrial processing is considered a by-product (Moreira-Silva et al. 2016).

Currently, as mentioned above, the economy has arisen a substantial interest in marine products for several sectors, such as in areas of health-related applications such as (bio)medical materials/devices, green plastics, cosmetics, food additives, clothing fabrics, water treatment, pharmaceutical, biosensors, among others (Silva et al. 2016; Nithya et al. 2017). Many extraction methodologies have received significant attention for the obtention of diverse polysaccharides, proteins, and glycosaminoglycans (GAGs) to accomplish the increasing demands for these products. The most emphasis is given to agar/agarose, alginates, chitin, chitosan, ulvan, carrageenans, collagen, and some GAGs such as chondroitin sulfate (CS) or hyaluronic acid (HA) (Carvalho et al. 2021b; Silva et al. 2012b; Shen et al. 2021). However, an eco-friendly approach should be applied not only to the management of waste products but also to the procedures currently used to extract these bioactive compounds, such as using certain chemicals or solvents that are harmful to the environment. For this, there is a considerable concern in applying the 3R’s Policy (reduce, reuse, and recycle), i.e., in practical terms, recycling the waste generated and creating new innovative extraction alternatives (green methods) such as the use of ionic liquids as solutions that can substitute current chemical products used (Inman et al. 2022). Likewise, it is crucial operating all these procedures according to well-established regulations of Good Laboratory Practice (GLP) and further of Good Manufacturing Practices (GMP) in order to accomplish and ensure the good practices, safe, and quality of the laboratory environment materials, as well as authenticate its validity (Bornstein-Forst 2017; Organization 2009). In some cases, to obtain a (bio)medical grade, it is required to perform careful purification methodologies and several characterizations, such as analyzing heavy metal content and endotoxin levels, among others (10,993–1, 2016).

Furthermore, to be profitable for the economy, it is also essential and relevant to optimize the current methodologies for extracting these marine biopolymers to access a higher purity product and extraction yield, less consuming time, and more low-cost strategies. In fact, materials based on marine compounds are under study by several research teams due to their great potential and properties, such as lower antigenicity, anti-inflammatory, non-toxicity, non-mutagenic, non-carcinogenic, non-irritant, and anti-oxidant properties, as well as high hemocompatibility, safe biodegradability, and excellent mechanical properties (Carvalho et al. 2020a, b). For instance, marine collagen has been demonstrated to be promising for cosmetic applications such as the development of creams or gels due to anti-aging and anti-wrinkling factors and high moisturizing action, which is considered an excellent property to protect against UV radiation (Alves et al. 2017; Xhauflaire-Uhoda et al. 2008). The development of different tissue scaffolds (such as hydrogels, cryogels, and membranes) is widely used in biomedical and tissue engineering and regenerative medicine (TERM) approaches (Hoyer et al. 2014; Pozzolini et al. 2018). The use of alginate or chitosan composites for the adsorption of heavy metals, such as Pb, Cd, Cu, or Ni, for wastewater treatment (Nithya et al. 2017), among other commercial applications.

Several protocols and consequent modifications for obtaining these marine biopolymers can be found regarding the extraction methodologies available in the literature. However, many are specific to the selected biopolymer and the raw material, making it challenging to choose an adequate protocol, especially when some researchers initiate on extraction area. For this reason, the motivation to write this review article was based on a gap found in the literature for a piece of this typology and utility. The aim was to build a manuscript that unequivocally set together a compilation of detailed protocols (without specific equipment and easy handling) to extract several promising marine bioactive compounds and quickly get through the protocol choice phase. We focused on marine materials due to the expertise of the authors’ team. Thus, it gathers the acquired practical knowledge with vital literature content. Furthermore, this article intends to positively impact the scientific community that sought to produce these materials with so much potential in several fields.

## Marine Origin Biopolymers, Extraction Methods, and Characterization

### Collagen and Gelatin

#### Sources, Characteristics, and Biological Properties

**Fig. 1:**
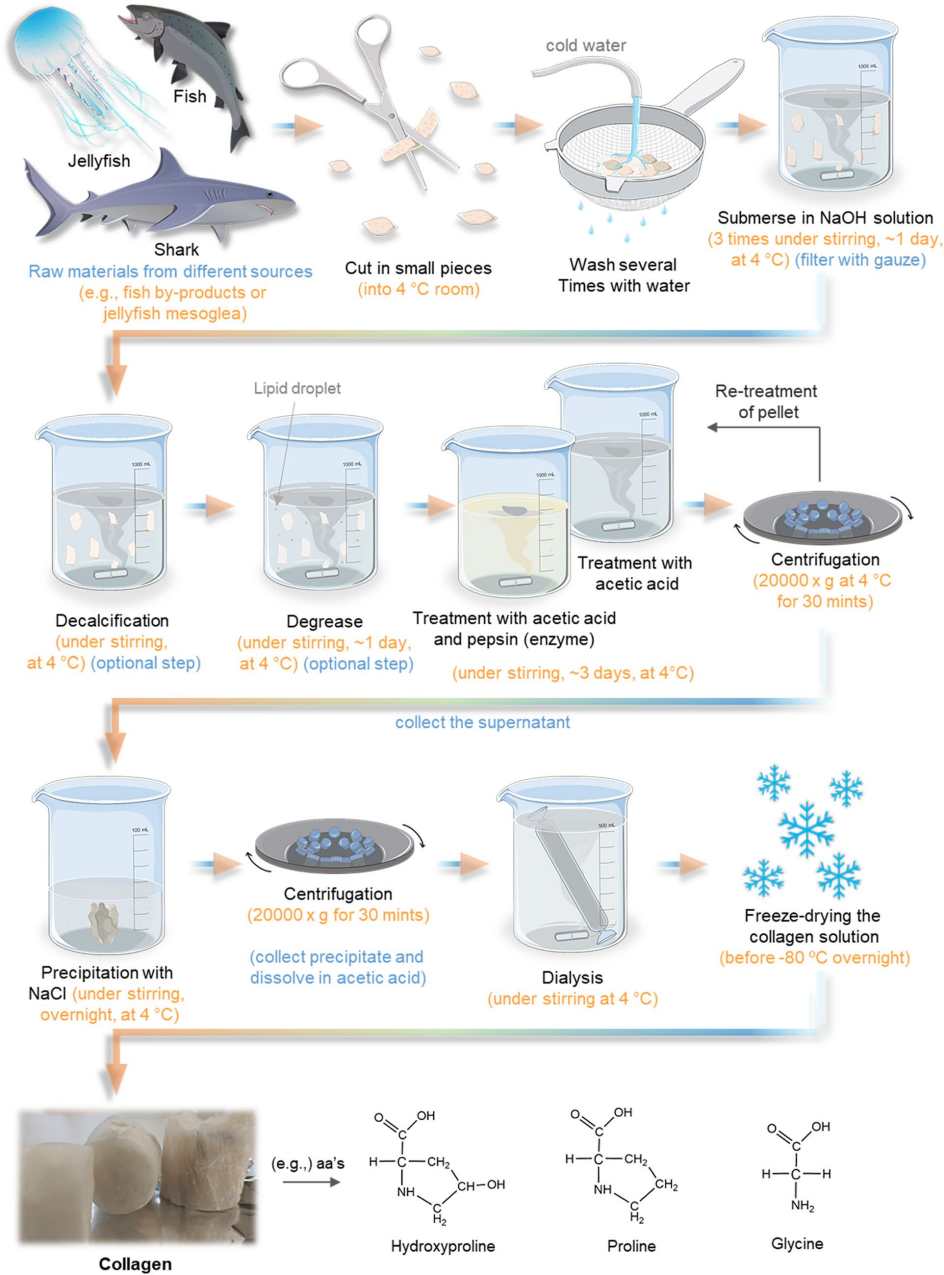
Schematic representation of acidic extraction methodology of collagen/gelatin and the sequential amino acid contents along with the collagen structure

Collagen is one of the most important structural proteins in the human body, accounting for around 30% of total protein weight, about a quarter of the total protein content of most animals (Shoulders and Raines 2009; Silva et al. 2012b, 2014). This protein has several key functions, from helping to form the extracellular matrix (ECM) of the skin’s dermis to its specific interaction with different receptors, being part of the signaling process (Tracy et al. 2014; Dwi Liliek and Hevi 2018; Arseni et al. 2018). Collagen also plays an important structural role in connective tissues (e.g., tendons, ligaments, bones), contributing to their molecular design, form, and mechanical properties and providing tensile strength and flexibility (Shoulders and Raines 2009; Alves et al. 2017; Muiznieks and Keeley 2013; Arseni et al. 2018). According to the literature, about 28 different types of collagens have been identified, although about 80 to 90% of the collagen in the body consists of the first three types (Jafari et al. 2020; Leon-Lopez et al. 2019). All collagens consist of amino acids bound together to form a triple helix, composed of 3 left-handed *α*-chain helices, assembled due to covalent and hydrogen bonding to form a right-handed supercoil that comprises the basic collagen unit (Ferraro et al. 2016; Brodsky and Persikov 2005). This triple helix is characterized by repetitions of triplets Gly-X–Y, a sequence of three amino acids in which glycine (Gly) is always present, and usually, the other two vary among several amino acids, often proline (Pro) and hydroxyproline (OHyp) (Ramshaw et al. 1998; Muiznieks and Keeley 2013; Prockop 2013), shown in Fig. 1.

Despite all collagen types sharing the triple helix characteristic, some structural differences give them specific functions, being grouped into two main classifications according to their capacity to form fibers: fibrillar (types I, II, III, V, and XI) and non-fibrillar collagen (Shoulders and Raines 2009). Determined by the disruption of the Gly-X–Y repeat of the *α* chain, instead of forming fibrils, these non-fibrillar collagens, such as type IV, form reticular networks in the ECM (Muncie and Weaver 2018; Jawad and Brown 2011). So, to promote the formation of collagen fibrils, the triple helix unites, self-assembles, and crosslinks through covalent and hydrogen bonds, which will also aggregate into semi-crystalline collagen fibers, which are essential for their own viscoelasticity, tensile strength of the tissue, and supporting cell growth (Berillis 2015; Jawad and Brown 2011).

**Fig. 2:**
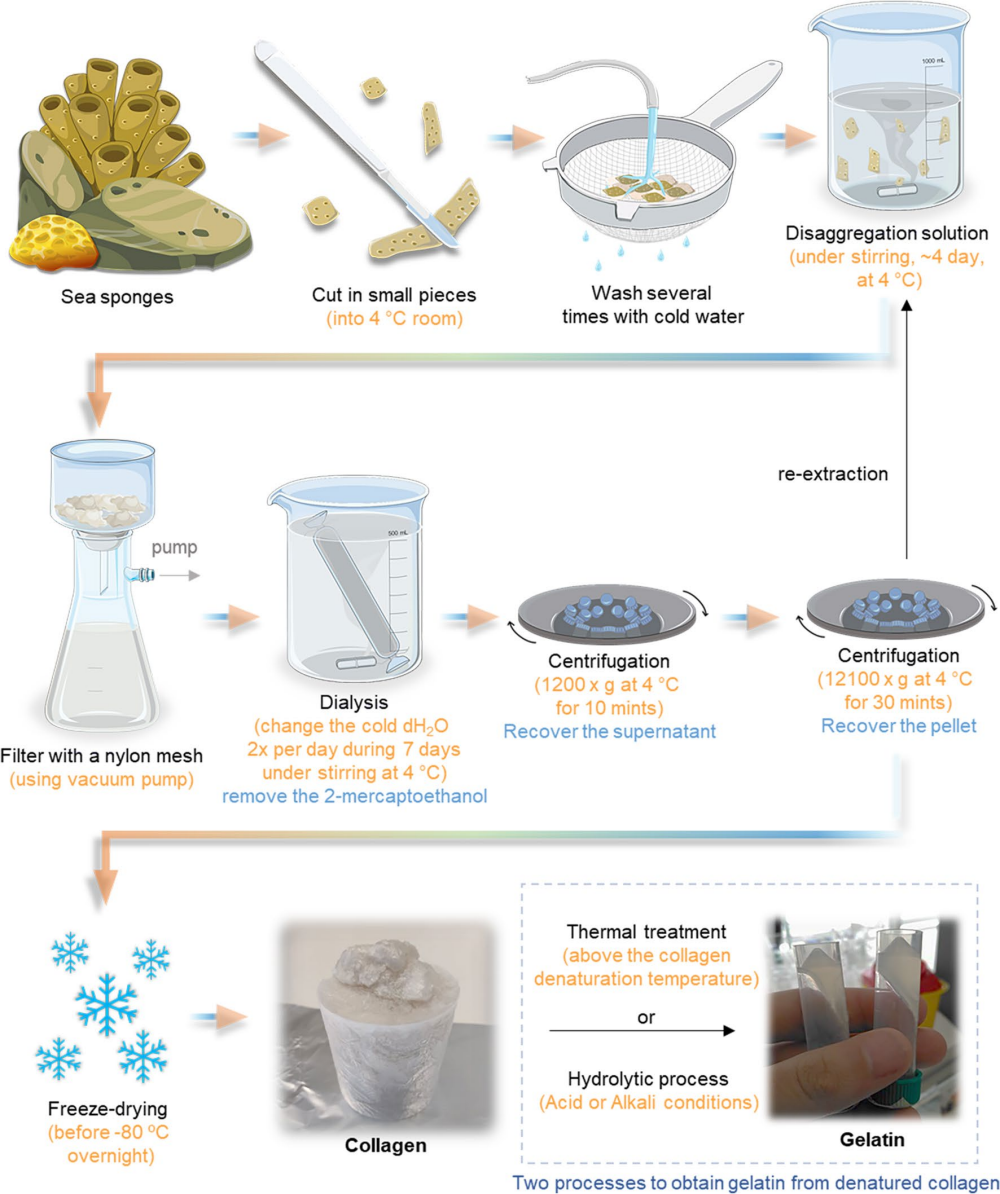
Schematic representation of alkaline extraction methodology of collagen and a short explanation of how gelatin can be obtained closely after a collagen extraction (by thermal treatment or hydrolytic procedure)

Depending upon the hydrolysis degree, collagen-based materials have been classified into undenatured collagen (around 300 kDa), gelatin (20–90 kDa), and collagen hydrolysates (2–9 kDa) (Van Vijven et al. 2012; Leon-Lopez et al. 2019; Li et al. 2005). The undenatured collagen obtained after isolation from a specific source (e.g., fish or bovine) with a high molecular weight. The gelatin is a collagen derivate that undergoes an irreversible chemical or thermal process until the denaturation temperature of the helix is reached, resulting in its loss of structure that origins the random coil conformation of the individual polypeptide chains due to bond breakage (Li et al. 2005), demonstrated on Fig. 2. In addition, collagen hydrolysates can be acquired, starting from gelatin, after applying an enzymatic treatment to divide it into smaller peptides with different sizes depending on the source (Aguirre-Cruz et al. 2020).

Due to their excellent biocompatibility and biodegradability, collagen and gelatine are the most sought-after materials preferred by the pharmaceutical, cosmetic, biomedical, and food industries (Ahmad et al. 2010; Carvalho et al. 2018a; Zhang et al. 2009). Traditionally, commercial collagen has been extracted from a variety of mammalian organisms (e.g., bovine). However, in the last years, mammalian collagen obtained from these sources has been associated with the risk of zoonotic diseases, such as bovine spongiform encephalopathy (BSE), transmissible spongiform encephalopathy (TSE), and foot-and-mouth disease (FMD), even due to ethical or religious restrictions (Alves et al. 2017; Silva et al. 2014). Therefore, new alternatives, such as using marine organisms, are being explored to obtain this biopolymer.

Marine resources have received more attention due to their availability, safety (free of zoonosis), environmentally friendly and simple processing techniques, less religious and ethical barriers, minor regulatory and quality control problems, low inflammatory response, and excellent metabolic compatibility (Silva et al. 2016; Silvipriya et al. 2015). Recent investigations have been concentrated on potential candidates for producing marine-origin collagen, such as fish (Silva et al. 2016; Sousa et al. 2020, 2019; Wang et al. 2007), jellyfish (Hoyer et al. 2014), sponges (Swatschek et al. 2002; Gokalp et al. 2020), mussels (Suhre et al. 2014; Rodriguez et al. 2017), squids (Cao et al. 2022; Jency and Dr. Manjusha 2022), sea-urchin (Ferrario et al. 2020; Marzorati et al. 2021), and sea cucumber (Cui et al. 2007; Senadheera et al. 2020). The possibility of collagen, in particular type I, being extracted from diverse fish parts can represent a valuable approach to valorizing its by-products (e.g., skin, bones, muscles, scales, and swim bladders) (Coppola et al. 2020; Shahidi et al. 2019). However, marine-derived collagen comprises a reduced proportion of hydroxyproline, lower cross-linking compatibility, and stability compared to mammal and avian collagen (Hickman et al. 2000). The collagen composition, stability, structure, and properties depend on the species, age, season, or habitat. Furthermore, the collagen extracted from warm water organisms has higher thermal stability than cold water species (Senadheera et al. 2020; Zain et al. 2020).

#### Overview of the Extraction Methodology

##### Acidic Solution Extraction Method

According to the existing literature, the procedures used to extract marine collagen seem like the ones for mammalians. The mainly used extraction techniques are based on the solubility of collagen in neutral saline solutions, acidic solutions, and acidic solutions with added enzymes (Schmidt et al. 2016). Nonetheless, it is necessary to consider that each kind of collagen has different characteristics between different species or individuals of the same species. In addition, the extraction yield was affected by the source of collagen, but also by the extraction technique used, the timing of the different reactions, the concentration of solutions used in the process, and the temperature of all isolation processes can also affect the collagen yield (Schmidt et al. 2016; Avila Rodriguez et al. 2018).

Marine collagen isolation needs to be defined and applied to this specific source, divided into three parts: preparation, extraction, and recovery, usually carried out at 4 °C to avoid protein denaturation (Avila Rodriguez et al. 2018; Coppola et al. 2020). Acid extraction is the most commonly used, mainly for fish by-products, jellyfish. The method starts by washing the raw material with distilled water, cut into small pieces, and chemically treated with sodium hydroxide (NaOH) to remove non-collagenous proteins, as represented in Fig. 1. In some cases, additional extra steps are essential, such as fat removal required for collagen extraction from codfish swim bladders (Sousa et al. 2019) (10% 2-propanol) or even the demineralization/decalcification that is required for the isolation of collagen from scales, cartilage, or bone (Jafari et al. 2020; Ahmed et al. 2020; Nagai and Suzuki 2000). Later, collagen isolation was achieved using an acetic acid solution, followed by centrifugation. Finally, the remaining biomass can be re-extracted following the same procedure. Furthermore, the collagen was precipitated by the addition of sodium chloride (NaCl) in Tris-HCl (pH 7.5), separated by centrifugation, purified by dialysis, and finally lyophilized (Sousa et al. 2019; Alves et al. 2017; Carvalho et al. 2018a). The extraction methods to obtain collagen using acidic methodology are presented in Fig. 1.

##### Alkaline Solution Extraction Method

In the case of marine sponges and echinoderms, the available methodologies are based on a barely basic solution and a chaotropic agent since the collagen isolated from these sources does not solubilize in acidic solutions (Silva et al. 2014). Indeed, extracted collagen is isolated as fibrils, which are difficult to solubilize, probably due to intramolecular crosslinking and higher glycosylation. Figure 2 represents a way of extracting collagen from sponges, which comprises the cleaning with dH_2_O, cutting into small pieces, treatment with disaggregating solution, filtration with nylon meshes, dialysis against dH_2_O, first centrifugation (10 min, 1200 × g) to remove impurities, followed by second centrifugation (30 min, 12,100 × g) to recover the collagen (pellet), froze at – 80 °C and freeze-drying (Gokalp et al. 2020). The extraction methods to obtain collagen using alkaline methodology are presented in Fig. 2.

Alternative methods are arising to comply with the principles of green chemistry and a more environmentally friendly alternative process, such as extracting a supercritical fluid using water acidified with carbon dioxide (Silva et al. 2016). This methodology comprises one single extraction step with soft operation conditions, avoids using an organic solvent, and increases the extraction yield (Barros et al. 2015; Sousa et al. 2020). Moreover, a potential alternative is aqueous solutions containing deep eutectic solvents to extract and purify collagen type I from Atlantic codfish (*Gadus morhua*) (Bisht et al. 2021). A step-by-step practical guide of extraction and purification procedures for the production of collagen (Tables S1 and S2) and a comprehensive inventory of the required materials, reagents, and equipment can be found in the supplementary information.

#### Analytical Methods for Polymer Characterization

The physicochemical properties of collagen and gelatin play a pivotal role in their diverse applications. Key factors to evaluate include their molecular weight, isoelectric point, and amino acid composition, as these properties influence their solubility, gelling capacity, and interactions with other molecules. Additionally, the thermal stability and rheological behavior of collagen and gelatin are crucial for processing and formulation considerations. Understanding their water-binding capacity, viscosity, and enzymatic susceptibility is vital in various industries, from food and pharmaceuticals to cosmetics and tissue engineering. Evaluating these physicochemical properties is essential to harness the full potential of collagen and gelatin in tailored applications. Some of these physicochemical characterizations are herein explained. (1) Amino acid content is acquired using chromatographic techniques, starting with protein hydrolysis and then by amino acid separation, identification, and quantification (Silva et al. 2014). The study of amino acid content permits the identification of collagen samples for the presence and quantification of characteristic amino acids, such as Gly, Pro, and OHyp. (2) Sodium dodecyl sulfate–polyacrylamide gel electrophoresis (SDS-PAGE) is an electrophoretic system, generally used as a method to separate proteins according to the molecular weight of denatured polypeptide chains, but also assessing the purity of a protein preparation (Righetti et al. 2001). This technique is used most to assess collagen source material purity and breakdown. Furthermore, Western blots can be employed to evaluate and identify the specificity of collagen type using monoclonal antibodies (Abraham et al. 2008). (3) Micro differential scanning calorimetry (micro DSC) is a versatile equipment used to measure a number of thermo-physical properties that permit the determination of the denaturation temperature (Td) of collagen and assess its thermal stability, which is correlated with the presence of OHyp in its structure (Carsote and Badea 2019). (4) Infrared spectroscopy (FTIR) enables the vibration study (stretching or bending) induced by infrared radiation, which changes the vibrational energy in the bond. Since different bonds and functional groups absorb at different frequencies, the transmittance pattern differs for each material. This analysis allows to detect of the collagen chemical structure and molecular bonds by studying the presence of the typical characteristic peaks: Amide A related to the intermolecular hydrogen bonding, which presents N–H stretching vibration, typically can be found within a range between 3000 and 3500 cm^−1^ (Belbachir et al. 2009; Sousa et al. 2020); Amide B can be detected into the range 3000–2870 cm^−1^, is associated with the asymmetrical and symmetrical stretch of CH_2_ groups (Tang et al. 2018); Amide I, typically located between 1650 and 1635 cm^−1^, representing the stretching vibration of C = O carbonyl groups of proteins, as collagen (Muthumari et al. 2016); Amide II is correlated to the C-N stretching combined with N–H bending vibration, CH_2_ bending and COO- symmetrical stretching, and it is found closed at 1540 cm^−1^. Finally, the Amide III is associated to N–H bending along with C-N stretching and C-O stretching and the peak can be observed near to 1240 cm^−1^ (Sousa et al. 2019). (5) Circular dichroism (CD) is a valuable device that uses the differential absorption of circular polarized light in an asymmetrical environment to assess the structure. It is mainly used to determine and characterize the secondary structure of proteins, and herein, particularly the helical nature of collagen. Additionally, this technique permits determining the denaturation temperature of proteins when recorded as a function of temperature (Greenfield 2006; Abraham et al. 2008). (6) Collagen or hydroxyproline quantification is an important analysis to assess the purity of the extracts, typically based on the OHyp contents (Colgrave et al. 2008). (7) Gel permeation chromatography (GPC) is a type of size-exclusion chromatography (SEC) that can separate analytes based on size and further determine the molecular weight (M_w_) of collagen and other biopolymers, as polysaccharides, expressed in Da or kDa using proper detectors. It is also essential to consider in this analysis the number average molecular weight (M_n_), the polydispersity index (M_w_/M_n_), and the intrinsic viscosity (IV) (Kasaai et al. 2000). To perform the analysis, collagen samples can be dissolved (1 mg mL^−1^) on the eluent with 0.15 M ammonium acetate (NH_4_OAc) and 0.2 M acetic acid (AcOH) solution (pH 4.5) and needs to be measured, for example, on NOVEMA column set (PSS—Polymer Standards Service, DE) since the collagen is a cationic polymer in acidic solutions (Carvalho et al. 2021a). (8) Mass spectrometry is a helpful analytical tool to measure the mass-to-charge ratio (m/z) of the molecules present in the sample. It can generally identify, quantify, and determine molecules’ structural and chemical properties by molecular weight determination (Rockwood et al. 2018; van Huizen et al. 2020). For example, according to the literature, the matrix-assisted laser desorption ionization time-of-flight (MALDI-TOF) mass spectroscopy is commonly used to analyze collagen samples, which allows the determination of molecular mass information and the identification of the sample and the presence of potential contaminants (Abraham et al. 2008). (9) Scanning transmission electron microscopy (STEM) is an extremely useful tool for visual confirmation of the morphology and physical state of the polymer surface using electron energy loss spectroscopy and high-angle annular dark-field imaging. The fibril structure and collagen organization can be assessed using this technique, TEM or AFM (Alexander et al. 2012). (10) Rheological analysis is the study of the flow and deformation of materials that provide information on how a given material reacts when subjected to a mechanical force and determine its behavior when subjected to different conditions, either in terms of stresses and shear deformation speed or as a function of temperature or other variables (Wilson 2018). (11) Glycosylation content of collagen can be assessed using, for example, a glycoprotein carbohydrate estimation kit (Pierce™—Thermo Scientific), which enables quantifying the amount of protein glycosylation to be measured as the percent of total purified protein mass. This kit has the advantage of being a simple and fast procedure, and it is a qualitative analysis since it can easily identify purified proteins as glycoproteins or even samples contaminated with sugars. It is also a semi-quantitative method since it can estimate the percentage of carbohydrate contents (w/w). This quantification is essential due it is known that the glycosylation process can influence protein behavior, including the formation, interactions, stability, and mobility (Roth et al. 2012), and (12) ion coupled plasma (ICP) analysis is atomic absorption spectroscopy used to identify and measure a range of chemical elements within the samples and study the presence of heavy metals. In addition, this technique allows for establishing the integrity and composition of the collagen samples. This method is beneficial for checking the absence of metal contaminants usually found in the sea.

### Chitin and Chitosan

#### Sources, Characteristics, and Biological Properties

Chitin has been considered the second most abundant natural polymer, after cellulose, being estimated to be produced annually in almost the same quantity as cellulose (Kumar 2000). This polysaccharide makes part of the organic matrix of exoskeletons of mollusks and arthropods, such as crustacean shells (e.g., crabs, shrimps), or even from endoskeleton, such as from squid pens. It is also present in insects, fungi cell walls, and algae (Kurita 2006; Raftery et al. 2016; Ahsan et al. 2018) or even in sponges (Zoltowska et al. 2019). However, chitin extraction is challenging in fungi due to its association with other polysaccharides such as cellulose, mannan, glucan, and polygalactosamine, making the isolation a complex methodology (Silva et al. 2012b).

Structurally, chitin is composed of a linear chain of (1 → 4) linked 2-acetamide-2-deoxy-*β*-d-glucopyranose units, being also designated as *N*-acetyl-d-glucosamine units (Zargar et al. 2015). In its extracted crude form, chitin presents a higher degree of acetylation (containing acetyl groups) and an ordered crystalline structure formed essentially by two allomorphs that leads the chain arrangement: *α* and *β*. The *α*-chitin is the most abundant and is characterized to contain an antiparallel arrangement and strong inter- and intramolecular hydrogen bonds (H-bonds), in which the presence of acetyl group units contributes to the stabilizing H-bond network. In contrast, *β*-chitin, being rarer, is characterized to have a parallel chain arrangement with weaker intermolecular hydrogen bonds (Kurita 2006). It is currently possible to differentiate these two allomorph chains using analytical methods such as infrared spectroscopy and solid-state nuclear magnetic resonance (NMR) spectroscopy, using X-ray diffraction (XRD) as an auxiliary characterization (Elieh-Ali-Komi and Hamblin 2016). Specifically, the alpha conformation is more prevalent in crustaceans, while the beta conformation is more frequently observed in cephalopods.

Chitosan is the most prominent derivative of chitin that can be obtained using alkaline conditions, being responsible for promoting the deacetylation reaction. During this process, acetyl groups that are present in chitin composition are removed, leaving behind the amino groups (-NH_2_) exposed, which can be protonated in mildly acidic solutions, making chitosan soluble in such conditions (while chitin is barely insoluble in most common solvents) (Gbenebor et al. 2017). In fact, the structural difference between chitin and chitosan is determined by the efficiency of the deacetylation process, i.e., higher or lower deacetylation degree (DD). Structurally, chitosan is composed of d-glucosamine (70–90%) and *N*-acetyl-d-glucosamine (10–30%) units, linked by *β* (1 → 4) glycosidic bonds, thus sharing a monomer with glycosaminoglycans such as hyaluronic acid that can be found throughout the body´s connective tissue, especially in cartilage ECM (Carvalho et al. 2020b).

In general, the DD has the capacity to affect the degradation rate and mechanical properties that include the rheological properties, as well the biological response in contact with cells, such as cell attachment, viability, and proliferation (Silva et al. 2012a). Besides that, chitosan naturally presents a higher positive electrical charge density which can be conjugated with anionic GAGs, proteoglycans, or other negatively-charged molecules, envisaging the creation of stable electrostatic complexes, for example, hydrogels (Silva et al. 2012b). Also, chitosan can be easily molded into different shapes and forms and contains natural biological properties such as non-toxicity, biocompatible, anti-inflammatory, antibacterial properties, biodegradable, and low-allergenic, that together increase their interest significantly for use in tissue engineering and biomedical application such as the treatment of neurodegenerative diseases or cartilage repair (Martins et al. 2014; Muraleedhara Kurup and Sumayya 2017).

#### Overview of the Extraction Methodology

##### Conventional Extraction Procedure

**Fig. 3:**
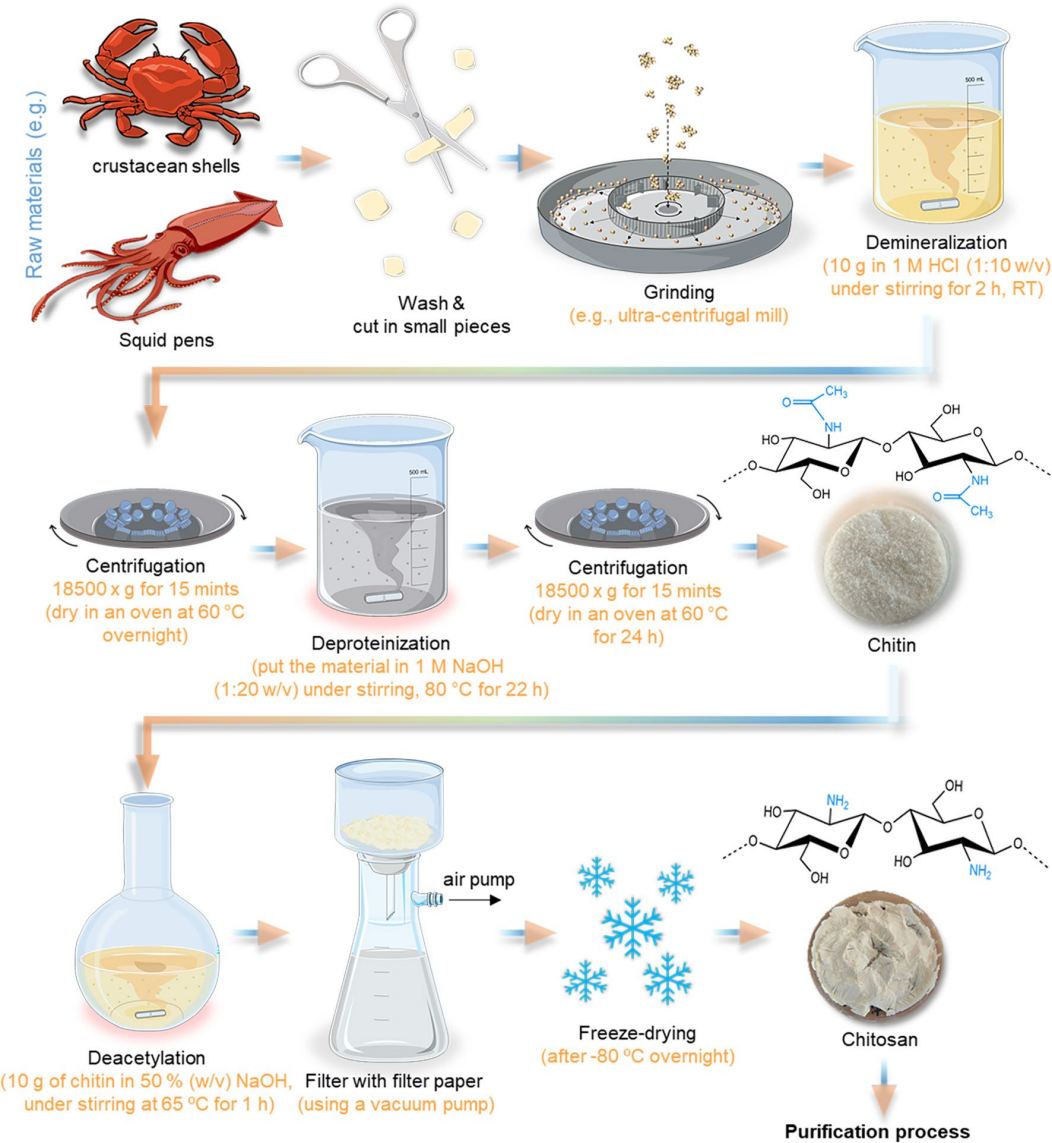
Schematic representation of the conventional extraction process of chitin from marine raw materials until its final conversion to chitosan. The typical model molecular structure of chitin and chitosan is represented (but in nature, they are slightly different: polymers with higher amounts of acetylated residues—chitin—are observed, while chitosan with a range of deacetylation degrees can be produced)

Conventionally, the chitin extraction protocol is divided into demineralization, deproteinization, and decolorization steps that can be carried out using chemical treatment (Percot et al. 2003) (Fig. 3) or, in some cases, can be used biological and/or enzymatic treatments to substitute the demineralization and deproteinization steps. In biological treatments, the fermentation process uses different species of bacteria and fungi, such as *Lactobacillus* sp., *Pseudomonas* sp., *Bacillus* sp., or *Aspergillus* sp. (Jung et al. 2006). In addition, decolorization is an optional process as it is only carried out when a colorless product is desired and the original material has inherent color. For this, acetone, NaOCl (sodium hypochlorite), 10% H_2_O_2_ (hydrogen peroxide) solution, or organic solvent mixtures can be used to remove the pigments that are expressed on the materials, being dependent on the final approach of these products (Maddaloni et al. 2020). As well, if the purpose is for example for biomedical or pharmaceutical areas the end-product needs to be highly purified to not cause serious side effects (Cheung et al. 2015). The conventional extraction methods to obtain chitin/chitosan are presented in Fig. 3.

##### Simpler and Faster Production Procedure

**Fig. 4:**
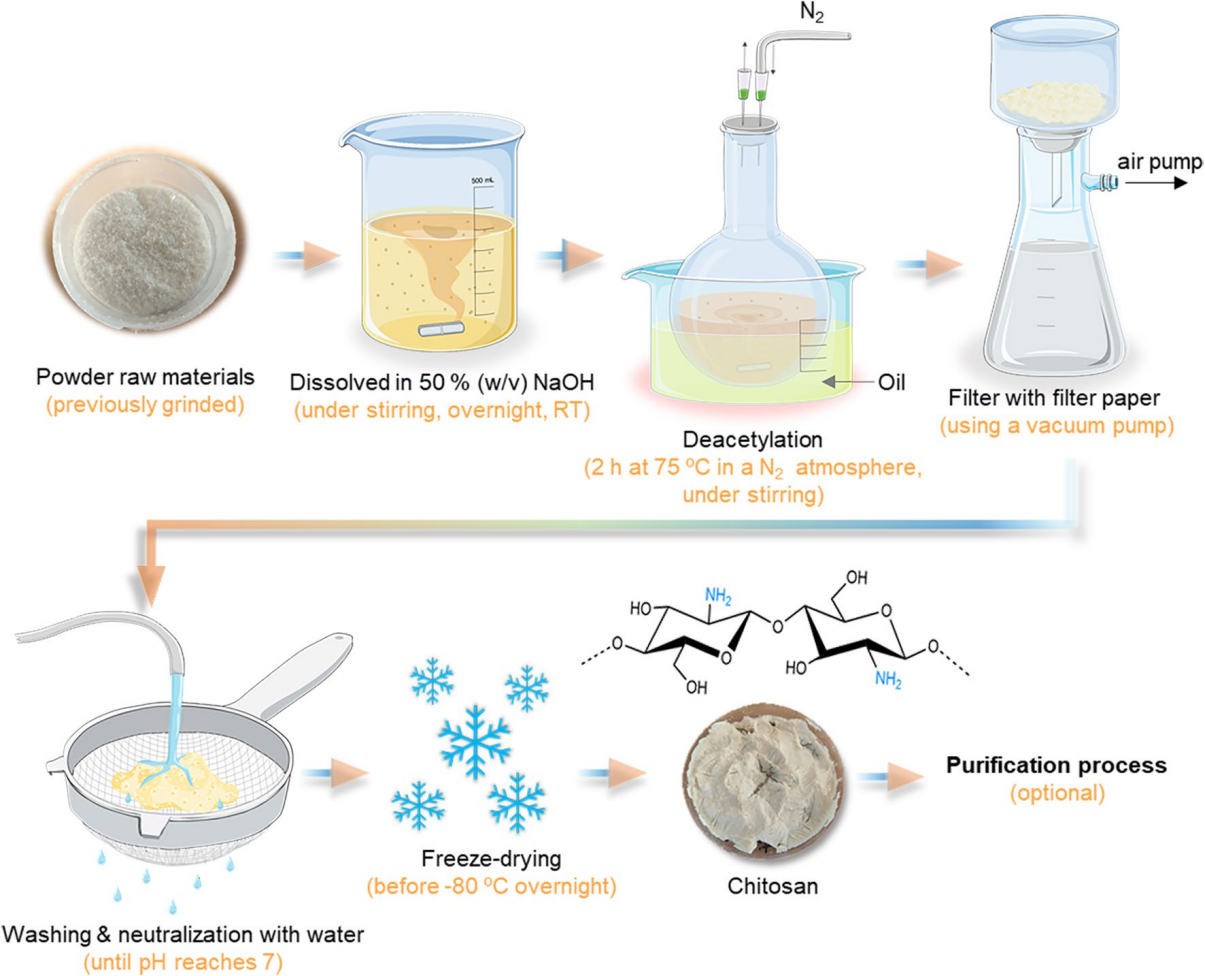
Schematic representation of chitosan production using a simpler and faster process in relation to the traditional methodology

Recently, a chitosan production approach was developed to be simpler and faster than the conventional extraction process (Fig. 4). This methodology can be performed using fewer steps, such as demineralization and deproteinization, which passes directly to deacetylation. In this case, the product is in contact with a nitrogen atmosphere (N_2_) that promotes deproteinization (López-Cebral et al. 2019). Unfortunately, in both extraction methodologies (conventional and faster), it is necessary to perform washing steps requiring huge amounts of water, which further limits the sustainability of the whole process. Additionally, this methodology is more suitable when using raw materials that are naturally colorless, such as squid pens. However, if the raw materials have color (such as crustaceous), the demineralization step should be applied to remove the pigments. The faster extraction methods to obtain chitin/chitosan are presented in Fig. 4.

##### Purification Process

The principle of this process is to obtain a high level of pure chitosan. A significant number of these contaminants come from the natural origin of the products and persist throughout the process.

**Fig. 5:**
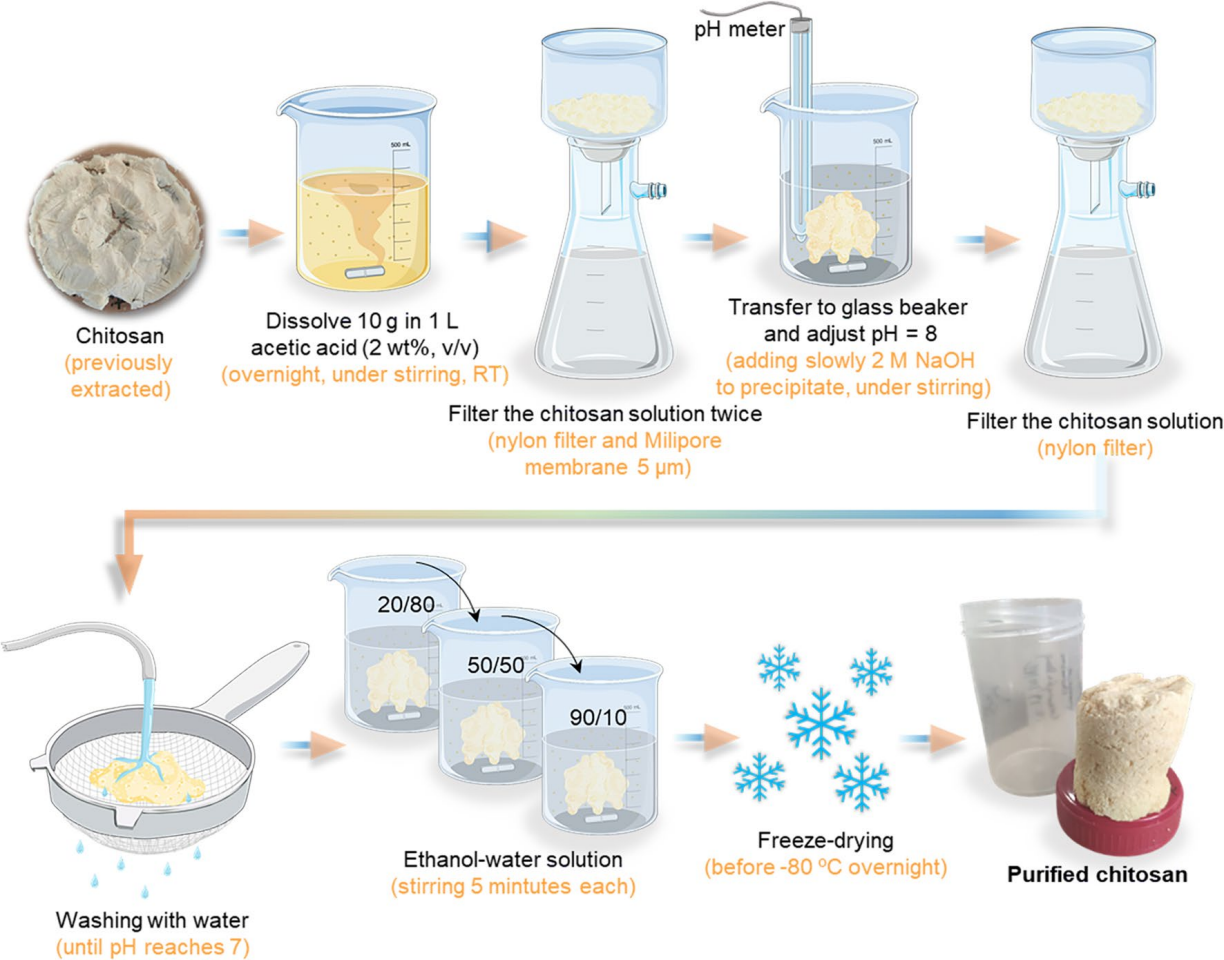
Schematic representation of purification methodology of chitosan

In general, the chitosan can be purified by dissolving in an acetic acid solution followed by precipitation (Fig. 5). However, like the extraction methodologies, this procedure has the disadvantage of requiring a considerable amount of water to neutralize the pH (Signini and Campana Filho 1999; Signini and Campana Filho 2001). The faster chitin/chitosan purification methodology is presented in Fig. 5.

A step-by-step practical guide of extraction and purification methodologies for production of chitin and chitosan (Tables S3, S4, and S5) and a comprehensive inventory of the required materials, reagents, and equipment can be found in the supplementary information.

#### Analytical Methods for Polymer Characterization

The physicochemical properties of chitin and chitosan are critical for their versatile applications. Evaluating their degree of deacetylation (DD) is crucial, as it influences solubility and biocompatibility, while understanding their molecular weight and distribution is essential for tailoring their mechanical and structural properties. For example, assessing their rheological behavior and viscosity aids in applications such as food additives, drug delivery, and tissue engineering. It is also important to evaluate the zeta potential and surface charge since they can affect their interactions with other substances. Overall, evaluating these physicochemical properties is fundamental in harnessing the potential of chitin and chitosan in diverse fields, including biomedicine, agriculture, and environmental science. Some of these physicochemical characterizations are herein explained. (1) Infrared spectroscopy (FTIR) is a common method applied to characterize polysaccharides like chitin and chitosan, especially to determine the degree of deacetylation and to confirm the homogeneity and purity by the presence of characteristic bands of each functional group. In chitin and chitosan samples, the most significant bands occur at wavenumbers of 3430–3450 cm^−1^ (OH stretching), 3255–3270 cm^−1^ (NH asymmetric stretching), 3100–3110 cm^−1^ (NH symmetric stretching), 1650–1655 cm^−1^ (CO stretching, amide I), 1590–1600 cm^−1^ (NH_2_ bending), 1550–1560 cm^−1^ (NH bending, amide II), and 1310–1320 cm^−1^ (CN stretching, amide III) (Carvalho et al. 2021a). Moreover, it is also possible to distinguish between the α and β-chitin, which the first having 2 peaks at approximately 1650 cm^−1^ while the β has only 1 peak. The percentage degree of deacetylation (DD %) can be calculated using Baxter’s equation (Eq. 1) with the intensity of the amide I band (~ 1655 cm^−1^) and the OH band (~ 3450 cm^−1^) obtained (El Knidri et al. 2016; Baxter et al. 1992).

1 $$\text{DD }\left({\%}\right)= \text{100} -\left[\left(\frac{{\text{A}}_{1655}}{{\text{A}}_{3450}}\right)\times {115}\right]$$

(2) X-ray spectroscopy or x-ray diffraction is a powerful analytical technique to obtain structural information, such as the polymorphic form of chitin crystallites as well the crystal structure/contents of chitin and chitosan. The patterns provide information about the periodic arrangement of atoms, resulting in an intensity diffractogram as a function of 2θ, which is an angle between the incident and diffracted beams (Stefanescu et al. 2012). The crystallinity index can be calculated using the relation of the peak intensities measured at 20° (*I*_110_) and 16° (*I*_am_) according to Eq. 2 developed by Segal et al. (Segal et al. 1959). Some authors defend that low crystallinity values are highly relevant for most applications such as biomedical fields, i.e*.*, if chitosan contains a lower crystallinity facilitates its solubility in acidic solutions, as it increases the accessibility of primary free amino groups present in their composition, and also because the degradability is greater the lower the crystallinity (Ioelovich 2014; Hahn et al. 2020).

2 $$\text{Crystallinity index }\left({\%}\right)=\frac{{I}_{110}-{I}_{\text{am}}}{{I}_{110}}\times 100$$

(3) Thermogravimetric analysis records the sample mass lost over time with the increase of the temperature. Typically, in polysaccharide samples, the thermograms exhibit two essential decomposition peaks. The first peak is a result of water evaporation that occurs at 50 and 110 °C, and the second peak is related to the degradation of the saccharide backbone, i.e., polymeric degradation and the decomposition of acetyl function that in chitosan samples is mainly observed between 300 and 400 °C (Paulino et al. 2006). Simultaneously, calorimetry measurements can be performed (e.g., differential scanning calorimetry—DSC), which provides additional information about the enthalpy values measured during the heating. (4) Elemental analysis can be performed in equipment such as X-ray photoelectron spectroscopy (XPS), also known as electron spectroscopy for chemical analysis (ESCA). This technique measures the presence of elements such as Carbon (C), Hydrogen (H), Nitrogen (N), and Oxygen (O), as well as the chemical and electronic state of the atoms within a material´s surface. For example, chitin and chitosan samples are used to determine the percentage of the degree of acetylation (DA) and deacetylation (DD) using Eqs. 3 and 4.

3 $$\text{DA }\left({\%}\right)\text{=}\left[\frac{\frac{\text{C }\left({\%}\right)}{\text{N }\left({\%}\right)}-\text{5.14}}{1.72}\right]\times {100}$$

4 $$\text{DD }\left({\%}\right){=}\left[\frac{\text{6.89}-\frac{\text{C }(\%)}{\text{N }(\%)}}{1.72}\right]\times {100}$$

(5) Gel permeation chromatography (GPC) or dynamic light scattering (DLS). The molecular weight (M_w_) and the intrinsic viscosity (IV) of chitosan can be assessed by GPC. For this, chitosan samples can be dissolved (1 mg mL^−1^) on the eluent with 0.15 M ammonium acetate (NH_4_OAc) and 0.2 M acetic acid (AcOH) solution (pH 4.5). They can be measured, for example, using a NOVEMA column set (PSS—Polymer Standards Service, DE) since the chitosan is a cationic polymer (Carvalho et al. 2021a). On the other hand, the DLS technique, also recognized as photon correlation spectroscopy or quasi-elastic light scattering, can measure macromolecules' molecular weight in solution, assessed by particle sizes (Carvalho et al. 2018b). (6) NMR spectroscopy can be used to investigate the electronic environment of single atoms and the interaction between neighbor atoms present in sample composition measured by the potential of ^1^H NMR, ^13^C NMR, and.^15^N NMR spectroscopy, being possible to use the sample in solid-state or solubilized. For this analysis, chitosan can be dissolved in deuterium oxide (D_2_O) and deuterium chloride (DCl), a minimum of 1 mg/mL. In general, the NMR spectrum can be strongly used to determine the deacetylation degree, the distribution of acetyl groups, the determination of impurities, and the cross-linkages of chitin and chitosan (Vårum et al. 1991; Heux et al. 2000). To understand the efficacy of the deacetylation step, the percentage of deacetylation degree (DD) is calculated using the following Eq. 5. where A_1_ is the protons integral values of positions C2–C6 on the sugar ring, and A_2_ is the protons integral values of the three N-acetyl protons of nGlcNAc (Carvalho et al. 2020b).

5 $$\text{DD }\left({\%}\right)\text{=}\left[1-\frac{6\times {\text{A}}_{2}}{3\times {\text{A}}_{1}}\right]\times {100}$$

### Fucoidan

#### Sources, Characteristics, and Biological Properties

**Fig. 6:**
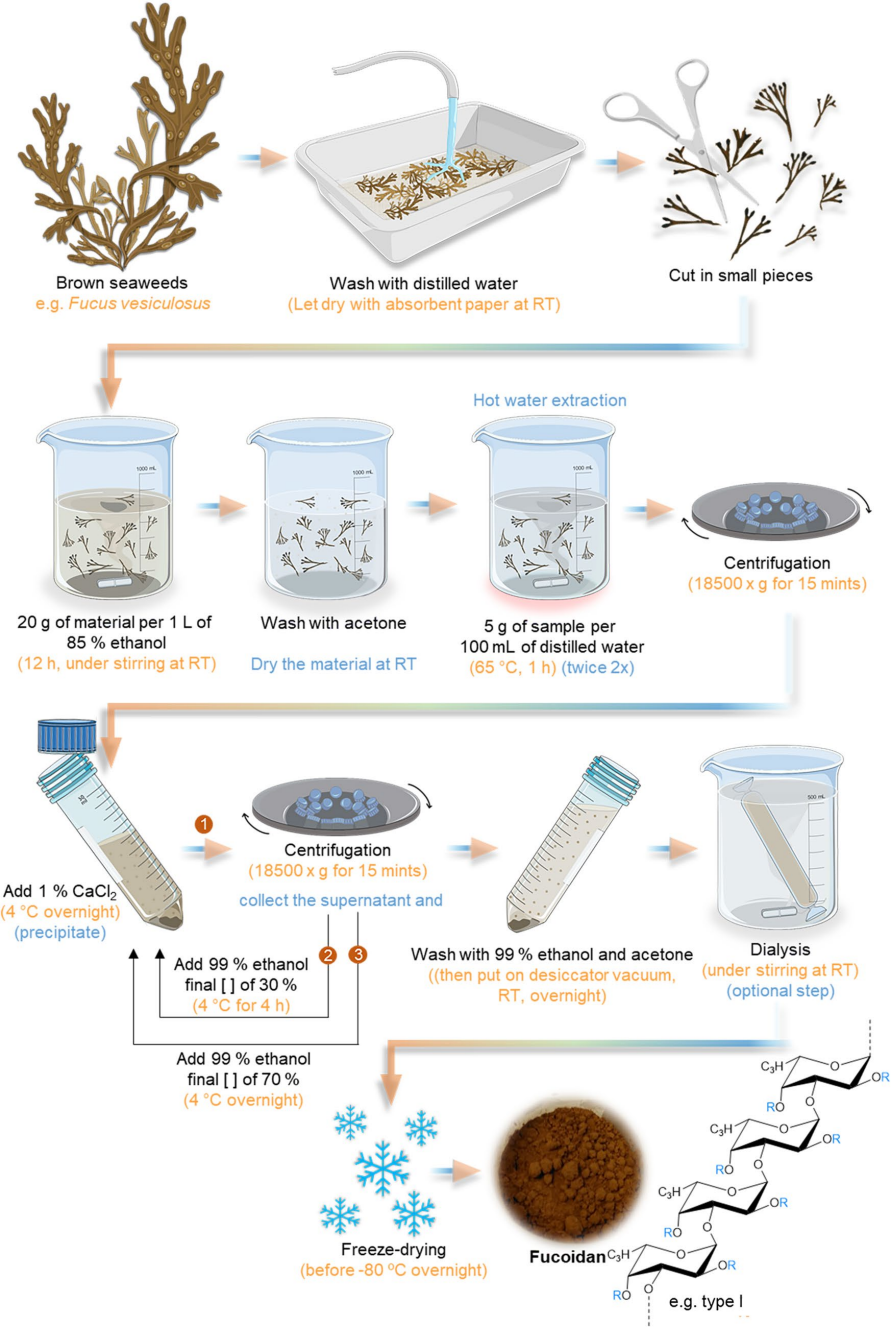
Schematic representation of hot water extraction methodology of fucoidan. It also demonstrates the molecular structure of Fucoidan type I, which essentially consists of repeated (1–3)-*α*-l-fucopyranose. R represents the variations by the different groups, which can be *α*-lfucopyranose, *α*-d-glucuronic acid, sulfate groups, and other sugars

**Fig. 7:**
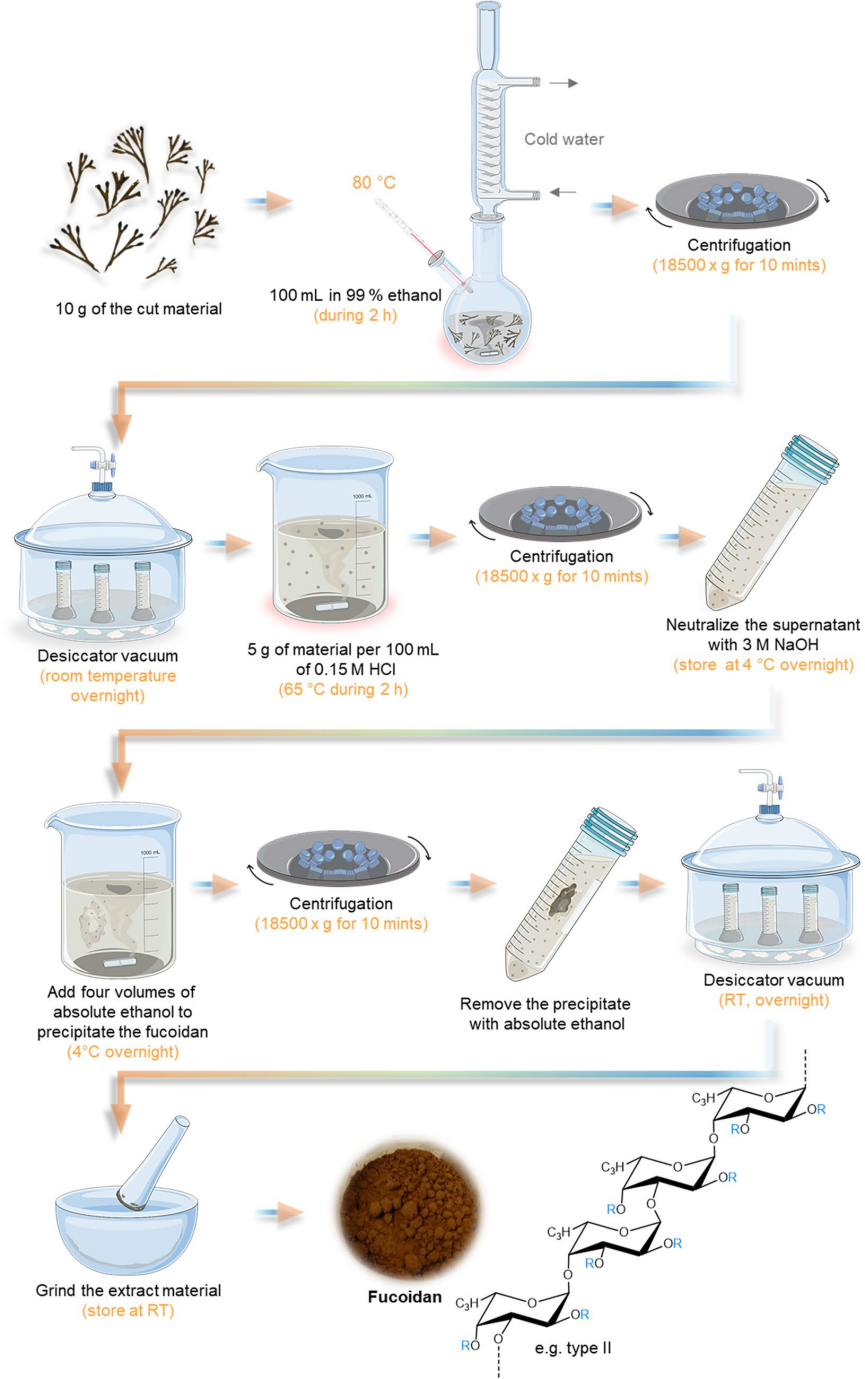
Schematic representation of acidic extraction methodology of fucoidan. It is also demonstrated the molecular structure of Fucoidan type II that is composed of alternating (1–3) and (1–4)-*α*-lfucopyranose). R represents the variations by the different groups, which can be *α*-lfucopyranose, *α*-d-glucuronic acid, sulfate groups, and other sugars

Fucoidan is an anionic polysaccharide from marine origin found in the tissue wall and intracellular spaces of different brown seaweeds species such as *Laminaria* sp., *Ascophyllum* sp., *Bifurcaria* sp., *Undaria* sp., and *Fucus* sp. (Wijesinghe and Jeon 2012; Senthilkumar et al. 2013). Structurally, fucoidan is composed of a backbone of fucopyranose (or fucose), often sulfated, together with side chains of uronic acids, and other monosaccharides in smaller quantities like d-xylose, d-galactose, d-mannose, glucose, arabinose, or l-rhamnose (Sinurat et al. 2015; Fletcher et al. 2017). Moreover, the fucoidan structure can be divided into two groups that largely depend on their source; type I have a central chain composed of (1–3)-*α*-l-fucopyranose residues (chemical structure represented in Fig. 6), and type II is composed of alternating and repeating (1–3) and (1–4) *α*-l-fucopyranose residues (Fig. 7) (Senthilkumar et al. 2013; Wu et al. 2016).

Recently, some studies demonstrated diverse biological activities of fucoidan and its lower molecular weight oligosaccharides derivatives that have a huge potential health benefit, enabling its use as pharmaceuticals, cosmetics, and nutraceuticals products, as well as biomedical and therapeutic applications, being included for TERM approaches (Reys et al. 2016; Oliveira et al. 2017). In this order, fucoidan can contain anti-coagulant properties, antithrombotic, antiangiogenic, anti-inflammatory, anti-tumor, antiviral, antihyperlipidemic, antihyperglycemic, antihyperlipidemic, immunomodulatory, contraceptive, antioxidant, and also protection effects of the digestive tract, as well as wound healing properties (Silva et al. 2012c; Fletcher et al. 2017; Flórez-Fernández et al. 2018). Unfortunately, these properties are not present in all fucoidan extracts, and their correlation to fucoidan’s chemical and structural features is not fully understood, creating multiple extract variations (Oliveira et al. 2017). Additionally, sulfated groups present in polysaccharides as fucoidan have considerable potential for TERM applications, especially for the treatment of articular cartilage tissue since they are considered non-toxic, biodegradable, water absorber, an inhibitor of arthritis, promoter of chondrogenic differentiation of stem cells, and have the ability to sequester growth factors that can increase the regeneration of damaged tissues (Karunanithi et al. 2016; Portocarrero Huang et al. 2017; Silva et al. 2012d). However, this may depend on structural differences, such as the number of sulfate groups and sugars.

#### Overview of the Extraction Methodology

##### Hot Water Extraction Method

The hot water extraction method is the most frequently used to extract fucoidan from algae and is the greenest protocol to date. This methodology was adapted by Yang et al. (2008) and had the advantage, compared with other extraction methods, of producing high-quality fucoidan, maintaining their stability and charge, high yield, and conserving the natural bioactivity and properties (Ragan and Craigie 1980). This process consists of using a pre-treatment with alcohol and acetone to remove lipids, salts, proteins, and color pigments and treatment with calcium chloride (CaCl_2_) to remove insoluble components that provide fucoidan a higher purity (Rani et al. 2017). In addition, this step can effectively remove alginate in brown algae cell walls, enabling a co-extraction using the precipitate after the treatment with CaCl_2_ solution (Dobrincic et al. 2020). The entire procedure is presented in Fig. 6.

##### Acidic Extraction Method

Different acids can be used for acidic fucoidan extraction; hydrochloric acid is the most used. However, compared with water extraction, the process yield is smaller (Rani et al. 2017). Likewise, the water extraction method can offer the whitest colored fucoidan, while the acid extraction provides a brownish powder (Lee et al. 2006). In addition, they also had the advantage of requiring fewer steps to extract the fucoidan, highlighting the no dependence on the freeze-drying step to obtain a dry extracted material which requires less time. The acid extraction methodology is demonstrated in Fig. 7.

##### Salt Extraction Method

**Fig. 8:**
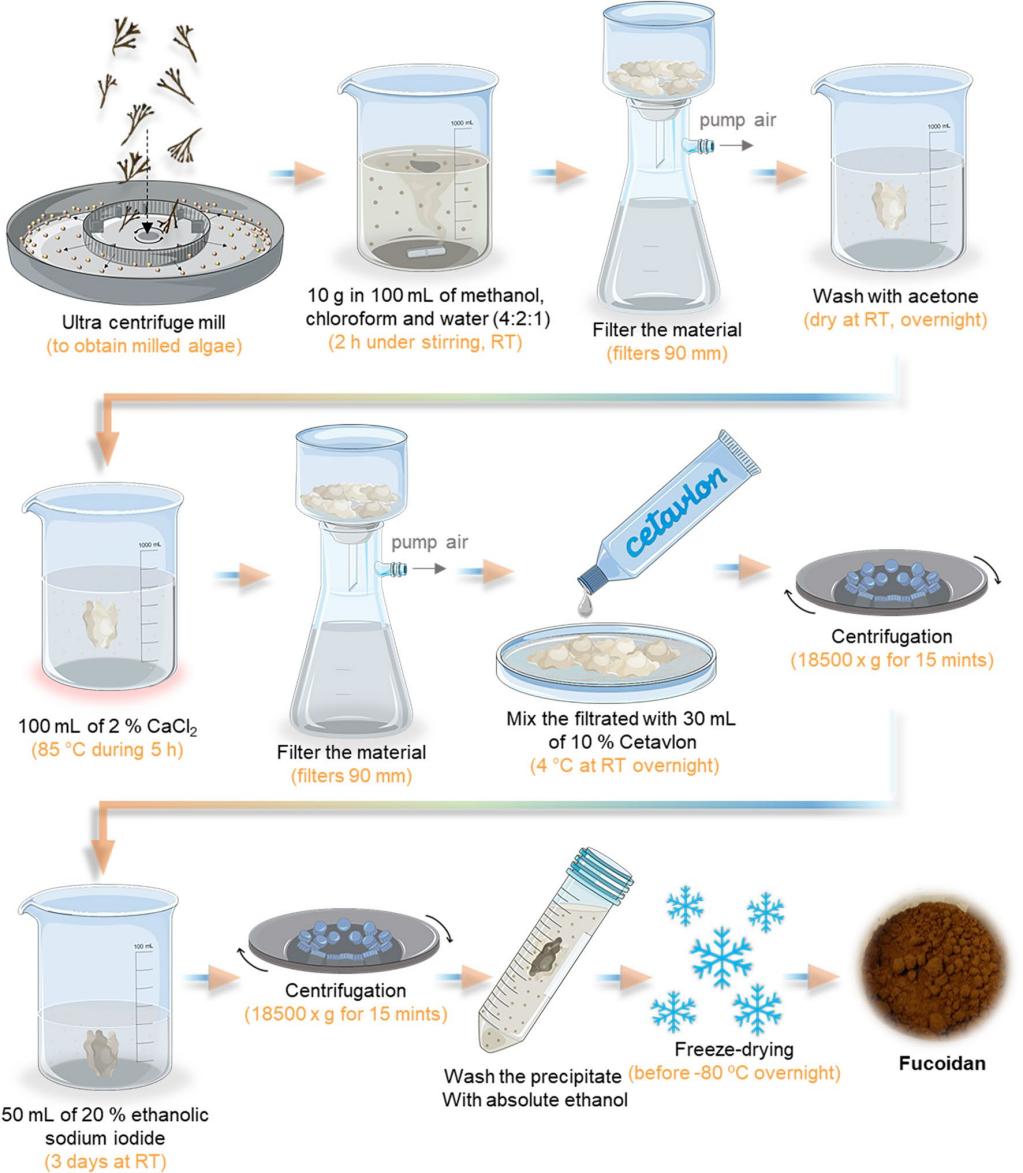
Schematic representation of salt extraction methodology of fucoidan

The use of calcium chloride (CaCl_2_) in this methodology effectively removes alginate, which is present in brown algae cell walls. During the extraction process, the solution containing CaCl_2_ enables extraction and dissolution of fucoidan and sodium alginate, making the process more effective with higher temperature and mechanical agitation. When sodium alginate gets in contact with the calcium ions, sodium ions are replaced in the polymer structure, forming solid calcium alginate. The ion replacement turns the alginate insoluble in water, being easily separated from the fucoidan and with a higher percentage of purity (Dobrincic et al. 2020). Indeed, to obtain high quality fucoidan should contain less than 0.1% of contaminated proteins (Kawamoto et al. 2006). To better isolate the fucoidan from the extract, a cationic detergent called hexadecyltrimethylammonium bromide (CETAVLON or CTAB) forms salts with the negative charges of the fucoidan. These salts are highly insoluble in water, promoting their precipitation (Dobrincic et al. 2020; January et al. 2019). However, the high quality material has a price since this methodology can affect the yield, i.e., obtaining a lower yield of crude fucoidan comparing the water and acidic method (Bilan et al. 2002). The salt extraction methodology is represented in Fig. 8.

A step-by-step practical guide of extraction and purification methodologies for the production of fucoidan (Tables S6, S7, and S8) and a comprehensive inventory of the required materials, reagents, and equipment can be found in the supplementary information.

#### Analytical Methods for Polymer Characterization

Fucoidan possesses several vital physicochemical properties that necessitate assessment for its various applications. Key factors include its molecular weight, which influences its bioactivity, solubility, and potential therapeutic effects. The degree of sulfation plays a significant role in determining its anticoagulant and antiviral properties. Fucoidan’s structural characteristics, such as its branching and conformation, affect its interactions with biological molecules. Its charge density, determined by the sulfate content, impacts its biological activity and stability. Additionally, the assessment of fucoidan’s purity and composition is essential for ensuring its consistent quality in industries like pharmaceuticals, cosmetics, and nutraceuticals. Some of these physicochemical characterizations are herein explained. (1) FTIR. In infrared spectroscopy analysis, the basic backbone of fucoidan contains a broadband approximately at 3500 cm^−1^ that corresponds to hydrogen bonding O–H group stretching vibrations, and in some samples, a weak signal appears around 2900 cm^−1^ that is characteristic of C-H stretching vibration (Chale-Dzul et al. 2014). Usually, an expressed band at 1700–1600 cm^−1^ is representative in all fucoidan samples, corresponding to the asymmetric elongation of the O-C-O vibration, indicating the absorbance of uronic acid (Hifney et al. 2016). The signal around 1400 cm^−1^ corresponds to the symmetric stretch vibrations of COO^−^ and the stretch vibrations of C-O within -COOH. The signal close to 1260 cm^−1^ is attributed to the asymmetric stretching of S = O, and the small band around 840 cm^−1^ suggests a pattern of sulfate substitution (C-S–O) at the C-4 position (axial substitution of *α*-linked l-fucopyranose. Both signals are essential features to confirm the presence of sulfate groups that are correlated to the bioactive properties of this polysaccharide (Mähner et al. 2001; Hifney et al. 2016). Additionally, the presence of two bands around 530–560 and 600–680 cm^−1^ is attributed to asymmetric and symmetric O = S = O deformation of sulfates, being also used to detect sulfate groups (Jo and Choi 2014; Fernando et al. 2017). (2) NMR. The chemical structure of fucoidan can be addressed by ^1^H NMR spectroscopy, using the deuterated water (D_2_O) as a solvent, a minimum of 1 mg/mL. Typically, the values detected of the chemical shifts are the isolated regions of methyl (H-6) protons, acetyl protons, ring (H-2 to H-5) protons, and anomeric protons. (3) Fucoidan composition. The composition of fucoidan can be analyzed by gas chromatography with the flame ionization detector (GC-FIC). GC-FID is used to perform compositional analyses of various organic compounds due to their high sensitivity to detect carbon amounts in the sample. The samples are burned in a hot, hydrogen-air flame during the analytical process, which forms carbon ions. The total amounts of ions detected are directly proportional to the amounts of carbon present in the sample, which can comprise a considerable amount of sugars (expressed in percentage %), namely galactose, xylose, traces of rhamnose, arabinose, mannose, glucose, among others. (4) Molecular weight by GPC. The size of the polysaccharide chains can be assessed by determining their molecular weight, being expressed in Da or kDa. Likewise, in analytical methods in chitosan, this technique can give additional information such as the number average molecular weight (M_n_), the polydispersity index (M_w_/M_n_), and the intrinsic viscosity (IV). For this analysis, fucoidan can be dissolved (1 mg mL^−1^) in PBS-buffered saline (0.01 M phosphate buffer, 0.0028 M potassium chloride, and 0.136 M sodium chloride, pH 7.4 at 25 °C, Sigma-Aldrich) and 0.05% w/v NaN_3_, and needs to be measured, for example, on SUPREMA column set (PSS—Polymer Standards Service, DE) since this polysaccharide is an anionic polymer. (5) Protein contents. Micro BCA protein assay kit (e.g., Thermo Scientific, USA) can be used to quantify the amounts of protein present in the fucoidan sample. For this quantification, the optical density of the standard curve and samples needs to be read at 562 nm in a microplate reader. (6) Sugar contents. For this quantification can be used the phenol–sulfuric acid assay or also called as Dubois method, since it is a simple acid-catalyzed condensation reaction developed by Dubois and their collaborators (Dubois et al. 1951) that is commonly employed for the determination of total sugar concentration in carbohydrates. (7) Uronic acid contents. To estimate the uronic acid contents in fucoidan samples, the method of Bitter and Muir (1962) can be used (Bitter and Muir 1962), which is a modification of the original procedure developed by Dische (1947). This modified procedure has less interferences, stable color formation, and the reaction is faster than the original methodology. To quantify, the absorbance should be measured at 530 nm using a spectrophotometer. (8) Sulfate contents. The measurements of sulfate in fucoidan samples can be carried out by turbidimetry, resulting from the formation of precipitates upon the addition of BaCl_2_, as Ba^2+^ interacts strongly with ester sulfates in fucoidan (Dodgson and Price 1962), whereby sulfate contents are estimated turbidimetrically as BaSO_4_. For this, the absorbance needs to be measured at 420 nm using a spectrophotometer. (9) Fucose contents. To determine the amounts of free fucose, it is possible to use the cysteine-sulphuric acid method for methyl pentoses (Dische and Shettles 1948). The optical density of the standard curve and the samples should be read at 396 nm and 427 nm using a spectrophotometer. The absorbance values are calculated using the following Eq. 6:

6 $$\text{Absorbance}=\left(\text{A396}\text{ nm}-\text{A427}\text{ nm}\right)$$

### Carrageenan

#### Sources, Characteristics, and Biological Properties

Carrageenan represents a family of linear sulfated polysaccharides, structurally is an anionic polymer, and can be extracted from certain species of red seaweeds (Rhodophyta—Class Gigartinales), mainly from *Chondrus crispus*, *Eucheuma cottonii*, *Gigartina* sp., and *Spinosum* sp. (Carvalho et al. 2020a). In general, the red algae are composed of carrageenans that occupy between 60 and 80% of the cell walls, 10–47% of proteins (high levels in late winter and lower during the summer season), floridean starch, and metabolites such as vitamins, essential oils, and phenols (Silva et al. 2012b). However, it is highly dependent on the species, season, location, and growth conditions (BeMiller 2019; Alba and Kontogiorgos 2018; Jhurry et al. 2006). The backbone of carrageenan is derived from galactose which consists of alternating units of 3-linked* β*-d-galactopyranose (G-unit) and 4-linked *α*-d-galactopyranose (d-unit) or 4-linked 3,6-anhydro-*α*-d-galactopyranose (DA-unit), thus forming disaccharide repeat units (Alba and Kontogiorgos 2018). In this order, it is estimated that the major constituents of the carrageenan structure are galactose and sulfate, followed by other carbohydrate residues such as xylose, glucose, and uronic acids, and in some samples can be present substituents like methyl ethers and pyruvate groups (Alba and Kontogiorgos 2018; Guan et al. 2017; Jhurry et al. 2006).

**Fig. 9:**
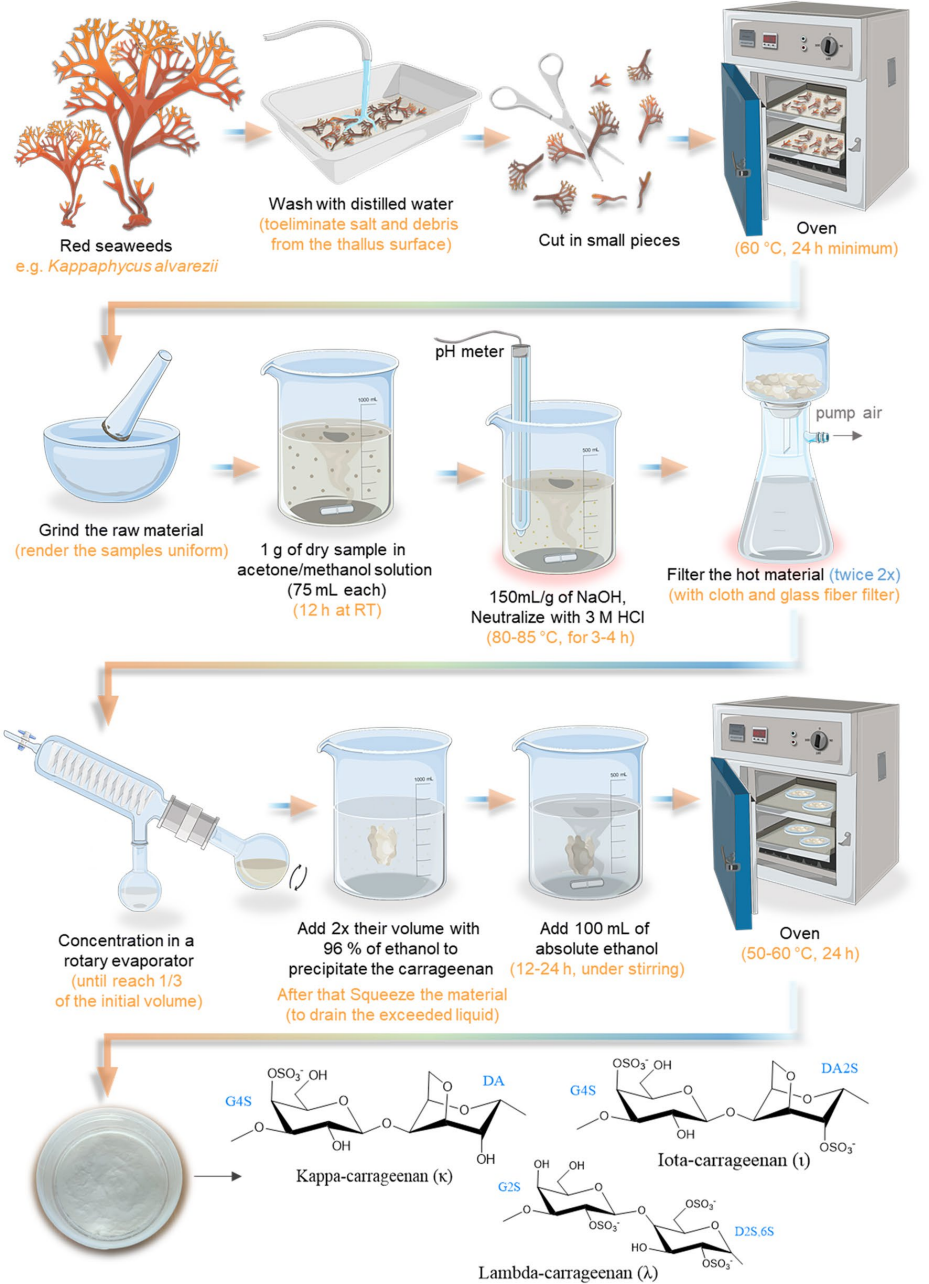
Schematic representation of alkaline extraction methodology (laboratory scale) of carrageenan. It also represents the molecular structures of different types of carrageenan (κ, ι, and λ)

According to structural variations, carrageenans can be divided into different families that are distinguished based on their primary structure and the number/position of the sulfate groups per basic disaccharide unit (Fig. 9). The most relevant types of carrageenan are kappa (κ), lambda (λ), and iota (ι), while mu (μ), nu (ν), theta (θ) are biological precursors of κ and ι, respectively (Alba and Kontogiorgos 2018; Guan et al. 2017). Therefore, it is estimated that κ and ι—carrageenan can be extracted from algae *Kappaphycus alvarezii* and *Eucheuma denticulatum*, typically together, while the λ—carrageenan can be obtained essentially from algae *Gigartina skottsbergi* and *Sarcothalia crispata* (Alba and Kontogiorgos 2018).

Recently, carrageenans have been investigated due to several biological activities (dependent on the carrageenan type and by molecular weight), ranging from anticoagulant and antithrombotic to immunomodulatory, antioxidant, antiviral, antitumor effects, and anti-inflammation (except the λ-carrageenan, which are used as an inflammatory inductor) (Guan et al. 2017; B.S. Albuquerque et al. 2016; Liang et al. 2018). Besides, its interest is increasing in several areas, from pharmaceuticals for drug administration approaches (Li et al. 2014), to environmental and biosensor applications (Esmaeili et al. 2017; Ooi et al. 2015), as well as tissue engineering (Popa et al. 2015).

#### Overview of the Extraction Methodology

Currently, in laboratory and industrial conditions, due to the achieved yield, most carrageenan is extracted from Kappaphycus alvarezii and Eucheuma denticulatum. Initially, when the extraction methodology was developed, carrageenan was obtained, mainly *Chondrus crispus* (Pereira et al. 2017). On a laboratory scale, the process starts with immersing the raw material in water to clean and remove traces of sand, shells, and other foreign matter as dead materials (this procedure is also the same used in industry). Then, a pre-treatment with acetone and ethanol is made to eliminate the organo-soluble fraction (Zinoun and Cosson 1996), and a treatment with NaOH in a hot bath to disaggregate the carrageenan from the other components of the ECM and to convert the units of *α*-d-galactopyranose-6-sulfate monomers into hydrophobic units of 3,6-anhydro-*α*-d-galactopyranose. Finally, the carrageenan must be precipitated and then dried in an oven. The procedure is demonstrated in Fig. 9.

In industrial conditions, after the algae are clean and dried, it is necessary to previously determine some characteristics of the dried raw material, such as the presence of humidity, sand, salts, epiphytes, and know the polymer that is intended to extract in order to allow the adequate adjustments on the methodology to obtain a successful extraction of carrageenan. For example, it is known that the λ-carrageenan can be dissolved at low temperatures (~ 15–20 °C) while the κ- and ι-carrageenans can be dissolved at higher temperatures, typically between 60 and 95 °C (Pereira et al. 2017). For this, two different methods are available: (i) refined carrageenan extraction and (ii) semi-refined extraction; both have the disadvantage of being considerably more expensive than the traditional but can obtain a polymer with a higher level of purity and yield.

A step-by-step practical guide of extraction and purification methodologies for the production of carrageenan (Table S9) and a comprehensive inventory of the required materials, reagents, and equipment can be found in the supplementary information.

#### Analytical Methods for Polymer Characterization

Likewise fucoidan, carrageenan exhibits several significant physicochemical properties that warrant evaluation for its diverse applications. Key factors include its molecular weight, which influences its gelling and thickening capabilities, crucial in food and pharmaceutical industries. The type and position of sulfate groups in carrageenan molecules determine its solubility, gel strength, and interaction with proteins, impacting product stability. The degree of esterification and the ratio of different carrageenan types influence its rheological behavior, aiding in controlling product textures. The ionic character, zeta potential, and salt sensitivity are essential for understanding carrageenan’s interactions in various formulations. Additionally, assessing its purity is vital for ensuring quality in food, cosmetics, and biotechnology applications. Some of these physico chemical characterizations are herein explained, (1) FTIR. In infrared spectroscopy analysis, the basic backbone of carrageenan contains a broadband approximately at 1240 cm^−1^ that matches to the stretching vibration of the S = O esters groups, with the band intensity/expression directly related to the contents of sulfate groups, which is characteristic of sulfated polysaccharides (Prado-Fernández et al. 2003). The signal around 930 cm^−1^ is related to the vibrations of the 3,6-anhydrogalactose bridges, prevalent in kappa-, iota-, and theta-carrageenan. The following two significant bands observed at 845 cm^−1^ and 805 cm^−1^ can reveal the identity of some carrageenan types. For this, the absorption band at 845 cm^−1^ is associated with the vibrations of C_(4)_-O-SO_3_, a fragment of the sulfated galactose. Characteristically, this band appears in kappa-, mu-, iota-, and nu-carrageenan spectra, while the band at 805 cm^−1^ can be observed only in iota-, and theta-carrageenan spectra. This second peak is associated with the vibrations of C_(2)_-O-SO_3_, a fragment of sulfated 3,4-anhydrogalactose (Gómez-Ordóñez and Rupérez 2011). Additionally, when hybrid carrageenans are obtained, it is possible to determine the iota/kappa carrageenans ratio using the relative intensity values of both peaks (805/845 cm^−1^ ratio) (Hilliou et al. 2006). (2) RAMAN spectroscopy is a non-destructive chemical analysis that can provide helpful and detailed information, regarding chemical structure, crystallinity, and molecular interactions. Comparatively, FTIR and RAMAN spectroscopies are complementary techniques, as both are based on molecular vibrations. In general, the RAMAN technique focuses on the scattering of radiation and depends on a change in polarizability of a molecule, while the FTIR uses the absorption of radiation and depends on a change in dipole moment (Geraldes 2020). In terms of analysis, the FTIR is more sensitive to hetero-nuclear functional group vibrations and polar bonds, especially O–H stretching in the water. On the other hand, the RAMAN is more sensitive to homo-nuclear molecular bonds, i.e., it can distinguish between C–C, C = C, and C≡C bonds (Muthuselvi et al. 2018). Furthermore, the RAMAN can provide better resolution than the FTIR spectra in carrageenan analysis due to its ability to identify different carrageenans. For example, some variants of the family of lambda-carrageenan are easily distinguished, such as the xi- and theta-carrageenan (Pereira et al. 2017). (3) Thin-layer chromatography (TLC) is a type of chromatography that can be used to separate non-volatile mixtures. This technique involves immersing an appropriate membrane (stationary phase) containing the samples in a flask with a solvent mixture (known as the mobile phase). Along the time, the different components of the sample are separated via capillary action. Compared with other techniques, the TLC offers some advantages to separate components due to relatively simple preparation, faster results, and comparatively inexpensive separation of different carrageenan types (Cheong et al. 2018). (4) High-performance liquid chromatography (HPLC) is a method that can be applied to separate, identify, and quantify components present in some mixture. This technique consists of passing the sample mixture through a column filled with a sold adsorbent material, and each component interacts slightly differently along the column, resulting in different flow rates for each element and subsequently causing a separation due to the different times that the components take to flow out of the column. (5) High-performance anion-exchange chromatography with pulsed amperometric detection (HPAEC-PAD) is a highly sensitive chromatography with good resolution for detecting monosaccharides, glycans, and oligosaccharides. Under strong alkaline conditions, the anions of oligosaccharides become weaker and can be well retained and separated using the anion-exchange column of the HPAEC-PAD (Yan et al. 2017). Unfortunately, this technique might not be suitable for routine analysis, as it requires a specific instrument compared with the TLC, and the detector performance depends on the condition of the PAD electrode, in which the response decreases with the number of injections (Jorge and Abdul-Wajid 1995). (6) Mass spectroscopy (MS) is a sensitive and powerful analytical technique used to quantify and identify known and unknown compounds and elucidate different biomolecules’ structure and chemical properties, such as oligosaccharides (Cheong et al. 2018). The process involves the conversion of the sample into gaseous ions, with or without fragments, which are characterized according to their specific mass-to-charge ratio (m/z) and relative abundance. For the carrageenan sample, this technique can obtain details about the accurate molecular weight, chain length distribution, fragments information, monosaccharide composition, and linkages and locate possible structural modifications (Lang et al. 2014; Kailemia et al. 2014). (7) Nuclear magnetic resonance. The chemical structure of carrageenans can be addressed by the potential proton of ^1^H and ^13^C-NMR spectroscopy. For this, the carrageenan sample can be dissolved using deuterated water (D_2_O). Due to the low natural abundance of the ^13^C isotope, the samples for this analysis should be prepared at relatively high concentrations (5–10% w/w) compared to ^1^H-NMR analysis (0.5–1.0% w/w). Moreover, the NMR analysis for carrageenans should be carried out at elevated temperatures to reduce the viscosity in the solution, being easier to manipulate for NMR tubes. The high viscosity is associated with obtaining a line broadening (Van de Velde et al. 2002). Therefore, this technique is extremely useful for carrageenan samples to identify the different types of carrageenan present in the sample and the purity.

### Ulvan

#### Sources, Characteristics, and Biological Properties

Seaweeds represent a rich but still underexploited source of bioactive compounds (de Freitas et al. 2015). The green sea lettuce, *Ulva* sp., is one of the most studied macroalgae (Jiménez-Escrig et al. 2011). It is an abundant genus of the seaweed group Chlorophyta, currently used for bioethanol fabrication and renewable gas fuel. In fact, it generally contains a small quantity of cellulose from which biogas is generated by anaerobic digestion (Vaishnavi et al. 2020). Typically, the cell wall has two combinations of compounds: (i) soluble ulvan and insoluble cellulose, and (ii) linear alkali-soluble xyloglucan and glucuronan (Madany et al. 2021). Ulvan provides 8% to 30% of the algae’s dry weight. This sulfated polysaccharide has been gaining attention for various industrial applications in the agriculture, food, pharmaceutical, chemical, and biomaterial industries (Cindana Mo’o et al. 2020). Ulvans are water-soluble sulfated heteropolysaccharides reported biological activities such as anticoagulant, antiviral, antioxidant, anti-allergic, anticancer, anti-inflammatory, and antihyperlipidemic (Qi et al. 2006a, b, 2013; Qi and Sun 2015; Radhouani et al. 2014; Aguilar-Briseño et al. 2015).

In the last years, there has been a peak of progress, with the respective publications, concerning exploring ulvan. A lot of this work was performed on ulvan lyases, but new modifications and potential applications were also explored. For instance, polyelectrolyte complexes (PEC) of ulvan and chitosan were evaluated as matrices for biomimetic mineralization, offering a greener scaffold fabrication route toward developing resorbable tissue engineering materials (Dash et al. 2018). Recently, it was possible to 3D (bio)print dermal-like structures, using methacrylate ulvan, biocompatible and biofunctional with enhanced mechanical, structural, and stability characteristics, for skin tissue repair (Chen et al. 2021). Furthermore, Kikionis et al. (2021) confirmed the ulvan osteoinductive capacity, confirming its potential in developing biomedical scaffolds for bone tissue regeneration applications (Kikionis et al. 2021). Another exciting work concluded that ulvan polysaccharides might have chemopreventive consequences against breast carcinogenesis (Abd-Ellatef et al. 2017).

#### Overview of the Extraction Methodology

**Fig. 10:**
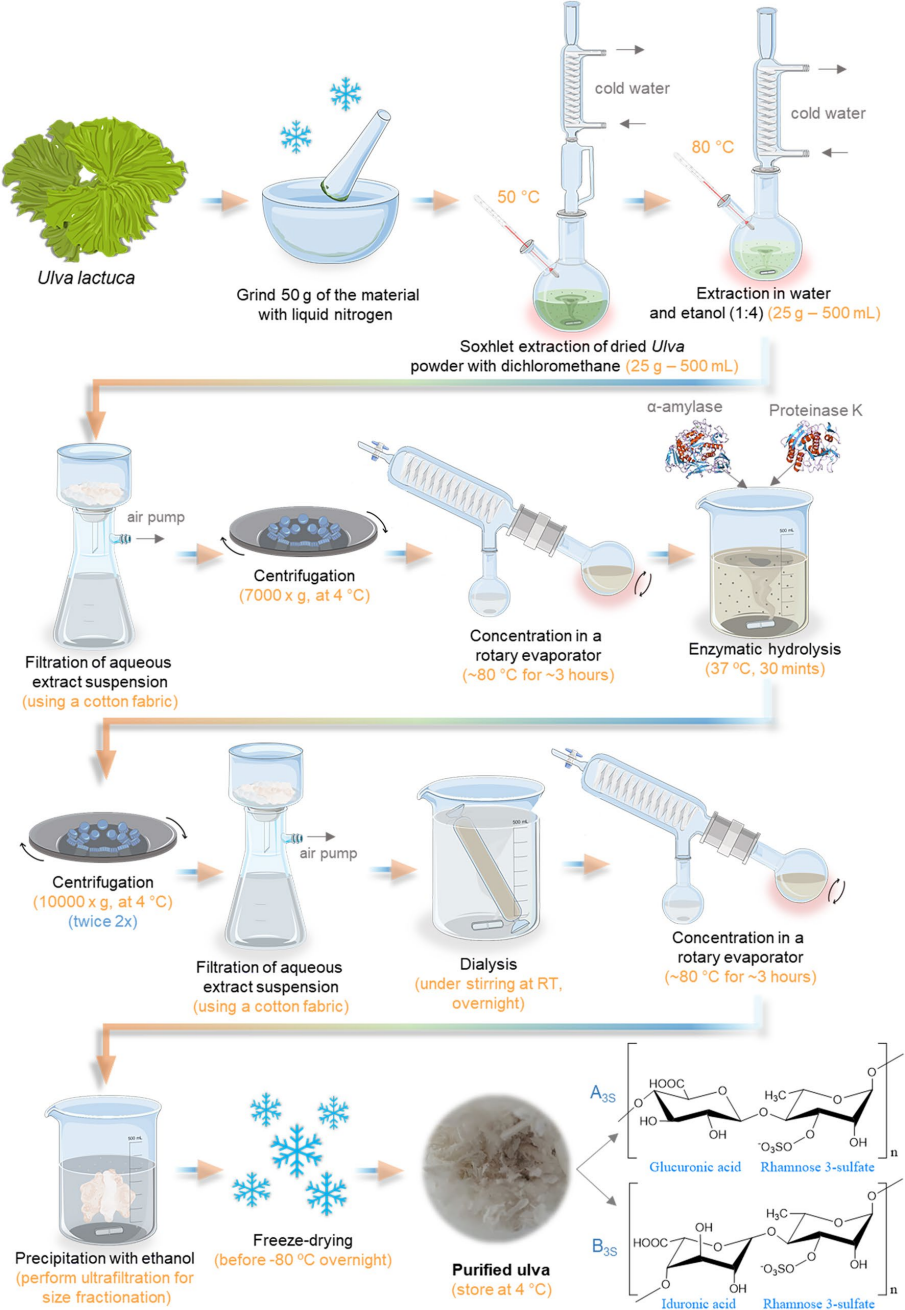
Schematic representation of Ulvan extraction methodology, illustrating the typical repeating unit structures of ulvan. (A_3S_) ulvanobiuronic acid type, composed of *β*-d-glucuronic acid and *α*-l-rhamnose-3-sulfate; and (B_3S_) is another ulvanobiuronic acid type, composed of *α*-l-iduronic acid and *α*-l-rhamnose-3-sulfate

The yield and the properties of ulvan change considerably with the extraction and purification processes, the source of the biomass, storage of collected biomass, and pre-extraction processing (Kidgell et al. 2019). Globally, the production of ulvan from green algae embodies four main stages; it starts with the (i) raw material recognition, selection, and collection; then the algae are (ii) cleaned, stabilized (if needed), and grinded; afterward, the most challenging stage involves the (iii) extraction and purification, followed by the ulvan (iv) precipitation, drying, and careful storage (Pinto 2012). In the first stage, the main precaution is cross-contamination by other organisms and determining appropriate habitat, geography, and seasonality. The second stage begins the laboratory procedures by performing the algae pre-treatment, performed by stabilization (hot-air drying, freezing, freeze-drying, or dry salting) (Robic et al. 2008). Finally, the algae are washed and dried (thermally), frozen, drying methods, brining, or dry salting; depending on the technique used, higher yields or higher molecular weight and viscosity can be obtained (Pinto 2012). The ulvan quantity and quality are highly affected by the extraction method and the selected solvent (Cindana Mo’o et al. 2020). Typically, the technique (Alves et al. 2010; Cindana Mo’o et al. 2020) comprises the following steps demonstrated in Fig. 10.

A step-by-step practical guide of extraction and purification methodologies for the production of ulvan (Table S10) and a comprehensive inventory of the required materials, reagents, and equipment can be found in the supplementary information.

#### Analytical Methods for Polymer Characterization

Ulvan possesses key physicochemical properties that are vital for its diverse applications. Evaluating ulvan’s molecular weight is essential as it impacts its solubility and bioactivity. Likewise, understanding the degree and pattern of sulfation is crucial for its anticoagulant and antiviral properties. Ulvan’s conformation, branching, and charge density significantly affect its interactions with other molecules, making it important to assess these aspects. The purity and composition of ulvan also need to be determined to ensure consistent quality in various industries, including food, pharmaceuticals, and environmental applications. Some of these physicochemical characterizations are herein explained. (1) Infrared spectroscopy. Ulvan structure spectrum shows a group of strong absorbance values (1650 cm^−1^, 1250 cm^−1^, and 1070 cm^−1^) and another of smaller ones (1400 cm^−1^, 850 cm^−1^, and 790 cm^−1^) (Robic et al. 2009; Ray and Lahaye 1995), namely those assigned to -COOH asymmetrical stretching (1650 cm^−1^), to -SO_3_—with = S = O stretching (1250 cm^−1^), to CH-O–CH asymmetrical stretching (1050 cm^−1^), the -COOH symmetric stretching (1400 cm^−1^), and also the ones related to sugar cycles and sulfation at axial and equatorial positions (800 and 850 cm^−1^). Moreover, it is also usually feasible to discern the following absorbance bands: 3400 cm^−1^ broadband (O–H stretching vibrations) and 2950 cm^−1^ (C-H bond) (de Freitas et al. 2015; Pereira et al. 2013). (1) NMR is among the characterization set of methods used for ulvan. Proton NMR is used (^1^H-NMR) routinely to validate the polysaccharide structure. The spectrum could be acquired at different temperatures. However, 60 °C is an excellent choice to move the deuterated water peak from the spectra' critical region of the anomeric peaks. However, most authors acquire them at 25 °C (Barros et al. 2013; Robic et al. 2008; Lahaye 1998). The anomeric proton signals are found at chemical shifts of 4.80 ppm to 4.95 ppm and 4.60 ppm to 4.67 ppm, and 5.29 ppm are from* α*-l-rhamnose (and *α*-d-rhamnose), *β*-glucuronic and* α*-xylose acids (Guidara et al. 2021). Excluding the anomeric peaks, most of the peaks from ring protons are concentrated in a single region from 3.23 to 4.54 ppm, except one that appears before, in the chemical shift around 1.32 ppm, relative to the C6 proton of rhamnose. (2) Elemental analysis is frequently used to determine the elemental content of an unknown substance. This kind of technique assumes immense importance for molecules extracted from natural products since it can be qualitative and quantitative analysis (Zhu et al. 2016). The sample is placed at the combustion reactor after weighing using an automatic sampler, together with a certain amount of oxygen. The final analysis generates a complete report with results of sample composition in C, H, N, and S in total percentage from 0.01% (100 ppm) to 100%. (3) Molecular weight by GPC Ulvan is well-known as an anionic polysaccharide, and thus, the column to be used should reflect this. Therefore, a column separating neutral and anionic polymers in aqueous eluents should be installed at the GPC apparatus, such as Suprema. The system should be kept at 30 °C and the eluent based in phosphate buffer solution (typically with sodium azide to prevent microorganisms’ growth) with 1 mL min^−1^ of flow rate. The calibration is performed with an individual molecular weight standard or an entire set of standards with different sizes. (4) High-performance liquid chromatography (HPLC) is a method used to separate, identify, and quantify components present in the sample. For example, this technique applied to ulvan samples can determine the molecular weight and the presence of different sugars as a measure of purity.

### Chondroitin Sulfate

#### Sources, Characteristics, and Biological Properties

**Fig. 11:**
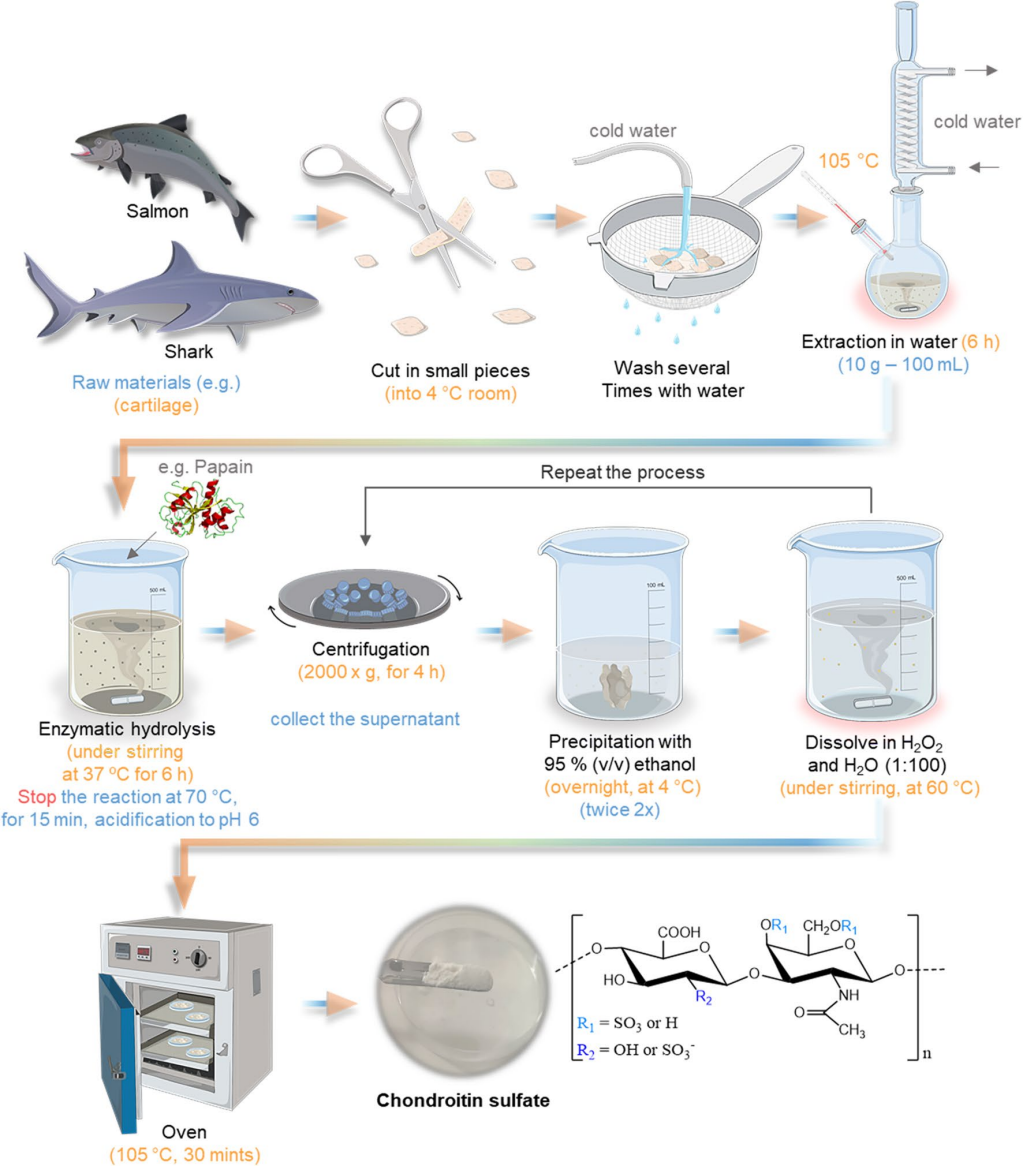
Schematic representation of chondroitin sulfate extraction methodology applied to fish cartilage, together with the general molecular structure of CS. R_1_ and R_2_ represent the variations in the terminal groups

Chondroitin sulfate is a linear polysaccharide formed by 20 to 100 repeating disaccharide units of (1–3)-*β*-*N*-acetyl-d-galactosamine and (1–4)-*β*-glucuronic acid (Poole et al. 2015; Vázquez et al. 2013; Abdallah et al. 2020b), linked together via *β*-(1 → 3) glycosidic linkages (Kumari and Badwaik 2019) (Fig. 11). The amount of repeating uronic acids depends on the source, even if the same tissue is used (Caballero et al. 2003). It could be sulfated at positions 4 or 6, deriving in the two main chondroitin sulfates, A and C. These sulfation patterns at positions 4 and 6 are determined by the activity of specific enzymes called sulfotransferases during its biosynthesis, namely C4 sulfotransferase (CHST11) and C6 sulfotransferase (CHST3) (Kumari and Badwaik 2019).

CS is one of the ECM components of several connective tissues, such as skin, bone, cartilage, ligaments, and tendons. It is recognized for the compressive strength of cartilage tissue since it can readily absorb water (Salinas et al. 2013). Still, besides the animal tissues that can be used as CS sources, it can also be obtained from certain bacteria (Kumari and Badwaik 2019), for instance, using *Pasteurella multocida* or *Escherichia coli* (Vázquez et al. 2013). Nevertheless, CS is obtained mostly from cartilage by-products from both mammalian and fishery origins. CS production from marine sources relies on several sources: shark cartilage, ray cartilage, fin, skate cartilage and fin, zebrafish cartilage, dogfish cartilage, and salmon nasal cartilage (Vázquez et al. 2013). CS derived from cartilaginous fish (mainly ray and shark) is referred to as a better source than mammalian because of its sulfation pattern and safety (Abdallah et al. 2020b).

Chondroitin sulfate has good stability, minimal toxicity, chemical modification possibilities, and specific biodegradability (Babu et al. 2019). It is implicated in several biochemical activities like antioxidants, anticoagulation, anti-inflammation, and antiapoptotic (Kumari and Badwaik 2019). The main application of this water-soluble polymer is for joint disorders (such as osteoarthritis—OA), commonly recognized as a supplement in OA management (Barrow and Shahidi 2007). Moreover, another role as a pharmacological agent may be achieved via direct uptake or part of a drug delivery system (Babu et al. 2019). Besides, it has been used with materials, such as collagen, to formulate scaffold matrices due to their capacity to absorb large amounts of water and maintain a hydrated environment, combined for be a biologically active matrix that allows cell survival and their normal functionalities such as cell adhesion, proliferation, or even differentiation (Kumbar et al. 2014). In fact, CS is also able to bind to a range of growth factors and cytokines, which can help to regulate cell behavior and promote tissue regeneration, such as bone and cartilage.

#### Overview of the Extraction Methodology

The marine by-products are a potential source for extracting valuable compounds, such as CS, which is both an environmentally and economically practicable approach. The methods of chondroitin sulfate isolation from cartilage have been defined for several years. Usually, they are built on the chemical hydrolysis of the tissue for proteoglycan core disruption, and then the elimination of proteins is performed to recover the product. Usually, the process starts by gathering the raw material, for instance, the cartilage of codfish, squid, catshark, salmon, octopus, spiny dogfish, sturgeon, and tuna, preferably from cartilaginous fish such as shark, skate, or ray (Abdallah et al. 2020b). For instance, using blue shark (*Prionace glauca*) cartilage, the process is demonstrated in Fig. 11.

A step-by-step practical guide of extraction and purification methodologies for the production of chondroitin sulfate (Table S11) and a comprehensive inventory of the required materials, reagents, and equipment can be found in the supplementary information.

#### Analytical Methods for Polymer Characterization

Chondroitin sulfate, a glycosaminoglycan found in connective tissues and often used in dietary supplements and pharmaceuticals, possesses several key physicochemical properties that demand assessment. These include its molecular weight, which influences its absorption and bioavailability. Evaluating the degree of sulfation is crucial, as it impacts chondroitin sulfate's anti-inflammatory and joint health effects. The structural characteristics, such as the pattern of sulfation and the arrangement of sugar units, play a role in its functionality. The purity of chondroitin sulfate and the presence of impurities must be determined to ensure product quality. Furthermore, understanding its solubility, charge, and interactions with other substances is important for various formulations and applications. Some of these physicochemical characterizations are herein explained. (1) High-performance liquid chromatography. HPLC is an effective method for separating, identifying, and quantifying the components of mixtures with extremely high precision (Robens et al. 1999). Chondroitin sulfate can be analyzed, for instance, using an Atlantis® dC18 column (silica-based, reversed-phase) at 25 °C eluted by mobile phase CH_3_CN and 1% phosphate buffer (10:90, v/v) at a flow rate of 0.6 mL/min. The detection wavelength was set at 195 nm. The retention time for CS is approximately 2.99 min (Xie et al. 2014). However, this retention time can vary according to the column used, the eluent, the flow rate, or even the molecular weight of the polymer. (2) Infrared spectroscopy. Typically, a characteristic CS structure spectrum has strong absorbance bands at 1627–1637 cm^−1^ and approximately 1420 cm^−1^, implying the presence of carboxyl, amine, and sulfate groups (Sundaresan et al. 2018). Furthermore, the strong absorptions at 1650 cm^−1^ (stretching vibration of the carbonyl bond of the amide group) and 1556 cm^−1^ (bending vibration of the N–H bond) demonstrate the existence of the acetamido group. (3) SDS-PAGE could be applied to determine the molecular weight of CS qualitatively. A 12% stacking gel and a 5% resolving gel with a constant current of 50 milliamperes (mA) are needed to perform electrophoresis. For this, the samples are loaded in each well, and the molecular weight ladder markers are used to assess the bands molecular weight. Then, gels are stained with 0.1% (w/v) Coomassie Brilliant Blue in 50% (v/v) methanol and 6.8% (v/v) acetic acid and destained with 7.5% (v/v) acetic acid and 5% (v/v) methanol. The obtained molecular weights depend highly on the CS source and used method. (4) Dimethylmethylene blue (DMMB) assay could be applied for quantifying GAG. The method is built on the metachromasia phenomenon, in which the characteristic blue of the cationic DMMB dye shifts to a violet hue with the dye binding to polyanionic substrates (Zheng and Levenston 2015). For this, the absorbance of the standard curve and the samples should be recorded at 525 nm using a spectrophotometer. (5) NMR analysis is a powerful technique that can be performed to obtain ^1^H and ^13^C spectra of CS solutions previously prepared with D_2_O, primarily to access the sulfated pattern (Mucci et al. 2000). In ^1^H NMR, the most relevant patterns appear at around 4.5–5 ppm, associated with anomeric protons attached to the glycosidic linkages between the hexosamine and uronic acid residues. The second relevant peak is associated with sulfate protons that appear as singlets at around 2.0–2.5 ppm, and the protons on the *N*-acetyl groups and the methyl group of the O-sulfate group occur at approximately 1.9–2.2 ppm, and 3.5, respectively. On the other hand, on ^13^C-NMR the most important peaks are related to carbon atoms in urinic acid residues (170–180 ppm), in the hexosamine residues (55–75 ppm), in the *N*-acetyl group (25–35 ppm), and in the sulfate group that typically appear in the range of 60–80 ppm.

### Hyaluronic Acid

#### Sources, Characteristics, and Biological Properties

Hyaluronic acid or hyaluronan (HA), by definition, is a linear glycosaminoglycan with high molecular weight consisting in the regular repetition of non-sulfated disaccharide units of d-glucuronic acid and N-acetyl-d-glucosamine linked by glycosidic bonds (Giji and Arumugam 2014; Abdallah et al. 2020a; Liu et al. 2011). It is an anionic biopolymer largely distributed in the connective tissues, the major macromolecular component of the extracellular matrix. HA can be found widely in prokaryotic to eukaryotic cells, especially in the cell wall of Streptococci bacteria, vitreous humor (VH) of the eye, skin, umbilical cord, synovial fluid, and rooster comb (Papakonstantinou et al. 2012). Due to its capacity to absorb water molecules and swelling properties, HA plays an essential role in tissue permeation and hydration, transport of macromolecules between cells, and bacterial invasiveness (Vazquez et al. 2013). In addition, this biopolymer features and chemical structure provides a wide range of physicochemical and biological properties and functions such as lubricity, viscoelasticity, biocompatibility, angiogenic, and immunostimulatory (Liu et al. 2011; Gupta et al. 2019). HA has become a well-discussed component in different areas, such as biotechnology, cosmetics, biomedical, bioengineering, and pharmaceutical, with many boasting its different uses, benefits, and high economic importance. Its natural function of supporting collagen fibrils makes hyaluronic acid ideal for developing materials for wound and arthritis treatment, as tissue scaffolds, drug delivery, and as components of implantable devices (Ivanova et al. 2014). Moreover, the activity of HA, its therapeutic effect, and its specific usage area depend directly on the molecular weight that depends on the source (Horkay et al. 2009; Snetkov et al. 2020).

The traditional sources of HA regarding industrial production are rooster combs and diverse mammalian sections, besides bacterial fermentation, which represents the most used and efficient process. However, marine resources, especially fish by-products and mollusc bivalves, have also been explored as an alternative source of HA, safeguarding the maximum exploitation of marine by-products (Murado et al. 2012; Silva et al. 2012b; Giji and Arumugam 2014).

#### Overview of the Extraction Methodology

**Fig. 12:**
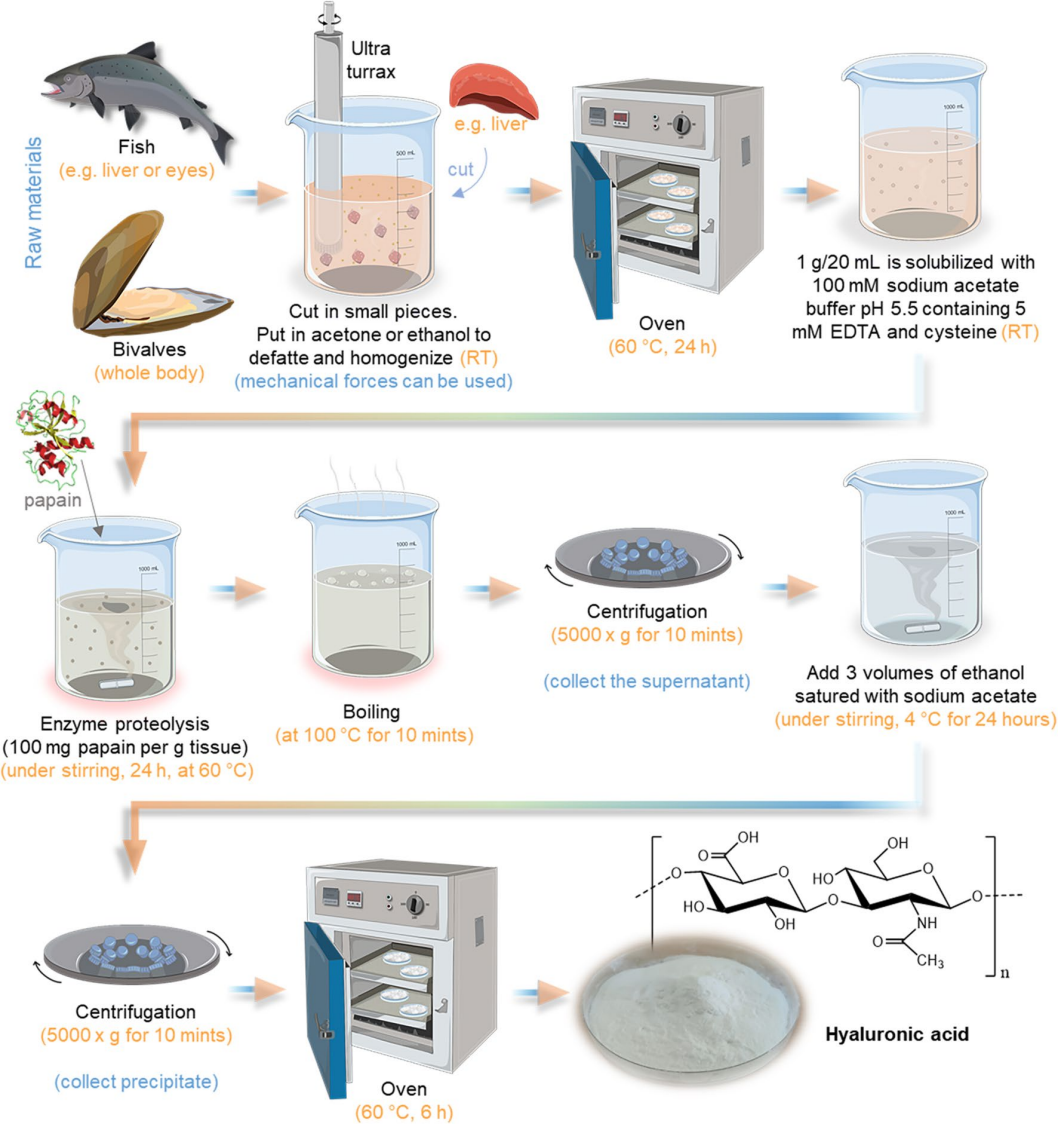
Schematic representation of hyaluronic acid extraction methodology, together with its chemical structure

To break down the cellular tissue structures and isolate HA from other polysaccharide complexes, various techniques were used based on detergents, enzymes, and/or solvents (Abdallah et al. 2020a; Vazquez et al. 2013). The most frequently used enzyme digestion method to extract HA from fish by-products, such as the liver of stingrays (Sadhasivam et al. 2013) and mollusc bivalves (Volpi and Maccari 2003), is based on papain digestion. In general, as shown in Fig. 12, this process consists of the first degreasing phase with acetone, followed by enzyme digestion, a boiling step to denature the enzyme, and precipitation using ethanol saturated with sodium acetate (Abdallah et al. 2020a; Sadhasivam et al. 2013). The entire extraction process is demonstrated in Fig. 12.

A purification step can be required to certify the higher purity of HA (Abdallah et al. 2020a), which is typically based on size separation using ultrafiltration-diafiltration systems (Choi et al. 2014; Zhou et al. 2006) or even using chromatography (Sadhasivam et al. 2013). The isolation of HA from the VH from fish eyeballs can also be achieved by using an optimized extraction and purification process described and developed by Murado et al. (2012), which comprises the recovery of VH from frozen eyes using nylon meshes. Then, a clarification based on electrodeposition combined with an ultrafiltration-diafiltration system is performed, followed by a selective recovery of the precipitate (alcoholic precipitation), an alkaline treatment in a hydroalcoholic medium with controlled conditions, and a recovery of HA by dissolving the precipitate in hydroalcoholic phosphate monosodium. Although bacterial production of HA has already been developed and studied industrially, no marine microorganisms have been found so far capable to produce this biopolymer. The only possible approach to this biofabrication is the use of a cultivation medium formulated with nutrients generated from marine biomass (Vázquez et al. 2009, 2010). A step-by-step practical guide of extraction and purification methodologies for the production of hyaluronic acid (Table S12) and a comprehensive inventory of the required materials, reagents, and equipment can be found in the supplementary information.

#### Analytical Methods for Polymer Characterization

Hyaluronic acid, a naturally occurring glycosaminoglycan with numerous medical and cosmetic applications, exhibits several crucial physicochemical properties that demand evaluation. These include its molecular weight, which affects its viscosity and performance in different formulations. Assessing the degree of cross-linking is also vital, since it influences its durability and residence time in tissues for applications like dermal fillers. Understanding the purity and potential impurities is essential to ensure product safety and quality. Hyaluronic acid’s water-binding capacity and its ability to retain moisture are key for its hydrating properties in skincare and wound healing. Additionally, evaluating its rheological behavior, such as viscoelasticity, aids in tailoring its application-specific properties. Some of these physicochemical characterizations are herein explained. (1) Electrophoretic analysis is critically important in HA evaluation because it allows the determination of the molecular weight distribution of the polysaccharides complex, but also the purity degree of the extract (Giji and Arumugam 2014). Different types of electromigration can be used to analyze HA, being more commonly explored the agarose gel electrophoresis (Lee and Cowman 1994; Sadhasivam et al. 2013). (2) FTIR enables the study of the fundamental absorption frequencies, which facilitates understanding the structure-spectral relationship of the associated molecular vibrations. This analysis should identify the presence of hydroxyl group -OH stretch and N–H stretch (3400–3200 cm^−1^), methylene C-H asymmetric/symmetric stretch (2850–2930 cm^−1^), CH stretching (3000–2800 cm^−1^), C = O carboxyl amide I (1650–1630 cm^−1^), CH_2_ (1460–1350 cm^−1^), CH_3_ (1370–1350 cm^−1^), C-O–H deformation (1310–1240 cm^−1^), C-O with C-O combination (1170–1030 cm^−1^), primary aromatic amine CN stretch (1280–1240 cm^−1^), C–O–C (1150–1000 cm^−1^), C-O (1050–1000 cm^−1^), and C-O–H stretching (3500–3200 cm^−1^) (Manju and Sreenivasan 2011). (3) NMR analysis is a powerful technique to provide information on the hydrogen atom position of HA solutions prepared with D_2_O. It also determines the presence of glucuronic acid and *N*-acetyl glucosamine H-1*β* glycosidic linkages, thus contributing to the identification of this biopolymer (Sadhasivam et al. 2013; Giji and Arumugam 2014). (4) HA content quantification is essential to evaluate the extracts’ purity, generally based on uronic acid content. However, it can also be verified through methods such as HPLC that determine and quantify the oligosaccharide products or even by ELSA (ELISA-like assays) based on enzyme-linked sorbent assays (Cowman et al. 2015a). (5) GPC is the routine method used to measure HA's molecular weight and structural characteristics. For this analysis, HA can be dissolved (1 mg mL^−1^) in PBS buffered Saline (0.01 M phosphate buffer, 0.0028 M potassium chloride, and 0.138 M sodium chloride, pH 7.4 at 25 °C, Sigma-Aldrich) and 0.05% w/v NaN_3_, and can be measured, for example, on SUPREMA column (PSS—Polymer Standards Service, DE) due to alginate be an anionic polymer (Sadhasivam et al. 2013). (6) Rheological analysis is important to evaluate the viscoelastic properties of HA that are directly correlated with its molecular weight. Moreover, HA solutions can be characterized as non-Newtonian fluids with shear-thinning and viscoelastic behavior (Kim et al. 2018; Cowman et al. 2015b; Snetkov et al. 2020).

### Alginate

#### Sources, Characteristics, and Biological Properties

**Fig. 13:**
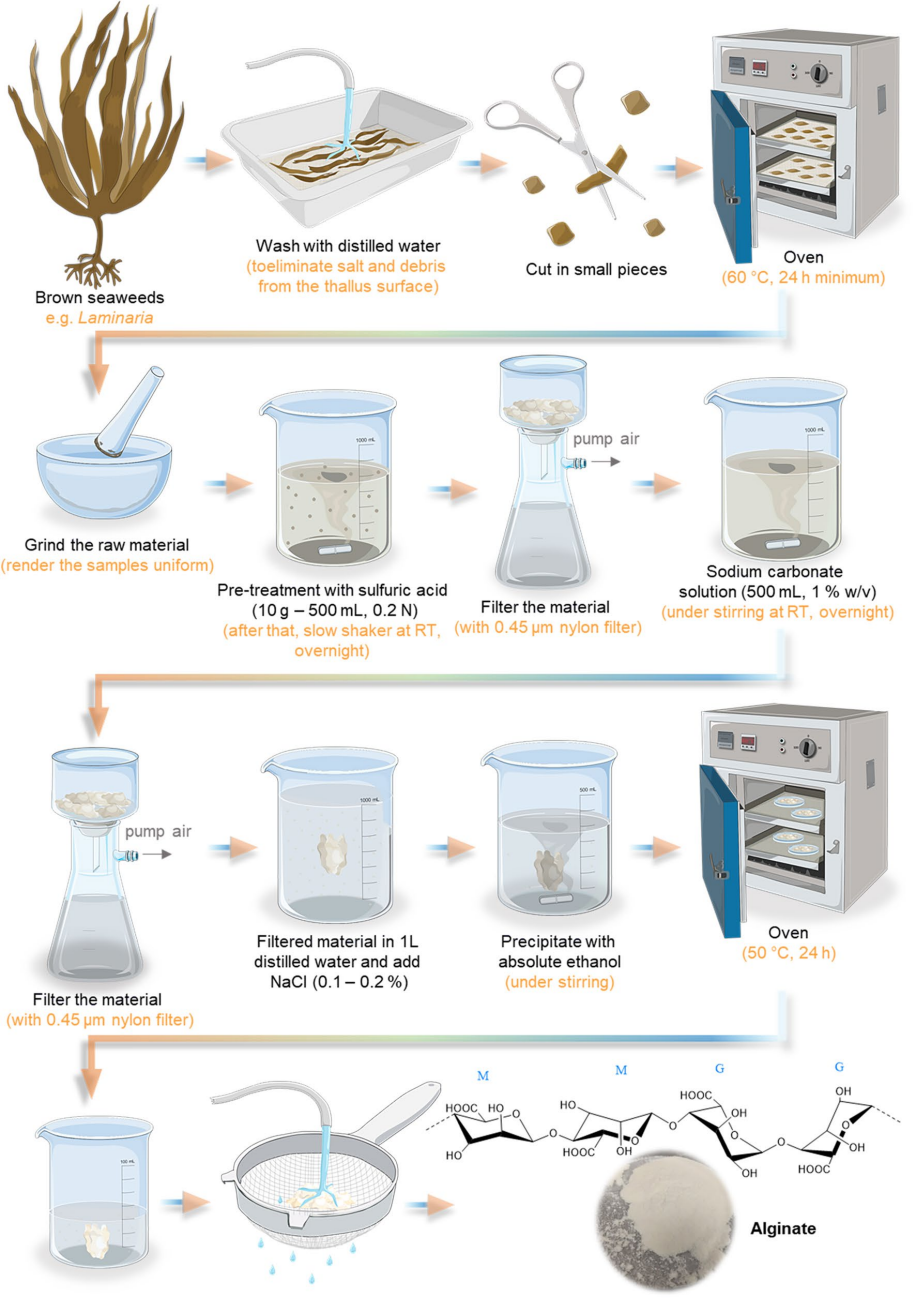
Schematic representation of alginate extraction methodology being presented the M-block (*β*-d-mannuronic acid) and the G-block (*α*-l-mannuronic acid) composing the chemical structure of this biopolymer

Alginate is a generic term for alginic acid salts, which is an anionic and water-soluble (Jahandideh et al. 2021) natural polymer that is mainly found in several brown algae species, including from the genera *Laminaria*, *Macrocystis*, *Durvillaea*, *Ecklonia*, *Undaria*, *Lessonia*, *Macrocystis*, *Sargassum*, *Turbinaria*, and *Ascophyllum* (Angra et al. 2021; Alihosseini 2016), but not land plants (Sudha 2017). Commercially, the most available alginate is from *Laminaria digitata*, *Laminaria hyperborean*, and *Macrocystis pyrifera*. However, it is also possible to obtain alginate through microbial production approaches, using several types of bacteria, such as *Azotobacter vinelandii* and *Pseudomonas aeruginosa* (Singha et al. 2021). Structurally, alginate is a polyuronidic acid because these polysaccharide molecules are assembled with uronic acid residues (Singha et al. 2021). It is essentially composed of two monomers, the (1,4)-linked *β*-d-mannuronic acid (M-block) and *α*-l-guluronic acid (G-block) (Fig. 13), organized by two homogeneous and one heterogeneous patterns. The homogenous is the diequatorial (MM-blocks or also called poly-M) and by diaxial (GG-blocks or poly-G), while the heterogeneous is namely equatorial-axial or also called MG-block (Membere and Sallis 2018; Abasalizadeh et al. 2020). The MG contents will vary with the source, location, and seasonality (among other factors), which influences the properties of alginates (Jahandideh et al. 2021; Angra et al. 2021; Alihosseini 2016). It is known that the G-blocks directly affect the properties of the alginate. When it contains a higher content and length of G-block and sequentially the molecular weight, it is responsible for forming a stronger gel (greatly viscous) (Lee and Mooney 2012). In fact, due to the G-block composed of guluronic acid residues, they have a higher affinity for calcium ions than mannuronic acid residues. When subjected to divalent cations, such as calcium ions, the G-block forms a gel through ionic crosslinking between the polymer chains, contributing to higher gel strength and rigidity. On the other hand, a high presence of M-block contents is responsible for the presence of biological properties such as immunogenic reactions (Pina et al. 2015). Recently, specific alginate has been described with immune-compatible performance, offering new opportunities for the biomedical use of this biopolymer.

In the last decade, alginates have revealed interesting properties that can be applied to several industrial sectors such as food, textile, and pharmaceutical or also for medical applications due to their rheological and mechanical properties, but also due to possible antioxidant and anti-inflammatory properties, low toxicity, and good biocompatibility (Christy et al. 2021). For instance, in fiber form, alginate can be applied to several products, such as woven, nonwoven, and knitted fabrics (Jahandideh et al. 2021). At the same time, for (bio)medical applications, it could be used for skin grafting in the plastic surgical procedure (Christy et al. 2021), wound dressings (Aderibigbe and Buyana 2018), drug delivery (Tonnesen and Karisen 2002), dental printing materials (Singha et al. 2021), and tissue engineering due to their potential to form gels spontaneously due to its intrinsic hydrophilicity and ionic crosslinking with divalent cations (such as Ca^2+^ and Mg^2+^) (Tariverdian et al. 2019). Furthermore, alginate is used as a stabilizer, thickener, gelling, emulator, and film-forming component in the food and pharmaceutical sectors due to its high stability and rheological performance (Singha et al. 2021; Christy et al. 2021).

#### Overview of the Extraction Methodology

Alginates could be obtained from brown algae or bacterial sources. However, the leading environmental and economic approach is considered to be the use of kelp. In fact, alginic acid and its salts (Ca, Mg, Na, and K) are around 40% of the brown algae’s dry weight (Additives et al. 2017). Note that alginate is the terminology generally applied to alginic acid salts. Besides, it can effortlessly co-extract alginate using fucoidan extraction (Ale and Meyer 2013). The process typically requires mild acid treatments (HCl or CaCl_2_) to the washed and milled dried algae be cleaned from unwanted compounds (counter ions and impurities) while converting cell wall alginate into alginic acid. Then, insoluble alginic acid is collected as soluble sodium salt after neutralization (NaOH or Na_2_CO_3_), and insoluble residues are removed (by filtration, flotation, or centrifugation). Finally, the soluble alginate is precipitated (Sudha 2017), using different techniques. Thus, the process has three main stages, (i) pre-extraction, (ii) neutralization, and (iii) purification/precipitation; residing in this last step the most significant differences between methods. Therefore, the method for extraction and purification could be as follows (Sudha 2017). The detailed procedure is demonstrated below and in Fig. 13.

A step-by-step practical guide of extraction and purification methodologies for the production of alginate (Table S13) and a comprehensive inventory of the required materials, reagents, and equipment can be found in the supplementary information.

#### Analytical Methods for Polymer Characterization

Alginate possesses several key physicochemical properties that necessitate assessment for its diverse applications. These include its molecular weight, which influences its viscosity and gel-forming capabilities, essential for applications in the food and pharmaceutical industries. The degree of guluronic and mannuronic acid residues, as well as the block structure of alginate, plays a significant role in its gelation properties. Additionally, understanding its solubility and interactions with ions is crucial for applications such as drug delivery and wound dressings. The purity and impurity levels of alginate need to be determined to ensure consistent quality in various formulations. Moreover, the rheological behavior and viscosity of alginate are important for controlling its texture in food products and medical uses. For this, it is explained some of these physico chemical characterization: (1) FTIR. The characteristic alginate structure spectrum is somewhat dependent on the alginate type. In fact, besides being very similar, the spectrum of each type has its characteristic fingerprint at a wavenumber between 950 and 750 cm^−1^, relative to C-O stretching of the uronic acid residue (Yudiati and Isnansetyo 2017). For instance, calcium alginate at 933.5 cm^−1^, sodium alginate at 949.0 cm^−1^ and 895.0 cm^−1^, and acid alginate at 941.3 cm^−1^ and 887.3 cm^−1^ (Yudiati and Isnansetyo 2017). The remaining spectrum, for instance, for sodium alginate, has a wide band at 3428 cm^−1^ attributed to O–H stretching vibrations and two more at 2929 cm^−1^ and 1608 cm^−1^ assigned to C–H stretching and O–C–O asymmetric stretching vibrations, respectively. The absorption band at 1415 cm^−1^ is assigned to C–OH deformation vibration. The bands located at 1315 cm^−1^, 1090 cm^−1^, and 1033 cm^−1^ are related to the deformation of C–C–H (and O–C–H), C–O stretching vibrations, and C–O (and C–C) stretching vibrations of pyranose rings, respectively (Fenoradosoa et al. 2010). Additionally, it is possible to characterize the block structure type on this technique, in which the M component can be observed at around 1.177 cm^−1^ being associated with C–O–C stretching vibration, and the G component at around 1.418 cm^−1^. (2) NMR is one of the characterization techniques used for alginates and is a reliable method for characterizing the block structure of alginate composition. The three key peaks ascribed to alginates are located in the anomeric region. Namely, the anomeric hydrogen of guluronic acid at 5.1 ppm to 5.2 ppm, mannuronic acid and the H-5 of alternating blocks overlapping at 4.7 ppm to 4.9 ppm, and the H-5 of glucuronic acid residues at 4.5–4.6 ppm (Belattmania et al. 2020). Moreover, the remaining H-2 to H-6 atoms appear with peaks at 3.74 to 3.98 ppm, while the O-(C = O)CH3 between 2.06 and 2.51 ppm, and the CH_3_ group appears with a chemical shift between 1.7 and 1.8 ppm (Belattmania et al. 2020). (3) GPC is the routine method that can be used to measure alginate’s molecular weight and structural characteristics. For this analysis, alginate can be dissolved (1 mg mL^−1^) on the eluent with 0.15 M ammonium acetate (NH_4_OAc) and 0.2 M acetic acid (AcOH) solution (pH 4.5) and be measured, for example, on SUPREMA column (PSS—Polymer Standards Service, DE) due to the alginate be an anionic polymer (Feng et al. 2017). (4) Thermogravimetric analysis (TGA) is a valuable technique to determine alginate’s thermal behavior and identify the presence of eventual impurities. As usual, alginate typically shows a gradual decline with rising temperatures, composed of four decomposition phases. The first stage is characteristically attributed to the initial dehydration process (temperature range between 50 and 70 °C), then two decomposition phases are related to carbonaceous residue (245–300 °C and 530–570 °C). Finally, the last mass loss is associated with the production of sodium carbonate (640–660 °C) (Belattmania et al. 2020). (5) Rheological analysis is essential to evaluate the alginate jellying behavior and its viscoelastic properties that correlate directly with the molecular weight. Moreover, it is particularly important to consider rheological studies such as oscillatory as a function of the frequency, oscillatory as a function of the range of temperature, and viscosity to predict and optimize alginate behavior for its later use, for example, for the manufacturing of scaffolds like 3D printing structures (Rezende et al. 2009). Alginate is generally characterized as non-Newtonian fluids with shear-thinning and higher viscoelastic behavior.

### Agar and Agarose

#### Sources, Characteristics, and Biological Properties

**Fig. 14:**
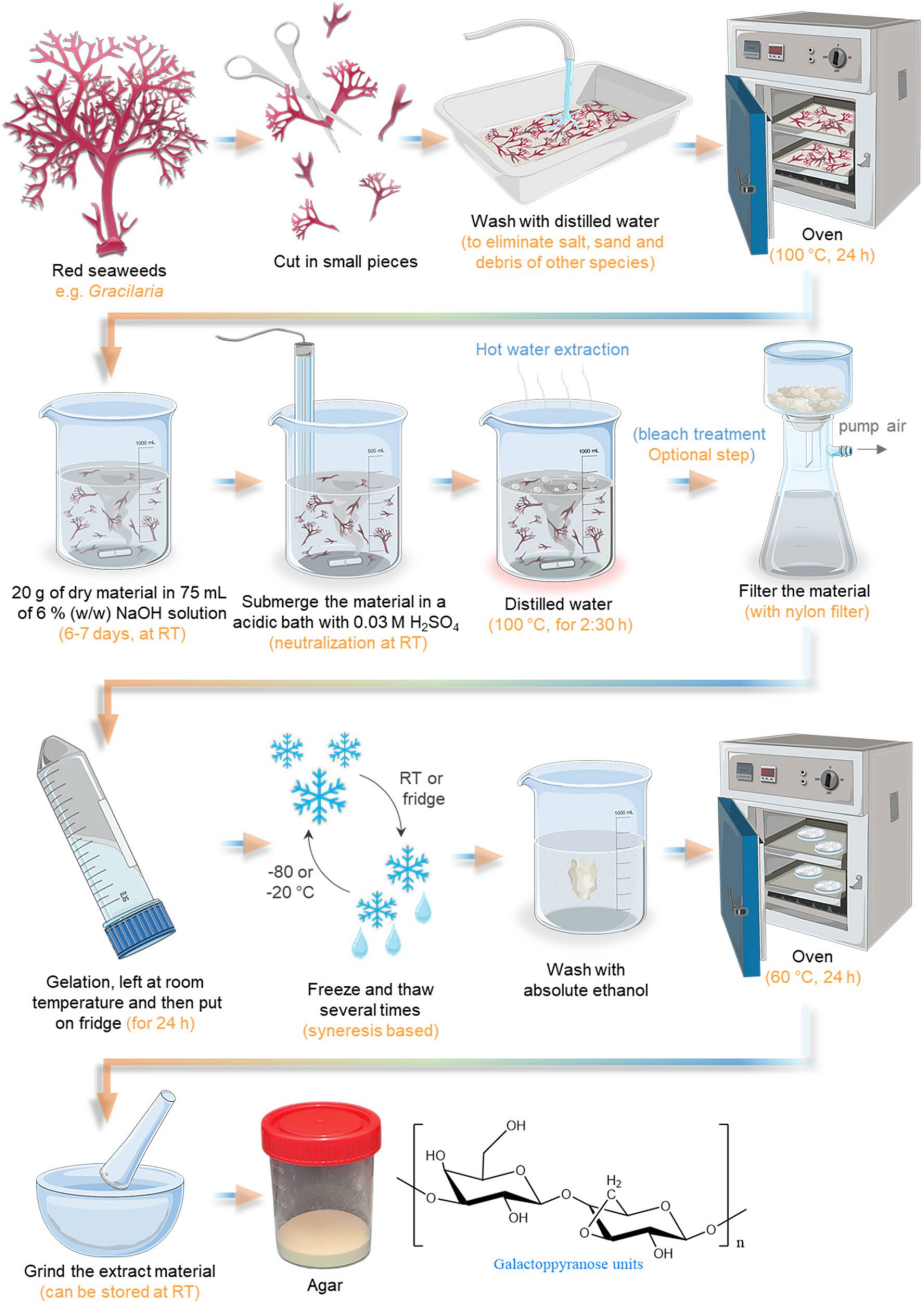
Schematic representation of agar extraction methodology, being also represented the chemical structure of agar exhibiting the galactopyranose units

Agar is a mixture of polysaccharides found in the cellular walls of red algae, namely agarophytes belonging to the class Rhodophyta (Lebbar et al. 2018). Their abundance is particularly relevant in members belonging to *Gelidiaceae* and *Gracilariaceae* families (Marinho-Soriano 2001). Currently, agar has strong economic importance due to its ability to form a gel at low concentrations and become solid at room temperature. In this order, it has a huge interest, for example, in its use as a bacteriological medium. Nowadays, agar formulations are among the most explored biopolymers of marine origin, with high economic interests in the industry due to their several applications. For instance, aimed at the food industry, agar can be used as an appetite suppressant, a vegetarian substitute for gelatin from mammal sources, a thickener for soups, fruit preserves, and some desserts as ice cream, as well as a clarifying agent in the brewing process (Jaswir et al. 2016). Furthermore, in laboratory applications, it is commonly used in salt bridges for electrochemistry or for microbiology to feed and provide an adequate environment (i.e., nutrients and support) for the growth of bacteria and microorganisms (Franco-Duarte et al. 2019). To overcome the higher demand for this polysaccharide, commercial agar is abundantly extracted from diverse marine algae genera, like *Gelidium*, *Gracilaria*, *Ceramium*, *Acantkopeltis*, and *Pterocladia* (Villanueva et al. 2010) due to their high levels of agar contents and to the capacity of some species to adapt in aquaculture conditions (Li et al. 2008). To simplify the general structural composition, agar is fundamentally composed of neutral agarose and charged agaropectin (non-gelling fraction) (Sasuga et al. 2017). However, a correct approach to its structure is considered agar as a complex mixture of water-soluble galactan derivatives made up of linear and alternating (1 → 3) and (1 → 4) linkages (Silva et al. 2012b). Structurally, its basic repeat unit is recognized to be composed of 4-*O*-3,6-anhydro-*α*-l-galactopyranose and 3-*O*-*β*-d-galactopyranose (illustrated in Fig. 14).

Agarose is derived by purifying agar. It is estimated that about 2/3 of the total agar extracted is agarose (Zhang et al. 2021). Structurally, agarose is constituted by repeating disaccharides with alternations on 3-linked *β*-d-galactopyranose and 4-linked 3,6-anhydro-*α*-l-galactopyranose residues (shown in Fig. 14). Some d-galactose and l-galactose units can be methylated, and pyruvate and sulfate are also found in small quantities in agarose composition (Zarrintaj et al. 2018; Guenet 2008). Currently, the gels of agarose have been used in many industrial applications, such as the food industry, molecular biology, microbiology, cell biology, and tissue engineering, due to thermoreversible behavior (thermal gelation) even at low concentrations, biocompatibility, high water content, biodegradability, non-toxicity together with high agarose availability and low-cost (B.S. Albuquerque et al. 2016; Yang et al. 2018). Additionally, agarose can be easily dissolved in different solvents, such as hot water (above 100 °C), dimethyl sulfoxide (DMSO), formamide (FA), dimethylformamide (DMF), *N*-methylformamide (MFA), and 1-butyl-3 methylimidazolium chloride (BmimCl) (Zarrintaj et al. 2018; Oliveira and Reis 2008).

#### Overview of the Extraction Methodology

##### Conventional Extraction Procedure

Conventionally, agar extraction comprises several steps, generally beginning with washing the raw materials and with an alkaline pre-treatment, which results in the desulfation of the polymer (Nishinari and Fang 2017). This pre-treatment is essential to eliminate the sulfate groups and promote the conversion of the units of l-galactose-6-sulfate into 3,6-anhydro-l-galactose, which is the main responsible for the improvements of agar gelling properties (Kumar and Fotedar 2009). Then, it is necessary to submerge the treated seaweeds in hot water for several hours to initiate the agar extraction. Additionally, after this step, it is essential for industrial purposes to perform a treatment with bleach to obtain a lighter shade of material, which is considered an optional step for laboratory purposes. However, an eco-friendly alternative is an urgent request to bleach the material on the agar extraction procedure (Li et al. 2008). After this, the residual water present in agar should be removed by a syneresis-based process through freezing followed by thawing several times, which is considered a good alternative instead of purchasing freeze-drying equipment on a large scale. To finalize, the agar materials should be completely dried using an oven and possibly stored at RT in the long term without compromising the integrity and properties of the agar. In addition, for future use, to prepare an aqueous agar solution, it is required to heat the material above its gel melting point, around 80–85 °C (Silva et al. 2012b).

Due to high demands for use in several applications and biotechnological areas, in the last decade, many efforts have been devoted to adapting existing agar extraction methods to ones that can be faster, with a higher yield, more eco-friendly, and cheaper. However, unfortunately, it has been impossible to assign a general extraction method valid for all agarophytes species enabling at the same time a standard quality for all industrial purposes. The conventional extraction procedure to obtain agar is demonstrated in Fig. 14.

##### Purification Process (Obtaining Agarose from Agar)

**Fig. 15:**
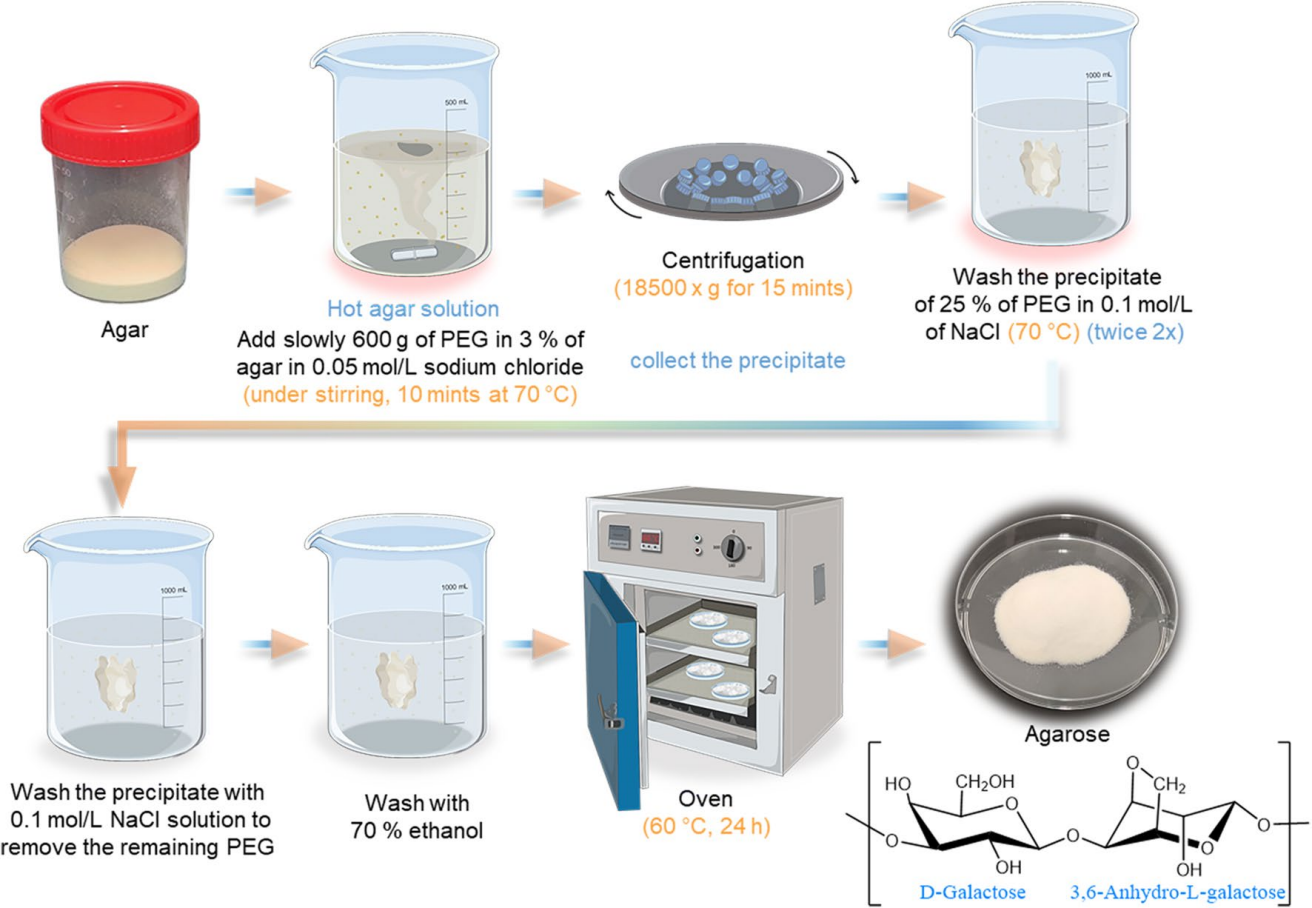
Schematic representation of the methodology to obtain agarose from agar, also represented agarose chemical structure, composed of d-galactose and 3–6-Anhydro-l-galactose

The principle of this process is to obtain a high level of pure agarose from the extracted agar. Currently, several methodologies are described to purify the agarose, such as (1) polyethylene glycol (PEG) precipitation, (2) DEAE-cellulose chromatography, and (3) PEG combined with DEAE cellulose chromatography (Zhang et al. 2019). In this review, we demonstrate the agarose purification process experimentally by PEG precipitation due to being easily performed in every ordinary laboratory (requiring no specific equipment) and being cheaper than other methodologies. The entire PEG procedure is demonstrated in Fig. 15.

A step-by-step practical guide of extraction and purification methodologies for the production of agarose from agar and this from red seaweeds (Tables S14 and S15) and a comprehensive inventory of the required materials, reagents, and equipment can be found in the supplementary information.

#### Analytical Methods for Polymer Characterization

Agarose boasts several essential physicochemical properties that necessitate assessment for its diverse applications. The most crucial factor is its gel strength, determined by its molecular weight and concentration, which is vital in electrophoresis techniques and chromatography. The degree of purity and the presence of contaminants must be determined to ensure accurate results in scientific experiments. Understanding the gelation temperature and its reversibility is key for applications in molecular biology. Agarose’s electroendosmosis, which influences the mobility of molecules in electrophoresis, also requires evaluation. Additionally, its solubility, rheological behavior, and interactions with biomolecules are important factors in formulation and research contexts. Evaluating these physicochemical properties is essential for harnessing the full potential of agarose in various scientific and biotechnological endeavors. Some of these physicochemical characterizations are herein explained. (1) Infrared spectroscopy. In infrared spectroscopy analysis, the basic backbone of agar typically shows broadband at 3300 cm^−1^ associated with the O–H groups, an absorbance at 2930 cm^−1^ related to the CH_2_ groups, and an absorbance between 2815–2845 cm^−1^, related to O-CH_3_ link vibrations. The absence of this last peak can indicate a lower methylation degree, which can be confirmed in ^13^C NMR spectra analysis (Guerrero et al. 2014). The band near 1640 cm^−1^ indicates the presence of proteins, i.e., assigned to amide I vibrations. The absorption bands at 1370 and 1250 cm^−1^ are associated with the vibration mode of sulfate groups. As explained above, these bands are related to the agar quality, since the presence of L-galactose-6-sulfate units decreases the gel strength (Kumar and Fotedar 2009). The band at 1180 cm^−1^ is correlated to the vibration mode of ester-sulfate link vibrations, and the intense peak that can be observed at 1040 cm^−1^ is common to all polysaccharides due to the coupling of the C-O or the C–C stretching modes with the C-O–H bending modes (Pereira et al. 2003). To finalize, the peak at 930 cm^−1^ is attributed to the vibration of the C–O–C bridge of 3,6-anhydro-galactose, while a small signal can appear at 850 cm^−1^, which is assigned to the C-O-S (sulfate) in C-4 link vibration from galactose (Mollet et al. 1998). (2) Molecular weight by High-temperature gel permeation chromatography (HT-GPC). The molecular weight of agar/agarose can be determined by HT-GPC equipped with two Shodex columns, using a flow rate of 0.6 mL/min at approximately 70 °C. For this, the samples should be dissolved (0.2% (w/v)) in a solvent with 0.2 M NaNO_3_ by previously heating the solvent in a boiling water bath (Suzuki et al. 2001). (3) Carbon 13 nuclear magnetic resonance (^13^C NMR) spectroscopy. The chemical structure of agar/agarose can be addressed by the potential proton of ^13^C-NMR spectroscopy. For this, the sample can be dissolved (5% (w/v) using the solvent, deuterated water (D_2_O), and recorded at 90 °C. As referenced in FTIR analysis, the presence or the absence of O-methyl groups can be noticed by the peak that should appear at approximately 59 ppm, which this complete absence indicates a lowly methylated structure (Guerrero et al. 2014). (4) Quantification of 3,6-anhydrogalactose contents. The fraction of 3,6-anhydro-galactose in the agar plays a fundamental role in regulating the helical conformation in the polysaccharide, which directly influences its gelation ability. Therefore, this can be quantified using the colorimetric method developed by Matsuhiro in 1983 (Matsuhiro and Zanlungo 1983). For this, the methodology consists of dissolving agar powder in distilled water at 90 °C and then mixing it in a solution containing 5% thymol dissolved in absolute ethanol and another solution with 0.5% ferric chloride dissolved in hydrochloric acid. To quantify the 3,6-anhydro-*α-d*-galactose contents, the measurements of the standard curve and the samples should be performed using absorbance at 635 nm with a spectrophotometer. (5) Quantification of sulfate contents. The sulfur contents of the obtained agar can be estimated using inductively coupled plasma optical emission spectrometry (ICP-EOS) or, for example, using the classic method of Gustafson (Guatafsson 1960). Firstly, the agar should be hydrolyzed using a solution of 1 M HCl at 100 °C and then add barium chloride (BaCl_2_), which links the sulfate ions in suspension with barium ions, promoting the precipitation of the compound hence formed. Next, the optical density of the standard solutions and the samples should be read at 550 nm using a spectrophotometer. (6) Quantification of gel strength. It is known that agar gel strength is influenced by several factors, such as polymer concentration, extraction methodology, and the pre-treatment, pH, and sugar contents such as the 3,6-anhydrogalactose, i.e., the gel strength increases with the increasing of the sugar contents (Ahmad et al. 2011). In this order, gel strength can be measured using a texture analyzer (e.g., TA-XT2 da Stable Micro Systems) and a complimentary rheometer analysis to measure the oscillatory and viscosity properties.

## Optimization Tools to Support Decisions When Defining New Methods on Polymers Extraction

Research for new marine-origin polymeric materials and the appropriate extraction methodologies is a field with many opportunities but still faces many challenges. Despite the vast knowledge (available in these areas), the most efficient production methods are still to be established for each raw material due to the complexity of some processes, amounts of variables, and different target features, among others. For this reason, the experimental design arises as a significantly helpful tool to conduct researchers/engineers/scientists to follow the best path and reach a process optimization, obtaining the desired product effectively and affordably. To be clear, the design of experiments (DoE) is not only about statistics but also a complementary mathematical tool capable of organizing and predicting the experiments systematically and efficiently examining the obtained data according to the selected parameters, indicating the best conditions to proceed. Furthermore, these tools have the advantage of interacting with different parameters to obtain the best points to increase the yield (or other aimed parameters) and avoid unnecessary and excessive characterizations and waste of products (Das and Dewanjee 2018). Therefore, DOE could be classified into four major factions: (i) factorial DOE, (ii) response surface methodology (RSM) based DOE, (iii) Latin square DOE, and (iv) Taguchi DOE (Vikram et al. 2014). However, the choice of the best DoE methodology depends on the researcher’s experience and other factors, like work objective, time, resources, and materials (among others) which together trace the best methodologies to be adapted to each experimental work. For instance, the optimization of the polymeric extraction process could be better performed by central composite or Box-Behnken DoE.

To be precise, Box-Behnken allows good accuracy in defining surface responses in relatively few experimental runs for one or more response variables (Ham and Jeswiet 2007). Although obtaining the data results is not linear, in the first analysis, the results from the diverse experiments must be carried out by relevance and quantitative answers to the statistical program to be effective in the design of 3D curves and drawing the Pareto charts. Therefore, it is crucial to accurately select the method that intends to be used to characterize the product in order to obtain a proper response. For instance, Vazquez et al. (2017) describe the process optimization for producing chitin and chitosan from *Illex argentinus* pen by-products. The optimal conditions for chitin isolation were determined for chemical and enzymatic deproteinization, and the resulting chitin was subsequently deacetylated by alkaline treatment. Moreover, Hifney et al. (2016) have focused on obtaining an efficient extraction of sulfated polysaccharides from brown algae, specifically fucoidan, using a hot buffer extraction process. The Box-Behnken experimental design was used to evaluate the impact of different conditions on fucoidan yield and its sulfate content.

## Concluding Remarks and Future Outlook

In the last decade, the sea has proven to be a vast source of materials, namely biopolymers, with a particular interest in several applications, including as an example, the food industry or medicine; as virucidal, bactericidal, and fungicidal agents, as well as biocompatible and biomedical materials due to their unique biochemical characteristics and properties. Many of these materials can be obtained from by-products generated in the industrial processing of marine biological resources, which have significantly increased in the last years. Taking advantage of the availability of these marine resources and the increasing demand for those polymeric products, many extraction methodologies have received significant attention for the obtention of polymers such as collagen, chitosan, fucoidan, or alginate (among others), with a high degree of confidence, to be used in human beings, many of them described in the manuscript. It is important to refer that when using by-products from marine resources, it is vital to consider the sustainability concerns that can arise. Overfishing, bycatch, resource depletion, environmental impact, and social and economic impacts should be respected. Thus, to certify sustainability, it is crucial to source by-products from well-managed and sustainable fisheries, minimize waste throughout the production process, and consider the larger context of promoting sustainable resource management and conservation of marine ecosystems; to support the long-term health and well-being of the oceans and respective communities. Scaling up the production of biomaterials from marine sources can be challenging due to several factors. Sourcing sustainable and abundant marine resources, extracting and purifying the biomaterials while removing impurities and contaminants, standardizing the production process, and cost-effectiveness are some of the concerns to consider. In this sense, the concept of biorefinery emerges. In fact, the biorefinery approach offers numerous opportunities for sustainable production processes using renewable resources. By using a wide range of feedstocks and extracting multiple products from a single source, biorefineries can increase resource efficiency, promote renewable energy sources, and produce a range of green chemicals. Biorefineries can also enable the transition to a circular economy by applying waste streams as feedstocks and producing biodegradable or recyclable materials.

In this protocol review, it is presented in a summarized and easy to interpret form the sources that provided the most common and explored marine origin polymers, their characteristics, chemical structure, and properties, as well as the most frequent extraction purification methodologies used to obtain them. Further, it states the materials, reagents, and equipment needed for each extraction procedure and the characterization and tools required to validate the extracted material. Despite the necessity of this review synthesis, many marine polymeric extraction methodologies exist in the literature. Most of them are similar to the methods presented in this article, only containing some modifications due to each author adapting to each raw material and personal preferences and know-how.

It is critical to address several challenges in the future to evolve in the field of polymer extraction. The progress of new alternatives to acidic/alkaline/solvent procedures and the optimization of current extraction methods are key factors for achieving greener approaches. This optimization will ensure that bioactive polymers can be extracted with high efficiency, reproducibility, and at a high level of purity and yield while employing low-cost, environmentally friendly techniques (using, for instance, less water and less energy) and preferably less time-consuming. Overcoming these challenges will lead to significant progress in the sustainable production of high-quality bioactive polymers with great potential for various industrial applications.

## Supplementary Information

Below is the link to the electronic supplementary material.

Supplementary file1 (DOCX 120 kb)

## Data Availability

All data generated or analyzed during this study are included in the article or available from the corresponding author on reasonable request.
