# Supplementary material for: Extraction and Purification of Biopolymers from Marine Origin Sources Envisaging Their Use for Biotechnological Applications
Source: Mar Biotechnol (NY). 2024 Sep 10;26(6):1079–119. doi: 10.1007/s10126-024-10361-5 (PMC11541305; doi:10.1007/s10126-024-10361-5)
Supplement: Supplementary file 1 — Supplementary file1 (DOCX 120 kb) [file 10126_2024_10361_MOESM1_ESM.docx]

**Extraction and purification of biopolymers from marine origin sources envisaging their use for biotechnological applications**

Duarte Nuno Carvalho^1,2^, Cristiana Gonçalves^1,2^, Rita O. Sousa^1,2^, Rui L. Reis^1,2^, J. Miguel Oliveira^1,2^, Tiago H. Silva^1,2^*

^1^3B´s Research Group, I3B´s – Research Institute on Biomaterials, Biodegradables and Biomimetics of University of Minho, Headquarters of the European Institute of Excellence on Tissue Engineering and Regenerative Medicine, AvePark 4805-017, Barco, Guimarães, Portugal

^2^ICVS/3B´s – PT Government Associate Laboratory, Braga/Guimarães, Portugal

*Corresponding author: Tiago H. Silva

E-mail: [tiago.silva@i3bs.uminho.pt](mailto:tiago.silva@i3bs.uminho.pt)

**Suplementary information**

**A practical guide of extraction and purification methodologies (step-by-step protocols) and a comprehensive inventory of necessary materials, reagents, and equipment for the extraction of diverse marine origin biopolymers**

**S1. Collagen and Gelatin**

*S1.1. Acidic solution extraction method*

**Table S1.** Comprehensive collagen extraction protocol using acidic procedure: a Step-by-Step Guide.

| **Step** | **Description** |
| --- | --- |
| ◷ TIMING ~3 weeks (10 days active (accumulative days), one overnight precipitation, and 5 days minimum on freeze-drier).  ‼ Caution All the following steps, including solutions and conditions, must be carried out at 4 °C to avoid collagen denaturation (for many marine origin collagens, denaturation temperature is below 22 °C). | |
| 1 | The raw materials of interest need to be cleaned from the rest of the soft tissue, as muscle and fat debris, washed, and cut into small pieces.  ! Advice There is no standard cleaning process in this step. This methodology can be adapted according to the source anatomy. |
| 2 | Removal of non-collagenous proteins, mixing with 0.1 M NaOH using a ratio 1:10 (w/v), under stirring for 1 day (change the solution 3 times). The treated biomass can be recovered by filtration with gauze and washed with abundant and cold distilled water until a neutral or weak basic ph.  ! Advice The NaOH concentration can be modified depending on the raw material used. Other enzymes may also be used, but pepsin is the most common to cut the talopeptides. |
| 3 | Decalcification or Demineralization can be performed using Ethylenediamine tetraacetic acid (EDTA) or Hydrochloric acid (HCl) solution with a ratio of 1:10 (w/v), under stirring. The solution is changed every 8 h. Then, the demineralized hard tissue is continuously stirred with 20 volumes of cold tap water for 10 min (3x). (Optional step)  ! Advice This step is required when the collagen is isolated from scales, bones, or calcified cartilage. The decalcification solution and reaction time can be changed according to the hard tissue used. |
| 4 | The raw material can be defatted using 10 % 2-propanol with a 1:10 (w/v) ratio for 24 h under stirring. Then, the biomass is filtered using gauze and washed with abundant and cold distilled water until the washed water becomes clear. (Optional step)  ! Advice This step is required when the raw materials present more fats. The solution and the reaction time can be switched according to the tissue used. |
| 5 | Collagen extraction is achieved by incubating the pre-treated raw material in 0.5 M acetic acid solution (1:10 w/v), for 3 days, at 4 °C, under stirring, followed by centrifugation at 20000 x g for 30 min at 4 °C.  ! Advice In this step, if possible, it is better to grind (a little, not until powder) to facilitate the process. |
| 6 | Collagen extraction can also be achieved by protease digestion for pepsin soluble collagen extraction, placing the pre-treated raw material in 0.5 M acetic acid solution (1:10 w/v) containing 10 % pepsin (according to the dry weight), for 3 days, at 4 °C, under stirring, followed by centrifugation at 20000 x g during 30 min at 4 °C.  ! Advice The digestion enzyme ratio can be changed depending on the tissue used. |
| 7 | The resultant pellet from steps 5 and 6 can undergo a re-extraction. |
| 8 | To precipitate the collagens, the supernatants from steps 5 and 6 are salted out by adding NaCl to a final concentration of 2.6 M in 0.05 M Tris–HCl (pH 7.5) and left overnight, under stirring at 4 ºC. The precipitate was removed by centrifugation at 20000 x g for 30 min at 4 °C.  ! Advice Different precipitation solutions are adapted in each extraction technique. |
| 9 | The pellet is resuspended in 0.5 M of acetic acid and dialyzed first against 0.1 M acetic acid for 2 days, then against 0.02 M acetic acid for 2 days, and finally against distilled water until pH 7. |
| 10 | The collagen solution is frozen at -80 ºC overnight and placed on a freeze-dryer.  ◼ Pause Point The dry material can be stored indefinitely at room temperature (RT). |
| 11 | After this step, the percentage (%) extraction yield of collagen can be determined as the ratio between the final product (after freeze-drying weight of the extracted collagen) and the initial product (weight of by-products biomass, preferably dry) using the following **Equation S1.** $\text{Extraction yield of collagen \% = }\frac{\text{Weight of collagen (g)}}{\text{Weight of biomass (g)}}\text{ x 100}$  **Equation S1.** |

*S1.2. Alkaline solution extraction method*

**Table S2.** Comprehensive collagen extraction protocol using alkaline procedure: a Step-by-Step Guide.

| **Step** | **Description** |
| --- | --- |
| ◷ TIMING ~2 weeks [3 days active (accumulative days), and 3 days minimum on freeze-drier]  ‼ Caution All the following steps must be carried out at 4 °C to avoid collagen denaturing. | |
| 1 | The sponges are thawed, and any exogenous materials are removed by rinsing in dH_2_O and cut into small pieces.  ! Advice This cleaning procedure can be adapted to marine sponge species. The excess water needs to be poured from the marine sponge samples. |
| 2 | The disaggregating solution (50 mM Tris–HCl buffer pH 7.4, 1 M NaCl, 50 mM EDTA, and 100 mM 2-mercaptoethanol) are added and left under stirring for 4 days.  ‼ Caution Be careful when handling 2-mercaptoethanol as it is considered toxic; use the appropriate material and equipment, and follow the associated safety rules. |
| 3 | The collagen solutions are filtered with a nylon mesh to remove any remaining undissolved fragments.  ‼ Caution The solution contains 2-mercaptoethanol; it must be handled according to the safety protocol. |
| 4 | The solution is widely dialyzed for 7 days against dH_2_O (change 2 times per day) to remove all traces of 2-mercaptoethanol.  ‼ Caution The solution contains 2-mercaptoethanol, it must be handled according to the safety protocol. |
| 5 | The suspensions are centrifuged at 1200 x g for 10 min at 4 ºC to further remove cell debris and sand particles. |
| 6 | To collect the collagen fibrils from the suspension, another centrifugation is followed at 12100 x g for 30 min at 4 ºC.  ! Advice Yielding pellets containing collagen can be resuspended in dH_2_O and stored at 4 ºC to be used further. |
| 7 | The collagen re-extraction is performed by repeating the second centrifugation step. |
| 8 | Freeze the collagen solution at -80 ºC overnight and put it on a freeze-dryer.  ‼ Caution The dry material can be stored at RT indefinitely. |
| 9 | The collagen percentage (%) extraction yield can be calculated using **Equation S1.** |

*S1.3. General inventory of materials, reagents, and equipment for collagen extraction*

*S1.3.1 Materials*

**▪** Stainless steel scissors (Step 1 in both methodologies).

**▪** Scalpel (Step 1 in both methodologies).

**▪** Cutting board (Step 1 in both methodologies).

**▪** Glass or plastic beaker 5 L (Steps 2 to 9 in the acidic methodology, and steps 2 and 4 in the alkaline methodology).

**▪** Stir bars (Steps 2 to 9 in the acidic methodology and steps 2 and 4 in the alkaline methodology).

**▪** Spatula (Steps 2, 3, 6, and 8 in the acidic methodology, and steps 2 and 6 in the alkaline methodology).

**▪** pH paper (Step 9 in the acidic methodology).

**▪** Centrifugal tubes 50 mL (Steps 5-8 in the acidic methodology and steps 5 and 6 in the alkaline methodology).

**▪** Gauze (Steps 2 to 4 in the acidic methodology).

**▪** Nylon meshes (Step 3 in the alkaline methodology).

**▪** Strainer (Step 1 in both methodologies).

*S1.3.2. Reagents*

**▪** Sodium hydroxide (NaOH) (Step 2 in the acidic methodology).

**▪** Hydrochloric acid (HCl) (Step 3 in the acidic methodology).

**▪** Acetic acid (CH_3_COOH) (Steps 5, 6, 7, and 9 in the acidic methodology).

**▪** EDTA (C_10_H_16_N_2_O_8_) (Step 3 in the acidic methodology and step 2 in the alkaline methodology).

**▪** 2-propanol (C_3_H_8_O) (Step 4 in the acidic methodology).

**▪** Sodium chloride (NaCl) (Step 8 in the acidic methodology and step 2 in the alkaline methodology).

**▪** Tris-HCl (C_4_H_11_NO_3_) (Step 8 in the acidic methodology and step 2 in the alkaline methodology).

**▪** 2-mercaptoethanol (C_2_H_6_OS) (Step 2 in the alkaline methodology).

*S1.3.3. Equipment*

**▪** Analytical balance (Steps 1, 6, and 11 in the acidic methodology, and steps 1, 2 and 9 in the alkaline methodology).

**▪** Freeze-dryer (Step 10 in the acidic methodology and step 8 in the alkaline methodology).

**▪** Magnetic stirrer (Steps 2 to 9 in the acidic methodology and steps 2 and 4 in the alkaline methodology).

**▪** Freezer -80 ºC (Step 10 in the acidic methodology and step 8 in the alkaline methodology).

**▪** Centrifuge (Steps 5 to 8 in the acidic methodology and steps 5 and 6 in the alkaline methodology).

**▪** pH meter (Step 8 in the acidic methodology and step 2 in the alkaline methodology).

**▪** Cold room (4 °C) ! Warning Preferably in all steps to avoid collagen denaturation.

**S2. Chitin and chitosan**

*S2.1. Conventional extraction procedure*

**Table S3.** Comprehensive chitin/chitosan extraction protocol using convencional procedure: a Step-by-Step Guide.

| **Step** | **Description** |
| --- | --- |
| ◷ TIMING ~6 days (3 days active, one overnight dry, and 3 days minimum on freeze-drier) | |
| 1 | The raw materials of interest need to be cleaned from the rest of the soft tissue, washed, and dried.  ! Advice There is no standard cleaning process in this step. This methodology can be adapted according to the source anatomy. |
| 2 | Cut the materials into small pieces. |
| 3 | Grind the small pieces using an ultra-centrifugal mill to obtain a powder material.  ‼ Caution For this procedure that uses an ultra-centrifugal mill, the raw materials should be completely dry. (Optional step)  ! Advice This step facilitates the efficiency of the process and the purity of the final product. |
| 4 | Weight the dry material. |
| 5 | To promote demineralization, 10 g of powder obtained is slowly added into the glass beaker containing 1M of HCl to obtain a ratio of 1:10 w/v, with constant stirring (~150 rpm) at RT for 2 h. This step consists in removing the mineral matter bound to chitin.  ‼ Caution The acid must be slowly added due to the gas formation. This kind of material contains calcium carbonate that reacts with the acid, forming carbon dioxide. |
| 6 | Centrifugate the solution at 18500 x g for 15 min to recover the sample, wash it with distilled water to neutralize the pH, and dry the material in an oven at 60 °C overnight. |
| 7 | For deproteinization, demineralized material is treated with 1M of NaOH (1:20 w/v) in a constant stirring (~150 rpm) at 80 ºC for 22 h. This step is essential to remove the proteins. |
| 8 | Centrifuge again, wash with distilled water until neutralizing the pH and dry in an oven at 60 ºC for 24 h.  ◼ Pause Point The dry material can be stored at RT. |
| 9 | After this step, the end product is chitin. The percentage (%) isolation/production yield of chitin can be calculated using **Equation S2**. $\%\text{ yield of chitin = }\frac{\text{weight of isolated chitin}}{\text{weight of starting material}}\text{ }\times\text{ 100}$  **Equation S2.** |
| 10 | To obtain chitosan, 1 g of the chitin is added to 50 % NaOH (w/v) under constant stirring for 1 h at 65 ºC. |
| 11 | Filter the solution with filter paper using a vacuum pump. |
| 12 | Freeze the material at -80 °C overnight and then place it on a freeze-dryer.  ◼ Pause Point The dry material can be stored at RT indefinitely. |
| 13 | After this step, the end product is chitosan (the weight of the chitosan should be determined). |

*S2.2. Simpler and faster production procedure*

**Table S4.** Comprehensive chitin/chitosan extraction protocol using faster procedure: a Step-by-Step Guide.

| **Step** | **Description** |
| --- | --- |
| ◷ TIMING ~4 days (1 day active, one overnight dry, and 3 days minimum on freeze-drier) | |
| 1 | Steps 1, 2, and 3 on the conventional extraction procedure (Figure 3 and Table 3). |
| 2 | Dissolve 10 g of powder raw material in 50 % (w/v) NaOH, under constant stirring at RT, overnight.  ! Warning The powder needs to be added slowly to avoid granules. |
| 3 | Prepare a bath with mineral oil in a crystallizing dish, preheat it to 75 ºC, and submerge the round bottom flask containing the dissolved solution. The system must be constantly stirring for 2 h with a nitrogen atmosphere (N_2_).  ! Advice Use two needles on top of the round bottom flask to create a continuous N_2_ flow inside of the flask.  ! Warning The stirring in this step is crucial to provide a homogenous contact between the N_2_ flow and the solution.  ! Warning The chitosan deacetylation degree can be manipulated on this step using less or more reaction time (*i.e.,* tuning the extension of the reaction). |
| 4 | Filter the solution with filter paper using a vacuum pump. |
| 5 | Wash the filtered material with distilled water until pH reaches 7.  ! Advice Use one strainer with an individual mesh filter nylon membrane (100 µm) on top of the strainer to avoid unnecessary loss of material. |
| 6 | Freeze the material at -80 °C overnight and then put it on a freeze-dryer. |
| 7 | After this step, the end product is chitosan.  ◼ Pause Point The dry material can be stored at RT. |
| 8 | It is optional to make a purification process on this chitosan since this methodology provides a highly purified product. |
| 9 | Step 12 on the conventional extraction procedure. |

*S2.3. Purification process*

**Table S5.** Comprehensive chitin/chitosan purification protocol: a Step-by-Step Guide.

| **Step** | **Description** |
| --- | --- |
| ◷ TIMING ~5 days (7-8 hours active, one overnight dry, and 3 days minimum on freeze-drier) | |
| 1 | Prepare 1 L of acetic acid solution (2 wt%, v/v) in a glass beaker. |
| 2 | Slowly add 10 g of chitosan powder into the glass beaker containing the acetic acid solution to obtain 1 wt% chitosan solution, under stirring. |
| 3 | Leave the solution overnight under stirring at RT. |
| 4 | Filter the chitosan solution twice. The first time (1x), use a nylon filter and then a Millipore membrane (5 μm) to remove the sludges (remove non-soluble impurities).  ! Warning If there is an obstruction of the nylon filter, remove it and wash it with an acetic acid solution (2 wt%). After that, wash the filter with distilled water several times. |
| 5 | After the filtration steps, transfer the filtered solution to a glass beaker under stirring at RT. |
| 6 | Slowly add 2 M NaOH, under stirring, until adjusting the pH to 8 to promote the polymer's precipitation, preferably during 30 min. |
| 7 | Filter the chitosan precipitated material. |
| 8 | Wash the precipitated material with distilled water until pH reaches 7.  ! Advise The chitosan precipitate can be transferred to a glass beaker with distilled water, under stirring for 5 minutes at RT, to help the deagglomeration of the precipitate material. |
| 9 | When the pH reaches 7, the chitosan precipitate is passed through ethanol-water solutions (20/80 and 50/50, and 90/10), 5 minutes under stirring in each solution, for dehydration.  ! Warning After that, the chitosan should be filtered to remove the maximum ethanol solution. |
| 10 | Transfer the purified chitosan to large Petri dishes or centrifuge tubes 15 mL ), depending on the available material/equipment).  ! Advise The centrifuge tubes can be put into round bottom flasks for freeze-drying. |
| 11 | Freeze the material at -80 °C overnight and then put it on a freeze-dryer. |
| 12 | After that, grind the material.  ! Warning The purified chitosan should be stored in a plastic bottle.  ◼ Pause Point The dry material can be stored at RT. |

*S2.4. General inventory of materials, reagents, and equipment for chitin and chitosan extraction*

*S2.4.1. Materials*

**▪** Stainless steel scissors (Step 2 in conventional ext. and step 1 in faster ext.).

**▪** Scalpel (Step 2 in conventional ext. and step 1 in faster ext.).

**▪** Cutting board (Step 2 in conventional ext. and step 1 in faster ext.).

**▪** Crystallizing dish (Step 3 in faster ext.).

**▪** Glass beaker (Steps 5, 7, and 10 in conventional ext., step 2 in faster ext., and steps 2, 5, 8, and 9 in purification).

**▪** Hammer (Steps 1 and 2 in conventional ext. and step 1 in faster ext.).

**▪** Round bottom flask (Step 10 in conventional ext. and step 3 in faster ext.).

**▪** Stir bars (Steps 2, 3, and 10 in conventional ext., steps 2 and 3 in faster ext., and steps 1, 3, 6, 8, and 9 in purification).

**▪** Spatula (Steps 3, 5, 7, and 10 in conventional ext., steps 1, 2, and 5 in faster ext., and steps 2, 6, 8, 9, and 10 in purification).

**▪** pH paper (Step 5 in faster ext., and step 8 in purification).

**▪** Centrifugal tubes 15 mL (Step 10 in purification).

**▪** Centrifugal tubes 50 mL (Steps 6 and 8 in conventional ext.).

**▪** Petri dish (Step 10 in faster ext.).

**▪** Filter paper (Step 11 in conventional ext., step 4 in faster ext., and steps 7 and 9 in purification).

**▪** Filter nylon membrane 100 μm (Step 5 in faster ext., and steps 4 and 8 in purification).

**▪** Needle (Steps 2 and 3 in faster ext.).

**▪** Strainer (Step 5 in faster ext., and steps 8 and 9 in purification).

**▪** Plastic beaker 5 L (Step 5 in faster ext., and step 8 in purification).

**▪** Millipore membrane 5 μm (Step 4 in faster ext.).

**▪** Plastic bottle 100 mL (Steps 3 and 13 in conventional ext., step 1 in faster ext., and step 12 in purification).

**▪** Büchner flask (Step 11 in conventional ext., step 4 in faster ext., and steps 4 and 7 in purification).

**▪** Büchner funnel (Step 11 in conventional ext., step 4 in faster ext., and steps 4 and 7 in purification).

**▪** Mineral oil (Step 3 in faster ext.).

*S2.4.2. Reagents*

**▪** Sodium hydroxide (NaOH) (Steps 7 and 10 in conventional ext., step 2 in faster ext., and step 6 in purification).

**▪** Hydrochloric acid (HCl) (Step 5 in conventional ext., step 1 in faster ext., and step 1 in purification).

**▪** Acetic acid (CH_3_COOH) (Step 1 in conventional ext.).

**▪** Ethanol (C_2_H_5_OH) 96 % (Step 9 in purification).

*S2.4.3. Equipment*

**▪** Analytical balance (Steps 4, 5, 9, 10, and 13 in conventional ext. and step 1 in faster ext.).

**▪** Ultra-centrifugal mill (Step 3 in conventional ext. and step 1 in faster ext.).

**▪** Analytical sieve shaker (Step 3 (optional) in conventional ext. and step 1 in faster ext.).

**▪** Oven 60 ºC (Step 6 in conventional ext., step 1 in faster ext., and step 1 in purification).

**▪** Freeze-dryer (Step 12 in conventional ext., step 6 in faster ext., and step 11 in purification).

**▪** Magnetic stirrer with heater system (Steps 5, 7, 10 in conventional ext., steps 2 and 3 in faster ext., and steps 3, 6, and 9 in purification).

**▪** Freezer -80 ºC (Step 12 in conventional ext., step 6 in faster ext., and step 11 in purification).

**▪** Centrifuge (Steps 6 and 8 in conventional ext.).

**▪** pH meter (Step 6 in purification).

**▪** Vacuum pump (Step 11 in conventional ext., step 4 in faster ext., and step 4, 7, and 9 in purification).

**▪** Compressive nitrogen (N_2_) air (Step 3 in faster ext.).

**S3. Fucoidan**

*S3.1. Hot water extraction method*

**Table S6.** Comprehensive fucoidan hot water extraction protocol: a Step-by-Step Guide.

| **Step** | **Description** |
| --- | --- |
| ◷ TIMING ~2 weeks (no less than 7 days to dry the macroalgae, 2 days active, 4 days overnight dry, and 3 days minimum on freeze-drier) | |
| 1 | If the macroalgae raw material is provided directly from the sea, it needs to be cleaned and washed several times with distilled water to remove other biological materials such as macro- and micro-algae from other species, sand, and small animals. (Optional step) |
| 2 | Let the macroalgae dry completely at RT using absorbent paper. Then, the percentage moisture contents can be determined using **Equation S3**. (Optional step)  ! Warning Change several times the absorbent paper to prevent the appearance of fungi on the surface of the macroalgae. $\text{Moisture percentage in (\%) = }\frac{m_{\text{m}}-\text{ }m_{\text{d}}}{m_{\text{d}}}\text{ × 100}$  **Equation S3.**  where m_m_ is the wet weight mass, and m_d_ is the dried weight mass of the macroalgae.  ! Advice Steps 1 and 2 are optional when the raw material is bought already dried. However, this material should be maintained in a dry atmosphere (e.g., an adequate vacuum bag). |
| 3 | Cut the materials into small pieces. |
| 4 | Add twenty grams (20 g) of the cut brown macroalgae into a beaker containing 1 L of 85 % ethanol under constant stirring for 12 h at RT.  ! Warning This step is very important to remove residual dirt, pigments, lipids, proteins, and low molecular weight compounds. |
| 5 | Wash the material with acetone and let it dry at RT to remove lipids and pigments. |
| 6 | Hot water extraction. Add five grams (5 g) of the dried biomass in 100 mL of distilled water at 65 °C with continuous stirring for 1 h.  ! Warning This procedure should be performed twice (2x). |
| 7 | Centrifugate the material at 18500 x g for 15 minutes.  ! Advice Adaptive step, the time can be increased or decreased according to the pellet formation. |
| 8 | Add 1 % of calcium chloride (CaCl_2_) to the supernatant obtained. After that, the solution should be maintained at 4 °C overnight to precipitate the alginate residues. |
| 9 | Centrifugate the material at 18500 x g for 15 minutes. |
| 10 | Add 99 % ethanol to the supernatant to obtain a final concentration of 30 % and leave the solution at 4 °C for 4 h. |
| 11 | Centrifugate the material at 18500 x g for 15 minutes to remove the remaining non-dissolved impurities (discard the pellet). |
| 12 | Add 99 % ethanol to the collected supernatant to obtain a final concentration of 70 %. After that, the solution should be maintained at 4 °C overnight to precipitate out the intact fucoidan. |
| 13 | Centrifugate the material at 18500 x g for 15 minutes. |
| 14 | Wash the pellet with 99 % ethanol and acetone and dry using a [desiccator](https://www.google.com/search?client=firefox-b-d&sxsrf=AOaemvJ2N-4G01RR6tjyKkXBlclWiLAiWA:1637657559413&q=desiccator&spell=1&sa=X&ved=2ahUKEwj0pd7oja70AhWOasAKHWYnClYQBSgAegQIARA2) vacuum at RT overnight. |
| 15 | Dialysis against pure water under a constant stirring at RT. (Optional step) |
| 16 | Freeze the material at -80 °C overnight and then freeze-dry it. The percentage (%) yield of extracted fucoidan can be calculated using the ratio between the final product (after freeze-drying) and the weight of the initial raw material (dried macroalgae biomass), following **Equation S4**. $\text{Yield of Fucoidan (\%) = }\frac{\text{Weight of fucoidan (g)}}{\text{Weight of the dried biomass (g)}}\text{ × 100}$  **Equation S4.** |

*S3.2. Acidic extraction method*

**Table S7.** Comprehensive fucoidan acidic extraction protocol: a Step-by-Step Guide.

| **Step** | **Description** |
| --- | --- |
| ◷ TIMING ~2 weeks (no less than 7 days to dry the macroalgae, 2 days active, and 4 days overnight dry) | |
| 1 | Steps 1, 2, and 3 on the conventional water extraction procedure. |
| 2 | Add 10 g of the cut brown macroalgae into a round-bottom flask and reflux 100 mL of 99 % ethanol for 2 h at 80 °C to remove fat and color pigments. |
| 3 | Centrifugate the solution at 18500 x g for 10 minutes (discard the supernatant). |
| 4 | Let the macroalgae dry overnight using a [desiccator](https://www.google.com/search?client=firefox-b-d&sxsrf=AOaemvJ2N-4G01RR6tjyKkXBlclWiLAiWA:1637657559413&q=desiccator&spell=1&sa=X&ved=2ahUKEwj0pd7oja70AhWOasAKHWYnClYQBSgAegQIARA2) with the vacuum at RT. |
| 5 | Put five grams (5 g) of the defatted algae into a beaker containing 100 mL of 0.15 M HCl under constant stirring for 2 h at 65 °C. |
| 6 | Centrifugate the solution at 18500 x g for 10 minutes. |
| 7 | The supernatant needs to be neutralized with 3 M of NaOH.  ! Warning This step is essential to prevent any acidic damage to the fucoidan structure during storage. |
| 8 | Store the neutralized extract at 4 °C overnight. |
| 9 | Four volumes of absolute ethanol need to be added into the extract solution and stored overnight at 4 °C to precipitate the fucoidan. |
| 10 | Centrifugate the solution at 18500 x g for 10 minutes to recover the fucoidan. |
| 11 | Remove the precipitate from the centrifuge tubes using absolute ethanol. |
| 12 | Let the precipitate dry overnight using a [desiccator](https://www.google.com/search?client=firefox-b-d&sxsrf=AOaemvJ2N-4G01RR6tjyKkXBlclWiLAiWA:1637657559413&q=desiccator&spell=1&sa=X&ved=2ahUKEwj0pd7oja70AhWOasAKHWYnClYQBSgAegQIARA2) with vacuum at RT. |
| 13 | Grind the fucoidan using a mortar and pestle. After that, the fucoidan extract can be stored at RT. |

*S3.3. Salt extraction method*

**Table S8.** Comprehensive fucoidan salt extraction protocol: a Step-by-Step Guide.

| **Step** | **Description** |
| --- | --- |
| ◷ TIMING ~3 weeks (no less than 7 days to dry the macroalgae, 3 days active, 2 days overnight, and 3 days minimum on freeze-drier) | |
| 1 | Steps 1, 2, and 3 on the conventional water extraction procedure. |
| 2 | Grind the small pieces using an ultra-centrifugal mill to obtain a powder material.  ‼ Caution For this procedure that uses an ultra-centrifugal mill, the raw materials should be completely dry. |
| 3 | To perform a pre-treatment, 10 g of milled algae are put into a beaker with 100 mL of methanol, chloroform, and water (4:2:1) under constant stirring to remove fat, protein, and color pigments. |
| 4 | Filter the solution with filter paper (90 mm) using a vacuum pump. |
| 5 | Wash the filtered material with acetone and let it dry at RT overnight. |
| 6 | Put the dried material into a beaker with 100 mL of 2 % CaCl_2_ under constant stirring for 5 h at 85 °C to extract water-soluble polysaccharides. |
| 7 | Filter the solution again with filter paper (90 mm) using a vacuum pump. |
| 8 | Mix the filtered material with 30 mL of 10 % cetavlon at 4 °C overnight to promote the fucoidan precipitation. |
| 9 | Centrifugate the solution at 18500 x g for 15 minutes (discard the supernatant). |
| 10 | Wash the precipitate material with distilled water and stir with 50 mL of 20 % ethanolic sodium iodide for 3 days at RT to remove and decompose any cetavlon residue. |
| 11 | Centrifugate the solution at 18500 x g for 15 minutes (discard the supernatant). |
| 12 | Wash the precipitate with absolute ethanol to remove sodium iodide. |
| 13 | Freeze the material overnight at -80 °C and then freeze-dry it. Same procedure and calculation of yield percentage from step 16 on the conventional water extraction method. |

*S3.4. General inventory of materials, reagents, and equipment for fucoidan extraction*

*S3.4.1. Materials*

**▪** Lab plastic tray (Steps 1 and 2 in hot water ext., step 1 of acid and salt ext.).

**▪** Absorbent paper (Step 2 in hot water ext., step 1 of acid and salt ext.).

**▪** Spatula (Steps 1, 6, and 8 in hot water ext., steps 1, 2, and 5 in acid ext., and step 3 in salt ext.).

**▪** Stainless steel scissors (Step 3 in hot water ext., and step 1 in acid and salt ext.).

**▪** Scalpel (Step 3 in hot water ext., and step 1 of acid and salt ext.).

**▪** Cutting board (Step 3 in hot water ext., and step 1 of acid and salt ext.).

**▪** Glass beaker (Steps 4, 5, 6, 10, and 14 in hot water ext., step 5 in acid ext., and steps 3, 5, 6, 8, 10, and 12 salt ext.).

**▪** Round bottom flask (Step 2 in acid ext.).

**▪** Desiccator vacuum (Step 14 in hot water ext., and step 3 and 12 in acid ext.).

**▪** Stir bars (Steps 4, 6, and 15 in hot water ext., step 5 in acid ext., and steps 3 and 6 in salt ext.).

**▪** Mortar and pestle (Step 13 in acid ext.).

**▪** Pipette Pasteur (Step 7 in hot water ext.).

**▪** Centrifugal tubes 50 mL (Steps 7, 9, 11, and 13 in hot water ext., steps 3, 6, and 10 in acid ext., and steps 9 and 11 in salt ext.).

**▪** Filter paper (Steps 4 and 7 in salt ext.).

**▪** Plastic urine container 100 mL (Step 16 in hot water ext., step 13 in acid ext., and step 13 in salt ext.).

**▪** Aluminium paper (Steps 4, 8, 15, and 16 in hot water ext., step 5 in acid ext., and steps 3, 5, 6, 8, and 13 in salt ext.).

**▪** Büchner flask (Steps 4 and 7 in salt ext.).

**▪** Büchner funnel (Steps 4 and 7 in salt ext.).

**▪** Dialysis membrane (Step 15 in hot water ext.).

! Warning The selection of dialysis pore size variates according to each fucoidan molecular weight.

*S3.4.2. Reagents*

**▪** Sodium hydroxide (NaOH) (Step 7 in acid ext.).

**▪** Hydrochloric acid (HCl) (Step 5 in acid ext.).

**▪** Calcium chloride (CaCl_2_) (Step 8 in hot water ext., and step 6 in salt ext.).

**▪** Ethanol (C_2_H_5_OH) 85 % (Step 4 in hot water ext.).

**▪** Absolute ethanol (C_2_H_5_OH) 99 % (Steps 10, 12, and 14 in hot water ext., steps 2, 9, and 11 in acid ext., and step 12 in salt ext.).

**▪** Acetone (C_3_H_6_O) (Steps 5 and 14 in hot water ext., and step 5 in salt ext.).

**▪** Methanol (CH_3_OH) (Step 3 in salt ext.).

**▪** Chloroform (CHCl₃) (Step 3 in salt ext.).

**▪** Cetavlon (antiseptic pharmacologic drug) (Step 8 in salt ext.).

**▪** Sodium iodide (NaI) (Step 10 in salt ext.).

*S3.4.3. Equipment*

**▪** Analytical balance (Steps 2, 4, 6, 8, and 16 in hot water ext., steps 1, 2, 5, and 7 in acid ext., and steps 1, 3, 6, 10, and 13 in salt ext.).

**▪** Ultra-centrifugal mill (Step 2 in salt ext.).

**▪** Magnetic stirrer with heater system (Steps 4, 6, and 15 in hot water ext., step 5 in acid ext., and steps 3, 6, and 10 in salt ext.).

**▪** Electric heating mantle at 80 °C (Step 2 in acid ext.).

**▪** Fridge 4 °C (Steps 8, 10, and 12 in hot water ext., steps 8 and 9 in acid ext., and step 8 in salt ext.).

**▪** Freezer -80 ºC (Step 16 in hot water ext., and step 13 in salt ext.).

**▪** Freeze-dryer (Step 16 in hot water ext., and step 13 in salt ext.).

**▪** Centrifuge (Steps 7, 9, 11, and 13 in hot water ext., steps 3, 6, and 10 in acid ext., and steps 9 and 11 in salt ext.).

**▪** pH meter (Step 7 in acid ext.).

**▪** Vacuum pump (Step 14 in hot water ext., steps 4 and 12 in acid ext., and steps 4 and 7 in salt ext.).

**S4. Carrageenan**

*S4.1. Extraction method*

**Table S9.** Comprehensive carrageenan alkaline extraction protocol: a Step-by-Step Guide.

| **Step** | **Description** |
| --- | --- |
| ◷ TIMING ~1 week (24 h to dry the macroalgae, 5 days active, 1 day overnight dry) | |
| 1 | The raw macroalgae must be cleaned and washed several times with distilled water to eliminate salt and debris from the thallus surface. |
| 2 | Let the macroalgae dry to constant weight in an oven at 60 °C for 24 h minimum. Then, the percentage of moisture contents can be determined using **Equation S5**. $\text{Moisture contents (\%) = }\frac{m_{\text{m}}-\text{ }m_{\text{d}}}{m_{\text{d}}}\text{ × 100}$  **Equation S5.**  where m_m_ is the wet weight mass, and m_d_ is the dried weight mass of the macroalgae. |
| 3 | Grind the raw material to render the samples uniform. |
| 4 | As pre-treatment, add 2 × 1 g of the dry seaweed into a beaker containing a mixture of acetone/methanol (75 mL of each reagent) under constant stirring for 12 h at RT.  ! Warning This step is essential to eliminate the organo-soluble fraction . |
| 5 | Place the sample into a solution of NaOH (1 M) in a ratio of 150 mL/g, under constant stirring between 3 to 4 h at a temperature between 80 °C to 85 °C, to extract carrageenan. |
| 6 | The solution needs to be neutralized to pH 6-8 with 0.3 M HCl. |
| 7 | Filter twice (2x) the hot solution under vacuum through cloth and glass fiber filter to remove the remaining biomass. |
| 8 | Concentrate the extracts with a rotary evaporator under vacuum until reaching one-third of the initial volume. |
| 9 | To precipitate the carrageenan, add to the concentrated solution, under warm conditions, twice its volume of 96 % ethanol. |
| 10 | Pull out the extracted carrageenan using a glass rod for a clean glass or petri dish. Squeeze the material to drain the exceeded liquid. |
| 11 | Add 100 mL of absolute alcohol to the material under constant stirring for 12 to 24 h. |
| 12 | Dry the carrageenan in an oven from 50 °C to 60 °C for 24 h. |

*S4.2. General inventory of materials, reagents, and equipment for carrageenan extraction*

*S4.2.1. Materials*

**▪** Lab plastic tray (Step 1).

**▪** Absorbent paper (Steps 1 and 2).

**▪** Spatula (Steps 3 and 4).

**▪** Stainless steel scissors (Steps 1 and 2).

**▪** Cutting board (Steps 1 and 2).

**▪** Glass beaker (Steps 4, 5, 6, 9, 10, and 11).

**▪** Round bottom flask (Step 8).

**▪** Stir bars (Steps 4, 5, and 11).

**▪** Mortar and pestle (Step 33) ! Warning Other procedures or equipment can be considered to grind the material.

**▪** Cloth (Step 7).

**▪** Glass fiber (Step 7).

**▪** Büchner flask (Step 7).

**▪** Büchner funnel (Step 7).

**▪** Petri dish (Steps 10 and 12).

*S4.2.2. Reagents*

**▪** Acetone (C_3_H_6_O) (Step 4).

**▪** Methanol (CH_3_OH) (Step 4).

**▪** Sodium hydroxide (NaOH) (Step 5).

**▪** Hydrochloric acid (HCl) (Step 6).

**▪** Ethanol (C_2_H_5_OH) 96 % (Step 9).

**▪** Absolute ethanol (C_2_H_5_OH) 99 % (Step 11).

*S4.2.3. Equipment*

**▪** Analytical balance (Steps 2 and 4).

**▪** Magnetic stirrer with heater system (Steps 4, 5, and 11).

**▪** Rotary evaporator (Step 8).

**▪** pH meter (Step 6).

**▪** Vacuum pump (Step 7).

**▪** Oven (Steps 2 and 12).

**S5. Ulvan**

*S5.1. Extraction method*

**Table S10.** Comprehensive ulvan extraction protocol: a Step-by-Step Guide.

| **Step** | **Description** |
| --- | --- |
| ◷ TIMING ~9 days (6 hours active, 6 days of the process, and 3 days minimum on freeze-drier) | |
| 1 | Weight 50 g of dried green seaweed powder, carefully stored and milled at low temperatures (using liquid nitrogen).  ! Warning The appropriate safety material to manage liquid nitrogen. Avoid prolonged exposition of the dry algae to prevent moisture absorption that compromises its quality over time. |
| 2 | Weight 25 g of the milled and filtered algae directly to a thimble that is then placed in the Soxhlet system with the necessary volume of dichloromethane (200 mL to 500 mL).  ! Warning The soxhlet extraction system should be assembled inside a chemical hood and managed carefully during the process. If the thimble is small, adjust it to the correct height (for instance, with cotton). It should be higher than the exit.  ! Warning Dichloromethane is an organochloride compound requiring extra caution due to its known hazards. It should also be correctly discarded in a container for halogenated residues. |
| 3 | The thimble content is transferred to a flask for solvent evaporation inside the chemical hood overnight. |
| 4 | The sample is transferred to a round-bottom flask with water and ethanol; 1:4 (500 mL per 25 g). A standard glass apparatus for extraction in water is assembled inside the chemical hood with a stirrer, a thermometer, and a condenser.  ! Advice The temperature should be maintained between 75-90 ºC, preferably 80 °C. Ulvan is extracted by water due to its solubility in water. |
| 5 | The resulting biomass is subject to a repetition of steps 2 to 4. |
| 6 | The resulting aqueous suspension extract is filtrated (using a cotton fabric) and centrifuged at 7000 x g at 4 °C.  ‼ Caution The portion that remains in the filter is discarded. |
| 7 | The sample goes to the vacuum rotary evaporator equipment to concentrate the sample (78 °C for about 3 h). |
| 8 | The purifications then start by subjecting samples to enzymatic reactions containing α-amylase and proteinase K (37 ºC, 30 min), stopping with heat, and then activating charcoal (15 min). Next, the residues are removed with two centrifugation cycles (10 000 x g) and filtration. |
| 9 | The sample is then placed in dialysis membranes overnight to remove impurities. |
| 10 | The sample is recovered and concentrated at the vacuum drying equipment, and then ethanol (50 mL) is added to precipitate the ulvan. Finally, the ethanol is evaporated in the oven, and then ulvan is solubilized in 300 mL of water.  ! Advice An ultrafiltration unit can be used to fractionate the sample by defined sizes at this stage. |
| 11 | The solution is divided into smaller portions, frozen at -80 ºC, and freeze-dried. |
| 12 | The dried sample is stored at 4 °C in an environment with low moisture.  ! Advice Avoid prolonged exposure to the air since the ulvan samples are highly hygroscopic. |

*S5.2. General inventory of materials, reagents, and equipment for ulvan extraction*

*S5.2.1. Materials*

**▪** Mortar and pestle (Step 1).

**▪** Safety equipment to manage liquid nitrogen (Step 1).

**▪** Soxhlet extraction thimble (Step 2).

**▪** Round bottom flask (step 4) with lid and three outlets (for the condenser, stirrer, and thermostat/thermometer).

**▪** Glass condenser (Step 4).

**▪** Stir bars (Steps 2, 4, 5, 8, and 9).

**▪** Thermostat/thermometer (Step 4).

**▪** Clean cotton fabric (Steps 6 and 8).

**▪** Spatula (Steps 1, 2, 6, and 8).

**▪** Stainless steel scissors (Steps 1 and 2).

**▪** Cutting board (Steps 1 and 2).

**▪** Glass beaker (Steps 8 and 9).

**▪** Büchner flask (Steps 6 and 8).

**▪** Büchner funnel (Steps 6 and 8).

*S5.2.2. Reagents*

**▪** Liquid nitrogen (Step 1).

**▪** Dichloromethane (Step 2).

**▪** Ethanol (C_2_H_5_OH) 96 % (Steps 4 and 10).

**▪** α-amylase (Step 8).

**▪** Proteinase K (Step 8).

*S5.2.3. Equipment*

**▪** Analytical balance (Steps 1 and 2).

**▪** Chemical hood (Steps 2, 3, and 4).

**▪** Magnetic stirrer with heater system (Steps 2, 4, 5, 8, and 9).

**▪** Rotary evaporator (Steps 7 and 10).

**▪** Centrifuge (Steps 6 and 8).

**▪** Freeze dryer (Step 11).

**S6. Chondrotin sulfate**

*S6.1. Extraction method*

**Table S11.** Comprehensive chondroitin sulfate extraction protocol: a Step-by-Step Guide.

| **Step** | **Description** |
| --- | --- |
| ◷ TIMING ~3 days (1 day active with a total time of 3 days in treatments) | |
| 1 | Dried cartilage (10 g), previously cut into small portions (5 cm), is extracted with 100 mL of boiling water for 6 h. |
| 2 | Then it is enzymatically digested, and the reaction is stopped by heating at 70 °C for 15 min. The enzymatic reaction could be performed under different approaches:   1. The pancreatic enzyme at pH 8.5 and 53 °C for 6 h. To stop this reaction is also needed to acidify to pH 6 (besides the increased temperature). 2. Different enzymes (neutrase, alcalase, papain, bromelain, and acid protease) are used under their optimum reaction conditions for 4 h . |
| 3 | The reaction product is centrifuged at 2000 x g for 10 min.  ! Warning The supernatant is the enzymatic extract. |
| 4 | The supernatant is precipitated with two volumes of ethanol (95 % v/v) to eliminate impurities. |
| 5 | The sediment is dissolved in hydrogen peroxide: water (1:100 v/v) and hydrolyzed at pH 10 and 60 °C for 4 h. |
| 6 | Steps 3 and 4 are repeated.  ! Warning A chromatographic technique followed by dialysis could replace the purification steps (3-6). It starts by adjusting the pH of the enzymatic reaction product to 4 (using HCl 6 M) loaded onto a column containing 50 mL of DEAE-Sepharose fast-flow resin. CS is then washed from the resin (NaCl 1.0 M; 1 mL/min) and precipitated with three volumes of ethanol (95 % v/v). |
| 7 | The product is dried in the oven at 105 °C for 30 min. |

*S6.2. General inventory of materials, reagents, and equipment for chondroitin sulfate extraction*

*6.2.1. Materials*

**▪** Deionized water (Step 1).

**▪** Round bottom flask (step 1) with lid and three outlets (for the condenser, stirrer, and thermostat /thermometer).

**▪** Glass condenser (Step 1).

**▪** Stir bars (Steps 1, 2, 5, and 6).

**▪** Thermostat/thermometer (Step 1).

**▪** Spatula (Steps 1, 2, 4, 5, 6, and 7).

**▪** Stainless steel scissors (Step 1).

**▪** Scalpel (Step 1).

**▪** Cutting board (Step 1).

**▪** Glass beaker (Steps 2 and 5).

*S6.2.2. Reagents*

**▪** Neutrase (2.0 × 10^5^ U/g) (Step 2)

**▪** Alcalase (2.0 × 10^5^ U/g) (Step 2)

**▪** Papain (6.5 × 10^5^ U/g) (Step 2)

**▪** Bromelain (5.0 × 105 U/g) (Step 2)

**▪** Pancreatic enzyme (4.0 × 10^3^ U/g) (Step 2)

**▪** Acid protease (5.0 × 10^4^ U/g) (Step 2)

**▪** Ethanol (C_2_H_5_OH) 95 % v/v (Steps 4, and 6)

*S6.2.3.. Equipment*

**▪** Analytical balance (Steps 1, 2 and 7).

**▪** Chemical hood (Step 1).

**▪** Magnetic stirrer with heater system (Steps 1, 2, and 5).

**▪** Centrifuge (Steps 3 and 6).

**▪** Oven (Step 7).

**S7. Hyaluronic acid**

*S7.1. Extraction method*

**Table S12.** Comprehensive hyaluronic acid extraction protocol: a Step-by-Step Guide.

| **Step** | **Description** |
| --- | --- |
| ◷ TIMING ~1 week (2 days active and 3 drying periods at 60 ºC) | |
| 1 | The raw materials of interest are cleaned from the rest of the tissue, washed, and cut into small pieces.  ! Advice There is no standard cleaning process in this step. This methodology can be adapted according to the source anatomy. |
| 2 | The raw material is defatted using acetone under stirring. Then, it is homogenized with a new acetone solution and dried at 60 ºC for 24 h.  ! Advice The reaction time can be optimized according to the raw material used. |
| 3 | The clean raw material is solubilized (1 g/20 mL) 100 mM sodium acetate buffer (pH 5.5), comprising 5 mM EDTA and cysteine. |
| 4 | 100 mg of papain is added per gram of biomass and then incubated at 60 ºC for 24 h under stirring.  ! Advice The enzyme used in this step can be changed (e.g., pepsin, pronase, or trypsin ) to optimize the extraction process. |
| 5 | The mixture is boiled for 10 minutes and then centrifuged at 5000 × g for 15 minutes to recover the supernatant. |
| 6 | To precipitate the HA, 3 volumes of ethanol saturated with sodium acetate should be added to the supernatants and left for 24 h at 4 ºC.  ! Advice Different precipitation solutions are adapted in each extraction technique. |
| 7 | The precipitate is recovered by centrifugation at 5000 × g for 15 min and dried at 60 ºC for 6 h.  ◼ Pause Point The dry material can be stored at RT indefinitely. |
| 8 | After this step, the percentage (%) extraction yield of HA can be determined as the ratio between the final product (after drying) and the initial product (weight of by-products biomass, preferably dry) using the following **Equation S6.**  $Extraction yield of HA \%=\frac{Weight of HA}{Weight of biomass}g\times100$  **Equation S6.** |

*S7.2. General Inventory of materials, reagents, and equipment for hyaluronic acid extraction*

*S7.2.1. Materials*

**▪** Stainless steel scissors (Step 1).

**▪** Scalpel (Step 1).

**▪** Cutting board (Step 1).

**▪** Glass or plastic beaker 5L (Step 2-6).

**▪** Stir bars (Steps 2-6).

**▪** Spatula (Steps 3, 4, and 6).

**▪** pH paper (Step 3).

**▪** Centrifugal tubes 50 mL (Steps 5 and 7).

**▪** Strainer (Step 1).

**▪** Glass petri dish (Steps 2, 4, and 7).

*S7.2.2. Reagents*

**▪** Acetone (C_3_H_6_O) (Step 1).

**▪** Ethanol (C_2_H_5_OH) (Step 6).

**▪** Sodium acetate (C_2_H_3_NaO_2_) (Steps 3 and 6).

**▪** EDTA (C_10_H_16_N_2_O_8_) (Step 3).

**▪** Cysteine (Step 3).

**▪** Papain (Step 4).

*S7.2.3. Equipment*

**▪** Analytical balance (Steps 3, 4, 6, and 8).

**▪** Oven (Steps 2, 4, and 7).

**▪** Magnetic stirrer with heater system (Steps 2-6).

**▪** 4 ºC chamber or refrigerator (Step 6).

**▪** Centrifuge (Steps 5 and 7).

**▪** pH meter (Step 3).

**S8. Alginate**

*S8.1. Extraction method*

**Table S13.** Comprehensive alginate extraction protocol: a Step-by-Step Guide.

| **Step** | **Description** |
| --- | --- |
| ◷ TIMING ~1 week (2 days active and 2 days dry) | |
| 1 | Dried brown algae (10 g) are milled and treated with a sulfuric acid solution (500 mL, 0.2 N);  ‼ Caution Sulfuric acid must be carefully handled and inside a chemical wood. |
| 2 | The mixture is maintained in a slow shaker overnight at RT to extract the acid-soluble salts. |
| 3 | Then, a filtration through nylon (0.45 µm pore size) is performed twice with a washing step between (50–100 mL distilled water). |
| 4 | Sodium carbonate solution (500 mL, 1 % w/v) is used to extract the residue by constant stirring at RT overnight. |
| 5 | Filtration through a nylon is performed after diluting the sample with distilled water (1 L). |
| 6 | The biomass extract is recovered by adding NaCl (50 mL, 0.1 %–0.2 %) and then stirring. |
| 7 | Add the stirred mixture, in small volumes, to ethanol (two times the volume of the mixture) while continuously stirring with a glass rod (a precipitate will glue to it). |
| 8 | The obtained precipitate is cleaned with ethanol and dried in the oven (50 °C, 24 h). |
| 9 | A treatment with formaldehyde solution (0.1 % – 0.4 %) for 3–5 h at RT is performed to whiten the alginate, then washed with water. |

*S8.2. General inventory of materials, reagents, and equipment for alginate extraction*

*S8.2.1. Materials*

**▪** Mortar and pestle (Step 1) ! Warning Other procedures or equipment can be considered to grind the material.

**▪** Stainless steel scissors (Step 1).

**▪** Glass beaker 1 L (Step 1).

**▪** Nylon membrane with pore diameter 0.45 µm (Steps 3 and 5).

**▪** Deionized water (Steps 3 and 5).

**▪** Glass rod (Step 7).

**▪**Thermostat/thermometer (Step 8).

*S8.2.2. Reagents*

**▪** Sulfuric acid (H₂SO₄) (Step 1).

**▪** Sodium carbonate (Na₂CO₃) (Step 4).

**▪** Sodium chloride (NaCl) (Step 6).

**▪** Ethanol 95 % (C_2_H_5_OH) (Steps 7 and 8).

**▪** Formaldehyde (CH_2_O) (Step 9).

*S8.2.3. Equipment*

**▪** Analytical balance (Step 1).

**▪** Magnetic stirrer with heater system (Steps 2, 4, and 6).

**▪** Oven (Step 8).

**S9. Agar and agarose**

*S9.1. Conventional extraction procedure*

**Table S14.** Comprehensive agar extraction protocol: a Step-by-Step Guide.

| **Step** | **Description** |
| --- | --- |
| ◷ TIMING ~2-3 weeks (no less than 6 days in pre-treatment, 8 hours active, 2 days dry, and no less than 4 days for syneresis procedure) | |
| 1 | The raw macroalgae must be cleaned and washed numerous times with distilled water to remove other biological materials such as macro- and micro-algae from other species, salts, sand, and small animals. |
| 2 | Cut the materials into small pieces. |
| 3 | Let the macroalgae dry in an oven at 90 °C for 24 h minimum. Then, the percentage of moisture contents can be determined using **Equation S7**. $\text{Moisture contents (\%) = }\frac{m_{d}\text{ - }m_{m}}{m_{m}}\text{ }\times\text{ 100}$  **Equation S7.**  where m_m_ is the dried weight mass, and m_d_ is the initial weight mass of the macroalgae. |
| 4 | Pre-treatment in alkaline solution. Add twenty grams (20 g) into a glass beaker containing 75 mL of 6 % (w/w) of NaOH, under stirring for 6-7 days.  ! Warning This step results in agar with superior "gel strength" due to its capacity to eliminate the sulfated groups and increase the 3,6-anhydrogalactose contents. These chemical modifications to the agar structure improve the gelling properties of the final product and increase its commercial value. |
| 5 | Wash the macroalgae with 0.03 M sulfuric acid (H_2_SO_4_) solution.  ! Warning This step is essential to neutralize the pH of the resulting mixture, and it is fundamental to obtain a high yield in the extractions. |
| 6 | Hot water extraction. Add the neutralized biomass into a glass beaker containing distilled water at 100 °C under continuous stirring for 2 h and a half. |
| 7 | Wash the material with bleach to achieve a bleaching treatment. (Optional step)  This step obtains a lighter shade material which is attractive for a commercial approach. |
| 8 | The resulting extracted solution is filtrated using filter nylon into a filtration system at RT.  ! Warning The portion retained by the filter is discarded. |
| 9 | In order to promote the gelation, the solution is left at RT for several hours and then placed in a fridge for 24 h to increase the consistency of the gel. |
| 10 | Freeze and thaw the extracted material several times (syneresis-based) to separate the residual water and dissolved salts from the agar. |
| 11 | Wash the material with absolute ethanol. |
| 12 | Dry the agar in an oven at 60 °C for 24 h. The percentage (%) yield extraction of agar can be calculated using **Equation S8**. $\text{ Extraction yieldof agar in \% = }\frac{\text{weight of dry agar}}{\text{weight of dry macroalgae}}\text{ }\times\text{ 100}$  **Equation S8.** |
| 13 | Grind the dry agar using a mortar and pestle. After that, the material can be stored at RT. |

*S9.2. Conventional extraction procedure*

**Table S15.** Comprehensive extraction protocol to obtain agarose from agar: a Step-by-Step Guide.

| **Step** | **Description** |
| --- | --- |
| TIMING ~2 days (3-4 hours active and 24 hours dry) | |
| 1 | Dissolve 3 % of the extracted agar into a glass beaker containing 0.05 mol/L of NaCl at 70 °C under constant stirring for 10 min. |
| 2 | Add 600 g of polyethylene glycol (PEG) slowly into the hot agar solution. |
| 3 | Centrifugate the solution at 18500 x g for 15 minutes (discard the supernatant). |
| 4 | Wash twice (2x) the precipitated material in a hot solution containing 25 % PEG in 0.1 mol/L of NaCl. |
| 5 | Wash the precipitate extensively with 0.1 mol/L NaCl solution.  ! Warning This washing procedure must be performed until no darker brown color is observed due to potassium iodide (I-KI) in the solution. |
| 6 | Wash the precipitate several times with 70 % ethanol. |
| 7 | Dry the agar in an oven at 60 °C for 24 h. The percentage (%) yield obtained from agarose can be calculated using **Equation S9**. $\text{Yield of obtaining agarose from agar in \% = }\frac{\text{weight of dry agarose}}{\text{weight of dry agar}}\text{ }\times\text{ 100}$  **Equation S9.** |

*S9.3. General inventory of materials, reagents, and equipment for agar and agarose extraction*

*S9.3.1. Materials*

**▪** Cutting board (Steps 1 and 2 of extraction method).

**▪** Stainless steel scissors (Steps 1 and 2 of extraction method).

**▪** Glass beaker (Steps 4, 5, 6, 7, and 11 of extraction method, and Steps 1, 2, 4, 5, and 6 in purification).

**▪** Stir bars (Steps 4, 5, 8, and 9 of extraction method, and Steps 1, 2, 4, and 5 in purification).

**▪** Büchner flask (Step 8 of extraction method).

**▪** Büchner funnel (Step 8 of extraction method).

**▪** Nylon filter (step 8 of extraction method)

**▪** Petri dishes (Step 12 of extraction method, and Step 7 in purification).

**▪** Spatula (Steps 3 and 12 of extraction method, and Steps 1, 2, 4, and 5 in purification).

**▪** Mortar and pestle (Step 13 of extraction method).

*S9.3.2. Reagents*

**▪** Sodium hydroxide (NaOH) (Step 4 of extraction method).

**▪** Sulfuric acid (H_2_SO_4_) (Step 5 of extraction method).

**▪** Bleach (Step 7 of extraction method).

**▪** Ethanol (C_2_H_5_OH) 96 % (Step 11 of extraction method).

**▪** Sodium chloride (NaCl) (Steps 1, 4, 5 in purification).

**▪** Polyethylene glycol (PEG) (Steps 2, 5 in purification).

**▪** Potassium iodide (I-KI) (Step 5 in purification).

**▪** Ethanol (C_2_H_5_OH) 70 % (Step 6 in purification).

*S9.3.3. Equipment*

**▪** Oven (Steps 3 and 12 of extraction method, and Step 7 in purification).

**▪** Analytical balance (Steps 3, 4, 5, 6, and 12 of extraction method, and Steps 1, 2, 4, and 5 in purification).

**▪** pH meter (Step 5 of extraction method).

**▪** Air pump (Step 8 of extraction method).

**▪** Chemical hood (Step 5 of extraction method).

**▪** Magnetic stirrer with heater system (Steps 4, 5, 6, 7, and 11 of extraction method, and Steps 1, 2, 4, 5, and 6 in purification).

**▪** Fridge (Step 10 of extraction method).

**▪** Freeze (Step 10 of extraction method).

**▪** Centrifuge (Step 3 of purification).
